# Stereoselective hydrogen isotope exchange on nicotinamide cofactors through flavoenzyme microscopic reversibility

**DOI:** 10.1039/d6sc01003b

**Published:** 2026-04-24

**Authors:** Christopher W. Otun, Michael Yuen, Harry J. Spacey, Carlo Bawn, Matthew J. Cliff, Francesco Falcioni, Ryan A. Bragg, Charles S. Elmore, Sam Hay, Jack S. Rowbotham

**Affiliations:** a Manchester Institute of Biotechnology, Department of Chemistry, University of Manchester Manchester M1 7DN UK jack.rowbotham@manchester.ac.uk; b Early Chemical Development, Pharmaceutical Sciences, R&D, AstraZeneca Cambridge UK; c Early Chemical Development, Pharmaceutical Sciences, R&D, AstraZeneca Gothenburg Sweden

## Abstract

We describe a panel of flavoenzymes with the ability to catalyse stereoselective hydrogen isotope exchange (HIE) between ^2^H_2_O (D_2_O) and reduced nicotinamide cofactors, enabling a simple and redox-neutral route to deuterated NAD(P)H isotopomers. In screening the FMN- and FAD-dependent enzymes, which have diverse native functions, we identified catalysts (many of which are commercially available) that selectively yield the full suite of [4-^2^H]-NAD(P)H stereoisotopomers in a single step. In combining stereo-complementary enzymes, we also identify simple one-pot routes to dideuterated [4-^2^H_2_]-NAD(P)H. The biocatalytic methods provide near-quantitative ^2^H-incorporation for both NADPH and NADH under mild conditions, using readily available ^2^H_2_O as the isotope source. The observed activity indicates a reversible flavoenzyme hydride-transfer cycle, reliant on hydrogen/deuterium exchange on the transiently reduced flavin cofactors. We provide computational analysis to rationalise the stereochemical outcomes of the screened reactions. Finally, preparative-scale syntheses are described that deliver isolated deuterated cofactors in excellent yield and isotopic purity. We envisage this easily implemented procedure will simplify access to these important biochemical compounds for mechanistic studies, and may open up wider ^2^H_2_O-driven biocatalytic deuteration reactions.

## Abstract 2

Redox neutral stereoselective hydrogen isotope exchange on NAD(P)H is shown by a panel of flavoenzymes. This activity enables simple preparation of isotopologues of these important biological cofactors for use in synthesis and mechanistic studies.

## Graphical abstract

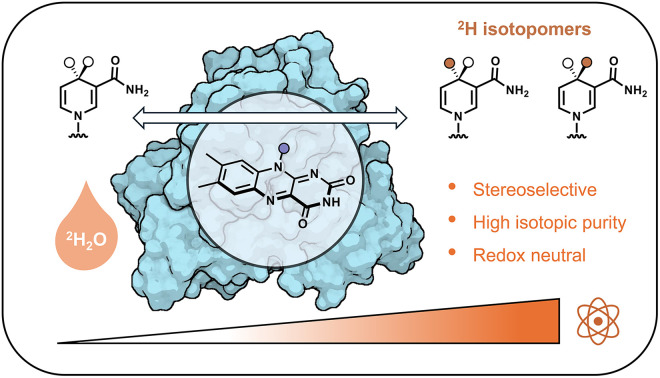

## Introduction

The range of catalytic approaches to hydrogen isotope labelling has been significantly expanded in recent years,^1^ driven in part to support the development of deuterated pharmaceuticals.^2^ Here, biocatalysis offers an attractive option for preparing deuterated compounds with high selectivity and low environmental impact.^3^ Enzymatic strategies for installing deuterium atoms (D or ^2^H) broadly fall into three categories, all of which have seen significant recent developments: (i) reductive deuteration,^4^ (ii) defunctionalisation deuteration (*e.g.* decarboxylation and dehalogenation),^5^ and (iii) hydrogen isotope exchange (HIE).^6^ Of the three, HIE is particularly advantageous as it enables late-stage installation of deuterium atoms by incubating the enzyme and target in ^2^H_2_O – a relatively low cost, readily available, and easy to handle isotope source.

**Fig. 1:**
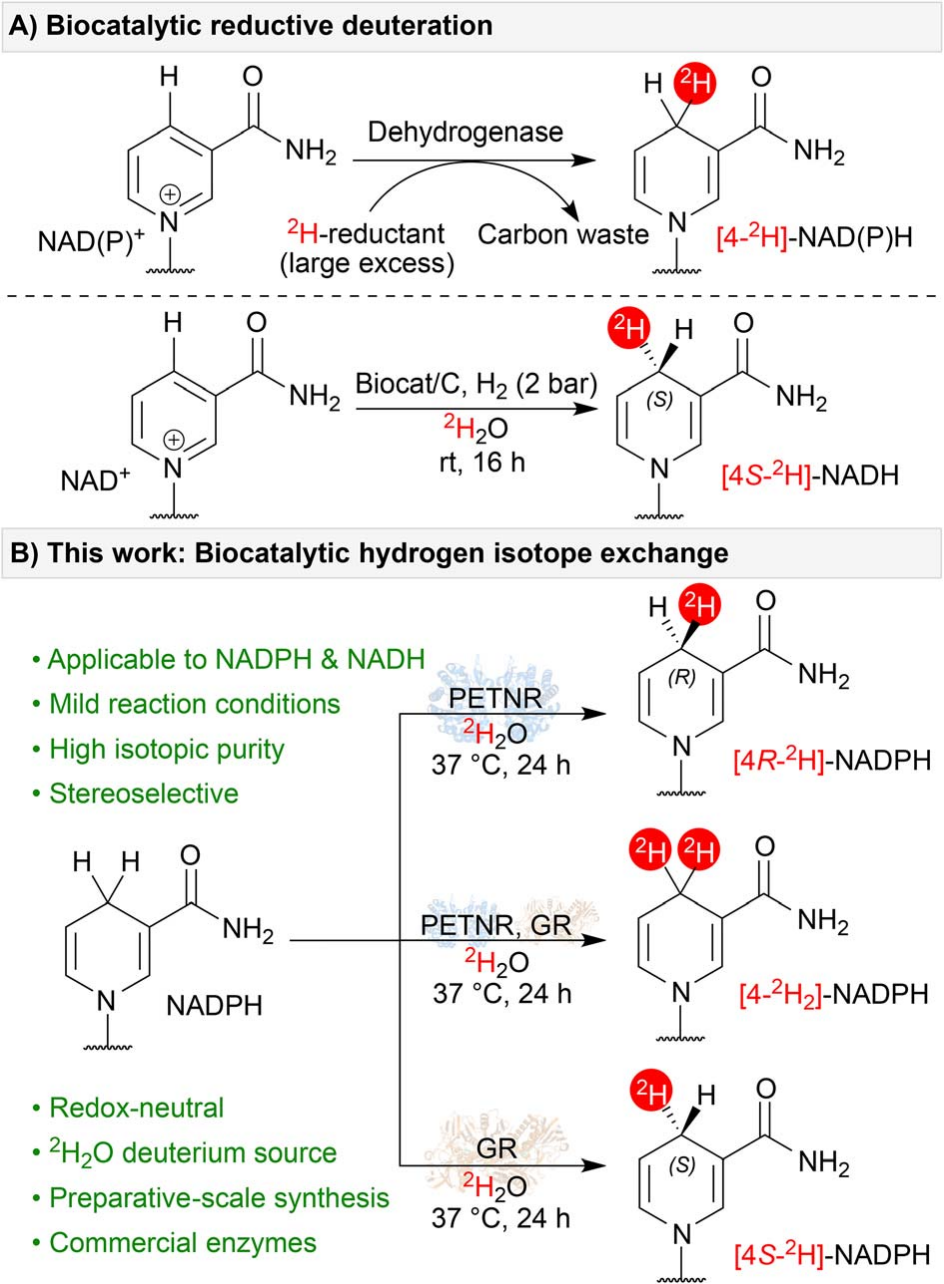
(A) Established examples of [4-^2^H]-NAD(P)H synthesis *via* the biocatalytic reductive deuteration of NAD(P)^+^. (B) This work: stereoselective HIE of NADPH and NADH through flavoenzyme microscopic reversibility.

Many of the recently developed enzymatic HIE approaches exploit microscopic reversibility in biocatalytic mechanisms across a range of enzymes and cofactors. For instance, pyridoxal 5′-phosphate (PLP)-dependent enzymes have been exploited for both the regio- and stereo-selective deuteration of amino acids and derivatives,^6*a*–*c*^ thiamine diphosphate (ThDP)-dependent enzymes can perform aldehyde HIE,^6*d*^ and decarboxylases utilising a prenylated flavin cofactor (prFMN) can exchange alkene C(sp^2^)-^1^H for C(sp^2^)-^2^H.^6*e*^ HIE in relation to NAD(P)H-dependent enzymes has been comparatively underexplored. Given the widespread synthetic applications of [4-^2^H]-NAD(P)H for reductive deuteration (see, for example, previous work by Rowbotham *et al.*, Fig. 1A),^4,7^ a better understanding of redox neutral HIE could be of significant benefit.

In addition to their synthetic utility, isotopomers of [4-^2^H]-NAD(P)H are useful for a variety of applications in kinetic and mechanistic enzymology.^8^ As such, a number of routes for their synthesis have been published.^4*a*,7,9^ These examples, which often involve multiple steps, rely on sacrificial redox chemistry to drive the reductive deuteration of oxidised nicotinamide cofactors (NAD(P)^+^, Fig. 1A). However, in seminal work by Rabinowitz and colleagues wherein *in vivo* isotope distribution pathways were examined,^10^ the authors reported that flavoenzymes can catalyse hydride/deuteride exchange on nicotinamide cofactors in their reduced form. Furthermore, they hypothesised that this exchange should be stereoselective and identified both NADH- and NADPH-active enzymes capable of incorporating ^2^H at the 4-position of the nicotinamide ring. Motivated by these observations, we set out to explore and characterise this novel flavoenzyme activity in more detail and develop a panel of enzymes capable of easily preparing the full suite of [4-^2^H]-NAD(P)H stereoisotopomers (Fig. 1B). Developing this panel will, in turn, open up opportunities for other biocatalytic deuteration strategies that can exploit the very wide array of NAD(P)H-dependent enzymes to prepare selectively deuterated chiral amines and alcohols.

## Results and discussion

### Preparing a panel of flavoenzymes to test for HIE of NAD(P)H

**Fig. 2:**
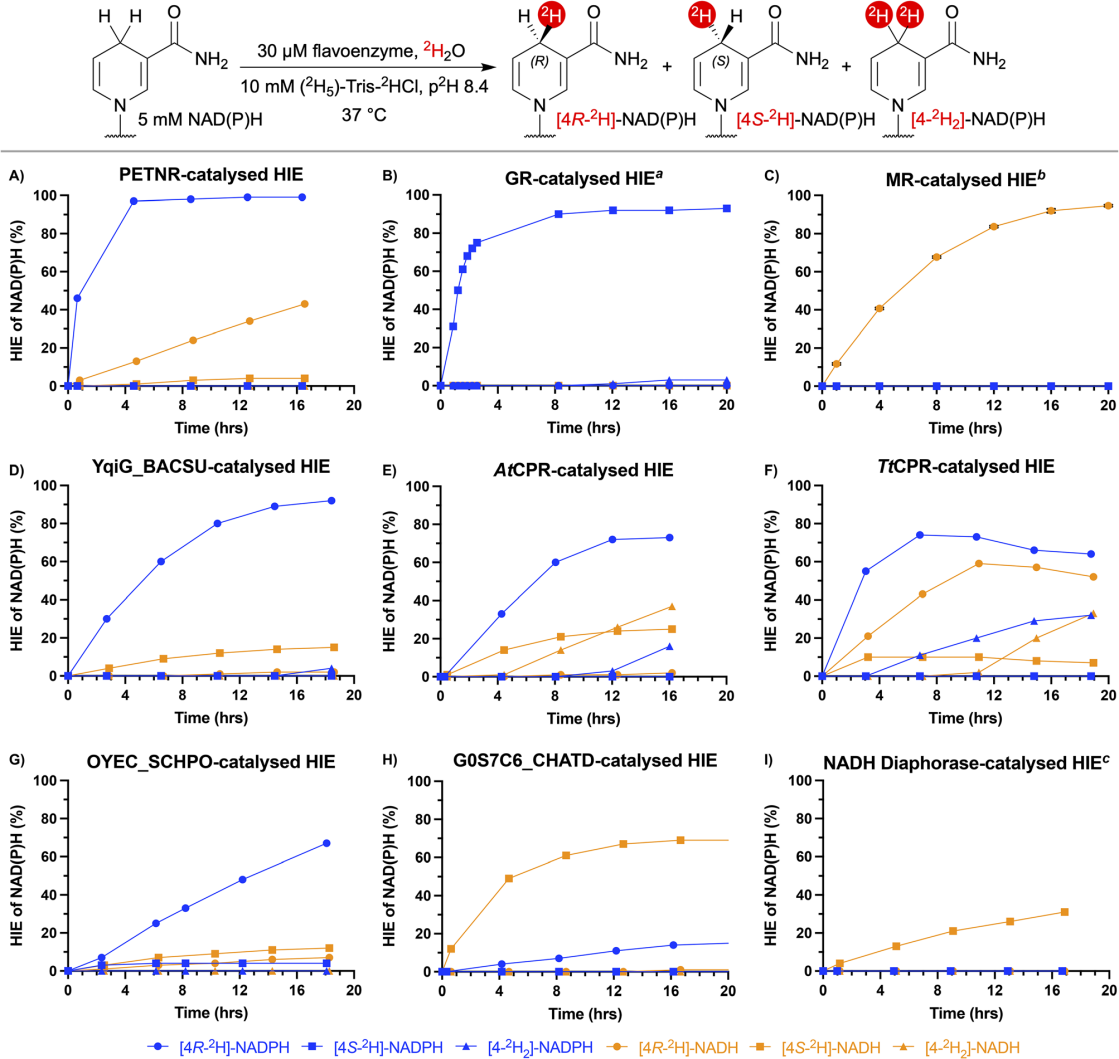
Kinetic profile of flavoenzyme-catalysed HIE of NAD(P)H. Reaction conditions: 5 mM NAD(P)H, 10 mM (^2^H_5_)-Tris-^2^HCl, ^2^H_2_O, p^2^H 8.4, 30 µM flavoenzyme, 37 °C. ^*a*^ Reactions carried out with 15 unit/mL enzyme. ^*b*^ Reaction carried out in triplicate (mean ± SD). ^*c*^ Reactions carried out with 45 unit/mL enzyme. % HIE of NAD(P)H determined by ^1^H NMR (800 MHz, ^2^H_2_O, 310 K).

A panel of flavoenzymes was assembled to screen for HIE activity (Fig. 2). The full details of these varied enzyme candidates are summarised in the supplementary Table S1. Included in the enzyme panel were the reductase domains of various truncated thermostable cytochrome P450 monooxygenases (CYP450): CYP116B65 from *Amycolatopsis thermoflava* (*At*CPR), CYP116B29 from *Thermobispora bispora* (*Tb*CPR) and CYP116B46 from *Tepidiphilus thermophilus* (*Tt*CPR). All of these enzymes are isostructural with the phthalate dioxygenase reductase from *Pseudomonas cepacia* and contain a flavin mononucleotide (FMN) cofactor, as well as a [2Fe-2S] iron-sulfur cluster within their structures.^11^ Also included in the flavoenzyme panel were the FMN-containing enzymes NADPH dehydrogenase C23G7.10c (OYEC_SCHPO) from *Schizosaccharomyces pombe*, commercially available NADH diaphorase from *Clostridium kluyveri*, pentaerythritol tetranitrate reductase (PETNR) from *Enterobacter cloacae*, morphinone reductase (MR) from *Pseudomonas putida*, thermophilic old yellow enzyme (TOYE) from *Thermoanaerobacter pseudethanolicus*, NADH:flavin oxidoreductase from *Bacillus subtilis* (YqiG_BACSU) and NADH:flavin oxidoreductase from *Chaetomium thermophilum* (G0S7C6_CHATD). Previously identified glutathione reductase (GR) from *Saccharomyces cerevisiae* was also included in the panel as a commercially available flavoenzyme which uses flavin adenine dinucleotide (FAD) instead of FMN.^10^

A diagnostic ^1^H NMR signal at *δ* = 2.7–2.9 ppm, corresponding to the redox-active protons at the 4-position of the nicotinamide ring of NAD(P)H, was used to quantify ^2^H-incorporation and to determine the stereoselectivity of the HIE reactions.^9*g*^^1^H NMR signals in the aromatic region were used to quantify NAD(P)H oxidation. Reaction mixtures were analysed by UPLC-MS to confirm monodeuteration by an increase in the molecular weight of NAD(P)H by +1.0. Instances of dideuteration were evidenced by a +2.0 increase in the molecular weight of NAD(P)H compared to commercial standards of NAD(P)H of natural isotopic abundance (see SI sections S5.2.1 and S5.2.2). Additionally, ultraviolet-visible (UV-vis) spectrophotometry was used to further monitor cofactor oxidation or degradation occurring during the HIE reactions by comparing *A*_260_ : *A*_340_ ratios to known standards (see SI sections S5.2.3 and S5.2.4).^12^

To minimise the oxidation or degradation of NAD(P)H in solution, all reactions and analytical samples were prepared under nitrogen. Unless otherwise stated, analytical-scale reaction mixtures containing 5 mM NAD(P)H, 30 µM flavoenzyme and 10 mM (^2^H_5_)-Tris-^2^HCl, p^2^H 8.4 in ^2^H_2_O were incubated at 37 °C (see SI section S3 for detailed methods).

### Initial HIE screening using ^1^H NMR time-course experiments

The initial screen for flavoenzyme HIE activity was performed as a high-throughput ^1^H NMR time-course experiment, with 24 reactions run and monitored in parallel over a 20 hour period (Fig. 2). This system enabled the reactions to be incubated at the desired temperature, with periodic NMR sampling as necessary. In analysing these reactions, it was apparent that PETNR and GR can rapidly catalyse HIE between ^2^H_2_O and NADPH with >99% and 97% ^2^H-incorporation, respectively (Fig. 2A and B). In addition to the faster HIE rates exhibited by PETNR and GR compared to the other flavoenzymes tested, PETNR and GR were found to be both stereoselective and mutually stereo-complementary, with PETNR catalysing the formation of [4*R*-^2^H]-NADPH and GR catalysing the formation of [4*S*-^2^H]-NADPH. The HIE of NADH by PETNR resulted in lower levels of ^2^H-incorporation compared to HIE of NADPH, but stereoselectivity was maintained for the most part (43% [4*R*-^2^H]-NADH after 16 hours, Fig. 2A). GR was unable to catalyse HIE of NADH (Fig. 2B). Conversely, MR proved to be capable of catalysing highly stereoselective HIE of NADH (95% [4*R*-^2^H]-NADH, Fig. 2C), while incubation of MR with NADPH resulted in no ^2^H-incorporation (unsurprisingly, as MR is known to be highly specific for NADH).^13^ Similarly to PETNR, YqiG_BACSU was able to catalyse stereoselective HIE of NADPH (92% [4*R*-^2^H]-NADPH, Fig. 2D). However, the use of NADH as the cofactor resulted in a large decrease in YqiG_BACSU-catalysed HIE activity and was accompanied by a reversal of stereo-preference (15% [4*S*-^2^H]-NADH, Fig. 2D). This reversal of stereoselectivity was also exhibited by *At*CPR, which resulted in the formation of 73% [4*R*-^2^H]-NADPH and 25% [4*S*-^2^H]-NADH in the HIE of NADPH and NADH, respectively (Fig. 2E). More generally, *At*CPR-catalysed HIE of NADPH and NADH proceeded with less stereochemical control compared to most of the other enzymes screened. As such, *A*tCPR formed appreciable amounts of dideuterated product ([4-^2^H_2_]-NAD(P)H) by the end of the time-course experiments (16% [4-^2^H_2_]-NADPH and 37% [4-^2^H_2_]-NADH, Fig. 2E). Of all of the flavoenzymes studied, *Tt*CPR showed the least cofactor specificity, with moderate ^2^H-incorporation levels for monodeuteration reached by the end of the experiment for both NADPH and NADH (64% [4*R*-^2^H]-NADPH and 52% [4*R*-^2^H]-NADH, Fig. 2F). While no reversal of stereoselectivity between *Tt*CPR-catalysed HIE of NADPH and NADH was observed, *Tt*CPR still exhibited a lack of strict stereochemical control, resulting in the formation of 32% [4-^2^H_2_]-NADPH and 33% [4-^2^H_2_]-NADH (Fig. 2F). With 67% [4*R*-^2^H]-NADPH detected at the end of the time-course, OYEC_SCHPO catalysed HIE of NADPH at a rate slower than any of the other flavoenzymes which possess a strong stereochemical preference for [4*R*-^2^H]-NADPH (Fig. 2G). Incubation of NADH with OYEC_SCHPO again resulted in a reversal of stereochemical preference and lower total levels of ^2^H-incorporation compared to the reaction with NADPH (7% [4*R*-^2^H]-NADH and 12% [4*S*-^2^H]-NADH, Fig. 2G). G0S7C6_CHATD showed a preference for NADH, as HIE of NADH resulted in the stereoselective formation of [4*S*-^2^H]-NADH (69%, Fig. 2H). G0S7C6_CHATD-catalysed HIE on NADPH also resulted in a reversal of stereochemistry (15% [4*R*-^2^H]-NADPH, Fig. 2H). Although selective for [4*S*-^2^H]-NADH, NADH diaphorase-catalysed HIE of NADH proceeded at a relatively slow rate, with levels of ^2^H-incorporation reaching 31% by the final measurement of the experiment (Fig. 2I). HIE catalysed by *Tb*CPR and TOYE resulted in relatively low levels of ^2^H-incorporation for both NADPH and NADH. The kinetic profiles for these enzymes can be found in SI section S5.1. All of the promising reactions from the initial screening were repeated for 24 hours to substantiate the identification of flavoenzymes capable of complete deuteration of the redox active hydrogen of NAD(P)H (Table S4).

Incubation of the flavoenzymes with the synthetic nicotinamide cofactor 1-methyl-1,4-dihydronicotinamide (MNAH), which can be used as a biomimetic cofactor in place of NAD(P)H,^14^ resulted in no deuteration in any instance (see SI section S4.3).

Interestingly, the UPLC-UV chromatograms and mass spectra obtained showed that, under some circumstances, the biologically active β-NAD(P)^+^ undergoes anomerisation to result in the formation of biologically inactive α-NAD(P)^+^ (see SI section S4.4 for a more detailed discussion and UPLC-UV peak assignment). However, this was only observed in rare cases where significant NAD(P)H oxidation had occurred, indicating that the α-NAD(P)^+^ might form from unstable mixtures of NAD(P)H and NAD(P)^+^.

### Comparison of observed HIE against known enzyme catalytic parameters

An examination of published kinetic data for some of the flavoenzymes in their native reactions could explain the rates of HIE observed in Fig. 2. For example, glutathione reduction by GR has the NADPH reduction of the FAD cofactor as the rate determining step. However, this reductive half-reaction step occurs relatively rapidly, even at 5 °C (153 s^−1^).^15^ This could explain the particularly fast rate of GR-catalysed HIE of NADPH at 37 °C (Fig. 2B). GR is known to exhibit low *K*_m_ values for NADPH (∼3 – 20 µM, depending on the organism),^15,16^ while *K*_m_ values for NADH tend to be around 100 times higher.^16^ These values are consistent with the cofactor specificity exhibited by GR for NADPH over NADH under the HIE reaction conditions (Fig. 2B).

Reports show that the rate of GR-bound FAD reduction is significantly faster than that of MR-bound FMN reduction (23.4 s^−1^ at 5 °C).^15,17^ The data obtained in this study is consistent with these reported kinetics as GR-catalysed HIE of NADPH (Fig. 2B) occurred at a rate faster than MR-catalysed HIE of NADH (Fig. 2C).

PETNR-catalysed HIE of NADPH occurred at a rate comparable to the GR-catalysed reaction (Fig. 2A), however, reported kinetic constants measured at 5 °C for the reduction of GR-bound FAD are significantly higher than that of PETNR-bound FMN.^15,17*b*,18^ This suggests that the kinetics of flavoenzyme-catalysed HIE between ^2^H_2_O and NAD(P)H cannot be accurately explained by looking at known rate constants for the reductive half-reactions alone. Nevertheless, the cofactor specificity exhibited by PETNR for NADPH over NADH in the HIE reaction is in agreement with reported *K*_m_ values for PETNR (0.08 ± 0.01 mM for NADPH, 1.5 ± 0.7 mM for NADH),^19^ suggesting that the cofactor specificity observed in the native reactions is maintained in the flavoenzyme-catalysed HIE reactions. The difference in *K*_m_ between NADPH and NADH for GR is far greater than that of PETNR,^15,16,19^ indicating that GR is more cofactor specific than PETNR. This trend in specificity is also observed in the HIE reactions (Fig. 2A*versus* 2B).

### Observed non-enzymatic reactivity

Control experiments where NAD(P)H was incubated in ^2^H_2_O in the absence of any enzyme resulted in no ^2^H-incorporation, indicating that the protons at the 4-position of the nicotinamide ring do not freely undergo exchange with ^2^H_2_O (Table S4, entry 1). Experiments where enzyme-free FAD was incubated with NAD(P)H also resulted in no ^2^H-incorporation (Table S4, entry 2).

The ^1^H NMR spectra obtained from the time-course experiments showed that a small proportion of the starting 1,4-NAD(P)H cofactor can undergo slow isomerisation to form redox-inactive 1,6-NAD(P)H. It is likely that this reactivity has been overlooked until now, because it is easily missed without the aid of a high field (800 MHz) spectrometer. In addition to unlabelled 1,6-NAD(P)H, our ^1^H NMR analysis revealed the formation of deuterated 1,6-NAD(P)H (assumed to be [6*R*-^2^H]-NAD(P)H from analogy of the peak shape with [4*R*-^2^H]-NAD(P)H). It is probable that, under the standard HIE reaction conditions, isomerisation of 1,4-NAD(P)H proceeds *via* a non-enzymatic hydride transfer from NAD(P)H to NAD(P)^+^.^20^ The observed stereo-preference of the reaction could be explained by the asymmetric conformation adopted by the cofactor in solution. For reactions which efficiently produced [4-^2^H]-NAD(P)H (*e.g.* GR-catalysed HIE of NADPH), levels of 1,6-NAD(P)H were around 2%. The highest levels of 1,6-NAD(P)H (≤10%) were observed only in reactions where significant cofactor oxidation took place (see SI section S4.5 for a more detailed discussion, NMR peak assignment and a scheme depicting the proposed mechanism for 1,4-NAD(P)H isomerisation).

### Synthesis of dideuterated NAD(P)H

**Fig. 3:**
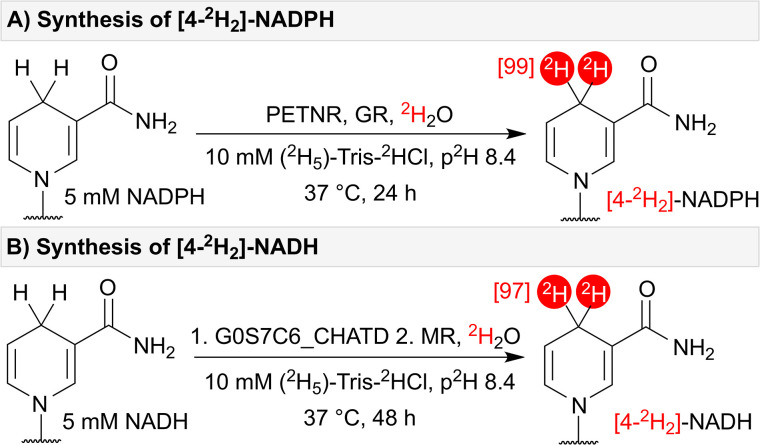
(A) Synthesis of [4-^2^H_2_]-NADPH – catalysed by stereo-complementary enzymes PETNR and GR. (B) Synthesis of [4-^2^H_2_]-NADH – catalysed by stereo-complementary enzymes G0S7C6_CHATD and MR.

Having identified enzymes capable of performing efficient HIE for the synthesis of [4*R*-^2^H]-NADPH (PETNR, OYEC_SCHPO and YqiG_BACSU), [4*S*-^2^H]-NADPH (GR), [4*R*-^2^H]-NADH (MR) and [4*S*-^2^H]-NADH (G0S7C6_CHATD), we envisioned the possibility for complete dideuteration of NAD(P)H by using stereo-complementary flavoenzymes in one-pot. Through this approach, PETNR and GR successfully catalysed the formation of [4-^2^H_2_]-NADPH with 99% ^2^H-incorporation (Fig. 3A). NADH underwent dideuteration in a one-pot, two-step reaction where G0S7C6_CHATD was initially incubated with NADH for 24 hours, followed by the addition of MR to give [4-^2^H_2_]-NADH with 97% ^2^H-incorporation after a further 24 hours of incubation (Fig. 3B). It is worth noting that, for very high levels of deuteration, additional precautions are required to remove adventitious protons such as pre-exchanging the enzymes into ^2^H_2_O buffer, drying and exchanging out labile protons on other salts and reagents, and working in a dry atmosphere.

### HIE mechanism and molecular dynamics

**Fig. 4:**
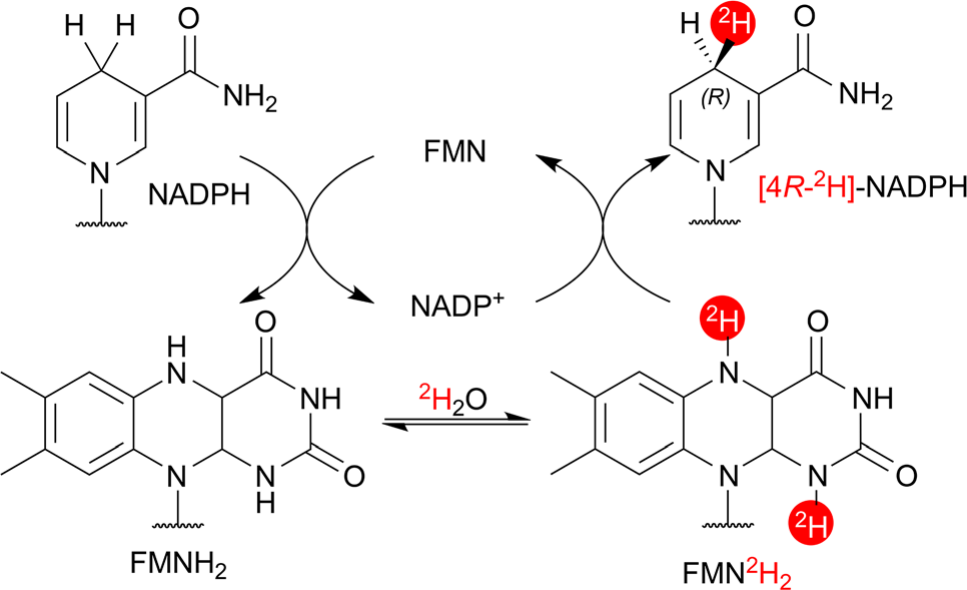
Speculative mechanism for *At*CPR-catalysed HIE of NADPH.^10^

The proposed mechanism for flavoenzyme-catalysed HIE proceeds *via* microscopic reversibility in the half-reaction between NAD(P)H and the flavin cofactor (Fig. 4).^10^ NAD(P)H first reduces the flavin *via* hydride transfer, forming a labile N5–H bond that can exchange readily with ^2^H_2_O.^17*b*^ The resulting deuterated reduced flavin then transfers deuterium to NAD(P)^+^ (*via* deuteride transfer) to generate [4-^2^H]-NAD(P)H.

Truncation of *At*CPR for the removal of the [2Fe-2S] cluster was performed to assess whether the [2Fe-2S] clusters in the CPRs affect HIE activity (see SI section S4.6). No significant difference in NADPH HIE activity was observed between truncated *At*CPR (*At*CPR-FMN) and full-length *At*CPR, suggesting that the [2Fe-2S] cluster plays no role in the HIE mechanism.

**Fig. 5:**
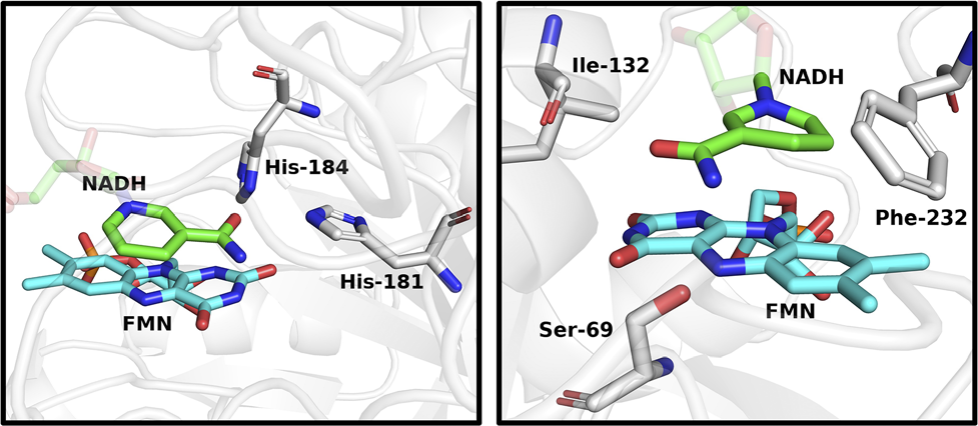
MD simulations of NADH binding poses within the active sites of PETNR and *At*CPR. Representative structures were chosen based on a clustering analysis of the Cα (protein) RMSD. Left: The amide of NADH forms interactions with His-181 and His-184 in PETNR to anchor the nicotinamide ring for stereoselective hydride/deuteride transfer. Right: A lack of interactions between the amide of NADH and nearby residues in *At*CPR enables conformational flexibility for hydride/deuteride transfer with less stereocontrol.

To explore differences in cofactor specificity and stereoselectivity between the flavoenzymes, molecular dynamics (MD) simulations were used to examine interactions of NADH with *At*CPR and PETNR. For *At*CPR, simulations were conducted from two different NADH binding poses that are consistent with either *pro*-(*R*) or *pro*-(*S*) hydride transfer to the flavin. These simulations revealed a lack of H-bonding interactions between the nicotinamide ring and the enzyme active site (Fig. 5 right for binding pose consistent with *pro*-(*S*) hydride transfer. See Fig. S6 for binding pose consistent with pro-(*R*) hydride transfer). Conversely, simulations of PETNR revealed interactions between the amide group of the nicotinamide ring and two histidine residues (His-181 and His-184, Fig. 5 left. See Fig. S7 for RMSD data).^21^ Experimental studies have shown that PETNR catalyses *pro*-(*R*) hydride transfer from NAD(P)H to alkene substrates, a stereo-preference likely governed by these nicotinamide interactions.^8*c*,19,22^ In contrast, the same interactions appear to be absent in *At*CPR, enabling either *pro*-(*R*) or *pro*-(*S*) hydride transfer from the nicotinamide ring. This analysis supports the switch in stereoselectivity observed between *At*CPR-catalysed HIE of NADPH and NADH (Fig. 2E). Since the 2′-phosphate of NADPH is distal from the active site, identifying specific H-interactions with this group is more difficult.^23^ The 2′-phosphate may constrain the cofactor in a binding orientation that favours *pro*-(*R*) hydride transfer, whereas the absence of this group in NADH may allow greater conformational flexibility and permit a binding pose that results in the formation of [4*S*-^2^H]-NADH. The conformational flexibility of NADH in the active site of *At*CPR could also explain why significant levels of [4-^2^H_2_]-NADH were detected as, despite a preference for *pro*-(*S*) deuteration, deuteride transfer could occur on either face of the nicotinamide ring (Fig. 2E).

With the mechanism of flavoenzyme microscopic reversibility in mind, it is worth addressing the means by which the considerable generation of [4-^2^H_2_]-NADPH in the *At*CPR- and *Tt*CPR-catalysed HIE reactions may have arisen. In the time-course reactions, both enzymes initially produced [4*R*-^2^H]-NADPH as the sole product, however as time progressed, [4-^2^H_2_]-NADPH formation was observed, without any emergence of [4*S*-^2^H]-NADPH (Fig. 2E and F). The most plausible explanation for this occurrence may be a kinetic isotope effect (KIE) in the oxidation of [4*R*-^2^H]-NADPH after re-entry into the active sites of *At*CPR and *Tt*CPR (Fig. S8). This KIE may be large enough such that (*S*)-hydride transfer from [4*R*-^2^H]-NADPH to FMN becomes kinetically favourable over (*R*)-deuteride transfer, resulting in the generation of a planar [4-^2^H]-NADP^+^ intermediate. Subsequent deuteride transfer from FMN^2^H_2_ then leads to the formation of [4-^2^H_2_]-NADPH (see Fig. S8 for a proposed reaction scheme).

### Preparative-scale synthesis of [4*R*-^2^H]-, [4*S*-^2^H]- and [4-^2^H_2_]-NADPH

Based on the levels of ^2^H-incorporation observed in the analytical-scale reactions, PETNR and GR were selected for preparative-scale HIE of NADPH. Firstly, a HIE reaction was performed with 25 mg NADPH (0.03 mmol) in the presence of 0.6 mol% PETNR to give [4*R*-^2^H]-NADPH in near quantitative yield and 99% ^2^H-incorporation (as measured by MS). The same was conducted with 34 U GR to afford [4*S*-^2^H]-NADPH in near quantitative yield and 96% ^2^H-incorporation. Finally, 25 mg NADPH was treated with PETNR (0.6 mol%) and GR (34 U) in one-pot to give [4-^2^H_2_]-NADPH in near quantitative yield and 94% ^2^H-incorporation. In all cases of preparative-scale synthesis, deuterated nicotinamide cofactor products were purified by anion exchange chromatography and easily isolated as white solids by lyophilisation.

## Conclusions and outlook

In summary, a panel of flavoenzymes was shown to catalyse regio- and stereoselective HIE between ^2^H_2_O and NAD(P)H. Flavoenzymes were identified for the efficient synthesis of [4*R*-^2^H]-NADPH (PETNR, OYEC_SCHPO and YqiG_BACSU), [4*S*-^2^H]-NADPH (GR) and [4*R*-^2^H]-NADH (MR). [4*S*-^2^H]-NADH could also be generated (G0S7C6_CHATD), albeit with a lower level of ^2^H-incorporation compared to the other NAD(P)H isotopologues mentioned under the conditions that we studied. It is likely that more successful candidates exist for this target, which may become apparent with future research.††For example, Rowbotham *et al*. have previously identified an atom efficient route to [4*S*-^2^H]-NADH using a hydrogenase-driven approach,^7^ and preliminary data indicates that the FMN-centred NAD^+^ reductase domain may also be a suitable candidate for HIE. [4-^2^H_2_]-NAD(P)H was also easily accessed upon incubation of NAD(P)H with the relevant stereo-complementary flavoenzymes in ^2^H_2_O in one-pot. Enzymatic HIE was successfully monitored over time with a parallel ^1^H NMR spectroscopic approach. Molecular docking of NADH in the active site of different candidates provided plausible explanations for the varying cofactor specificity and stereoselectivity of these enzymes. Moreover, the demonstration of preparative-scale enzymatic HIE shows how this method could be employed as a convenient and atom economical method for the preparation of deuterated cofactors to be used in the study of NAD(P)H-dependent enzyme kinetics and mechanisms. Our future work will focus on exploiting the observed activity in synthetic HIE strategies using NAD(P)H-dependent enzymes to prepare valuable deuterated chiral amines and alcohols.

## Author contributions

J. S. R. conceptualised and supervised the work. F. F., R. A. B. and C. S. E. provided industrial supervision and steered the applicative focus of the project. C. W. O., M. Y. and H. J. S. performed protein expression and purification. C. W. O. designed and carried out all deuteration experiments. C. B and M. J. C assisted C. W. O. with the execution of the NMR time-course experiments. M. Y. and S. H. carried out computational work and helped with interpretation. C. W. O. and J. S. R contributed to data analysis. C. W. O. prepared the original manuscript draft, and all authors assisted with the reviewing and editing process.

## Conflicts of interest

There are no conflicts to declare.

## Supplementary Material

SC-017-D6SC01003B-s001

## Data Availability

The datasets supporting this article have been uploaded as part of the SI. Supplementary information (SI) is available. See DOI: https://doi.org/10.1039/d6sc01003b.
