# Supplementary material for: Stereoselective hydrogen isotope exchange on nicotinamide cofactors through flavoenzyme microscopic reversibility
Source: Chem Sci. 2026 Apr 24;17(23):11459–65. doi: 10.1039/d6sc01003b (PMC13147284; doi:10.1039/d6sc01003b)
Supplement: SC-017-D6SC01003B-s001 [file SC-017-D6SC01003B-s001.pdf]

Supplementary Information (SI) for Chemical Science.
This journal is © The Royal Society of Chemistry 2026

Electronic Supplementary Information (ESI)

Stereoselective hydrogen isotope exchange on nicotinamide cofactors through flavoenzyme microscopic reversibility

Christopher W. Otun, Michael Yuen, Harry J. Spacey, Carlo Bawn, Matthew J. Cliff, Francesco Falcioni, Ryan A. Bragg, Charles S. Elmore, Sam Hay and Jack S. Rowbotham*

*Corresponding author: jack.rowbotham@manchester.ac.uk

Table of contents

S1. List of abbreviations ... 3

S2. Supplementary figures and tables ... 5

S3. Methods and materials ... 10

S3.1. General information ... 10

S3.1.1. Preparation of deuterated buffers ... 10

S3.1.2. Analytical instrumentation ... 10

S3.2. Enzymes ... 11

S3.2.1. Commercially available enzymes ... 11

S3.2.2. Enzyme expression and purification ... 11

S3.2.3. Digestion of *At*CPR for the removal of [2Fe-2S] cluster ... 12

S3.2.4. Calculation of enzyme concentration ... 13

S3.2.5. Protein sequences ... 13

S3.3. Biocatalysis ... 15

S3.3.1. General procedure for the ^{1}H NMR (800 MHz) time-course ... 15

S3.3.2. General procedure for the 24-hour HIE reactions ... 16

S3.3.3. Sample preparation and procedure for UPLC-MS analysis ... 16

S3.3.4. Sample preparation for UV-vis spectrophotometric analysis ... 17

S3.3.5. Determination of cofactor proportions by ^{1}H NMR spectroscopy ... 17

S3.3.6. Preparative-scale synthesis of [4*S*-^{2}H]-NADPH ... 19

S3.3.7. Preparative-scale synthesis of [4*R*-^{2}H]-NADPH ... 19

S3.3.8. Preparative-scale synthesis of [4-$^{2}H_2$]-NADPH ... 19

S3.4. Molecular modelling ... 20

S3.4.1. Model setup ... 20

S3.4.2. System equilibration and molecular dynamics ... 21

S4. Supplementary results and discussion ... 25

S4.1. Control experiments in $^{1}H_2O$ ... 25

S4.2. Optimisation of reactivity for full HIE of NAD(P)H redox active hydrogen ... 25

S4.3. HIE of 1-methyl-1,4-dihydronicotinamide ... 27

S4.4. Formation of α-NAD(P)$^{+}$ ... 27

S4.5. Formation of 1,6-NAD(P)H ... 28

S4.6. Truncation of *At*CPR for the removal of [2Fe-2S] cluster ... 32

S5. Supplementary data ... 33

S5.1. HIE reaction time-course ... 33

S5.1.1. Stereochemical outcome of HIE over time ... 33

S5.1.2. ^{1}H NMR (800 MHz) data ... 33

S5.2. 24-hour HIE reactions ... 44

S5.2.1. UPLC-MS data – HIE of NADPH ... 44

S5.2.2. UPLC-MS data – HIE of NADH ... 46

S5.2.3. ^{1}H NMR (400 MHz, $^{2}H_2O$, p^{2}H 8.4, 298 K) and UV-vis data – HIE of NADPH ... 48

S5.2.4. ^{1}H NMR (400 MHz, $^{2}H_2O$, p^{2}H 8.4, 298 K) and UV-vis data – HIE of NADH ... 52

S5.3. Preparative-scale HIE reactions ... 55

S5.3.1. UPLC-MS data ... 55

S5.3.2. UV-vis data ... 56

S5.3.3. ^{1}H NMR data (400 MHz, $^{2}H_2O$, p^{2}H 8.0, 298 K) ... 57

S6. Supplementary References ... 58

S1. List of abbreviations

Abbreviation	Definition
*At*CPR	Cytochrome P116B65 reductase from *Amycolatopsis thermoflava*
*Tb*CPR	Cytochrome P116B29 reductase from *Thermobispora bispora*
*Tt*CPR	Cytochrome P116B46 reductase from *Tepidiphilus thermophilus*
COSY	Correlation spectroscopy
CYP450	Cytochrome P450 monooxygenase
d.e	Diastereomeric excess
FAD	Flavin adenine dinucleotide (oxidised)
$FADH_2$	Flavin adenine dinucleotide (reduced)
FMN	Flavin mononucleotide (oxidised)
$FMNH_2$	Flavin mononucleotide (reduced)
G0S7C6_CHATD	NADH:flavin oxidoreductase from *Chaetomium thermophilum*
GR	Glutathione reductase
HIE	Hydrogen isotope exchange
LC-MS	Liquid chromatography-mass spectrometry
MHz	Megahertz
MNAH	1-methyl-1,4-dihydronicotinamide
MR	Morphinone reductase
NAD^+	Nicotinamide adenine dinucleotide (oxidised form)
$NADP^+$	Nicotinamide adenine dinucleotide phosphate (oxidised form)
NADH	Nicotinamide adenine dinucleotide (reduced form)
NADPH	Nicotinamide adenine dinucleotide phosphate (reduced form)
[4-^{2}H]-NADH	Nicotinamide adenine dinucleotide (reduced form), singly deuterated at 4-position of nicotinamide ring. Unspecified stereochemistry
[4-^{2}H]-NADPH	Nicotinamide adenine dinucleotide phosphate (reduced form), singly deuterated at 4-position of nicotinamide ring. Unspecified stereochemistry
[4*R*-^{2}H]-NADH	Nicotinamide adenine dinucleotide (reduced form), singly deuterated at 4-position of nicotinamide ring. (*R*)-form
[4*R*-^{2}H]-NADPH	Nicotinamide adenine dinucleotide phosphate (reduced form), singly deuterated at 4-position of nicotinamide ring. (*R*)-form
[4S-^{2}H]-NADH	Nicotinamide adenine dinucleotide (reduced form), singly deuterated at 4-position of nicotinamide ring. (*S*)-form

[4S-^{2}H]-NADPH	Nicotinamide adenine dinucleotide phosphate (reduced form), singly deuterated at 4-position of nicotinamide ring. (*S*)-form
[4-$^2H_2$]-NADH	Nicotinamide adenine dinucleotide (reduced form), doubly deuterated at 4-position of nicotinamide ring
[4-$^2H_2$]-NADPH	Nicotinamide adenine dinucleotide phosphate (reduced form), doubly deuterated at 4-position of nicotinamide ring
NMR	Nuclear magnetic resonance
OYEC_SCHPO	NADPH dehydrogenase from *Schizosaccharomyces pombe*
PETNR	Pentaerythritol tetranitrate reductase
TOYE	Thermophilic old yellow enzyme
UV-Vis	Ultraviolet-visible
YqiG_BACSU	NADH flavin oxidoreductase from *Bacillus subtilis*

S2. Supplementary figures and tables

Table S1: Flavoenzymes used in this study.

Enzyme full name	Abbreviation	Enzyme family	Native species	Role in nature	Cofactor(s)
Cytochrome P116B65 reductase	*At*CPR	CYP116	*Amycolatopsis thermoflava*	Facilitates electron transfer to sustain CYP450 catalytic cycle	FMN and [2Fe-2S]
Cytochrome P116B29 reductase	*Tb*CPR	CYP116	*Thermobispora bispora*	Facilitates electron transfer to sustain CYP450 catalytic cycle	FMN and [2Fe-2S]
Cytochrome P116B46 reductase	*Tt*CPR	CYP116	*Tepidiphilus thermophilus*	Facilitates electron transfer to sustain CYP450 catalytic cycle	FMN and [2Fe-2S]
OYEC_SCHPO	OYEC_SCHPO	NADPH dehydrogenase	*Schizosaccharomyces pombe*	Facilitates electron transfer from NADPH	FMN
Pentaerythritol tetranitrate reductase	PETNR	Old yellow enzyme	*Enterobacter cloacae*	Degradation of organic nitrate esters	FMN
Morphinone reductase	MR	Old yellow enzyme	*Pseudomonas putida*	Degradation of opiate alkaloids	FMN
Thermophilic old yellow enzyme	TOYE	Old yellow enzyme	*Thermoanaerobacter pseudethanolicus*	Detoxification of xenobiotics	FMN
NADH diaphorase	NADH diaphorase	NADH:flavin oxidoreductase	*Clostridium kluyveri*	Facilitates electron transfer from NADH to FMN	FMN
YqiG_BACSU	YqiG_BACSU	NADH:flavin oxidoreductase	*Bacillus subtilis*	Facilitates electron transfer from NADH to FMN	FMN
G0S7C6_CHATD	G0S7C6_CHATD	NADH:flavin oxidoreductase	*Chaetomium thermophilum*	Facilitates electron transfer from NADH to FMN	FMN
Glutathione reductase	GR	Class-I pyridine nucleotide-disulfide oxidoreductase	*Saccharomyces cerevisiae*	Reduction of oxidised glutathione to sustain cellular redox homeostasis	FAD

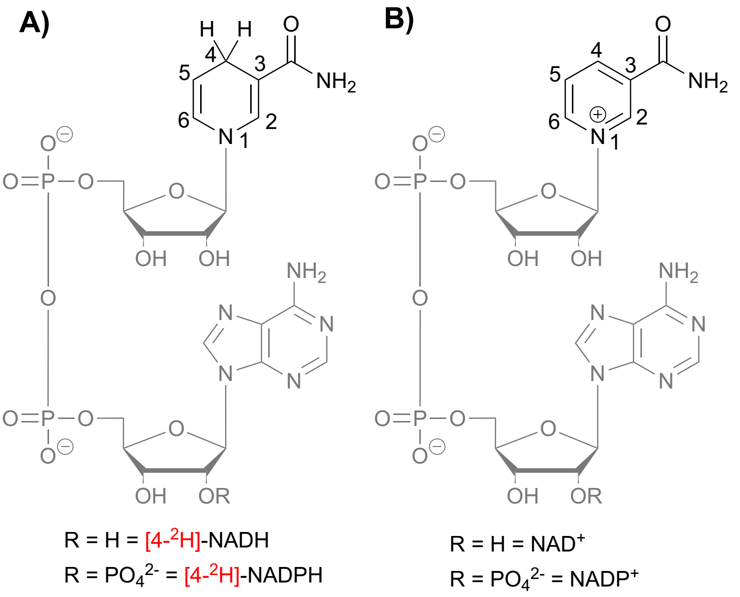

Fig. S1: A) Structure of reduced nicotinamide cofactors NADH and NADPH. **B)** Structure of oxidised nicotinamide cofactors NAD^+ and $NADP^+$.

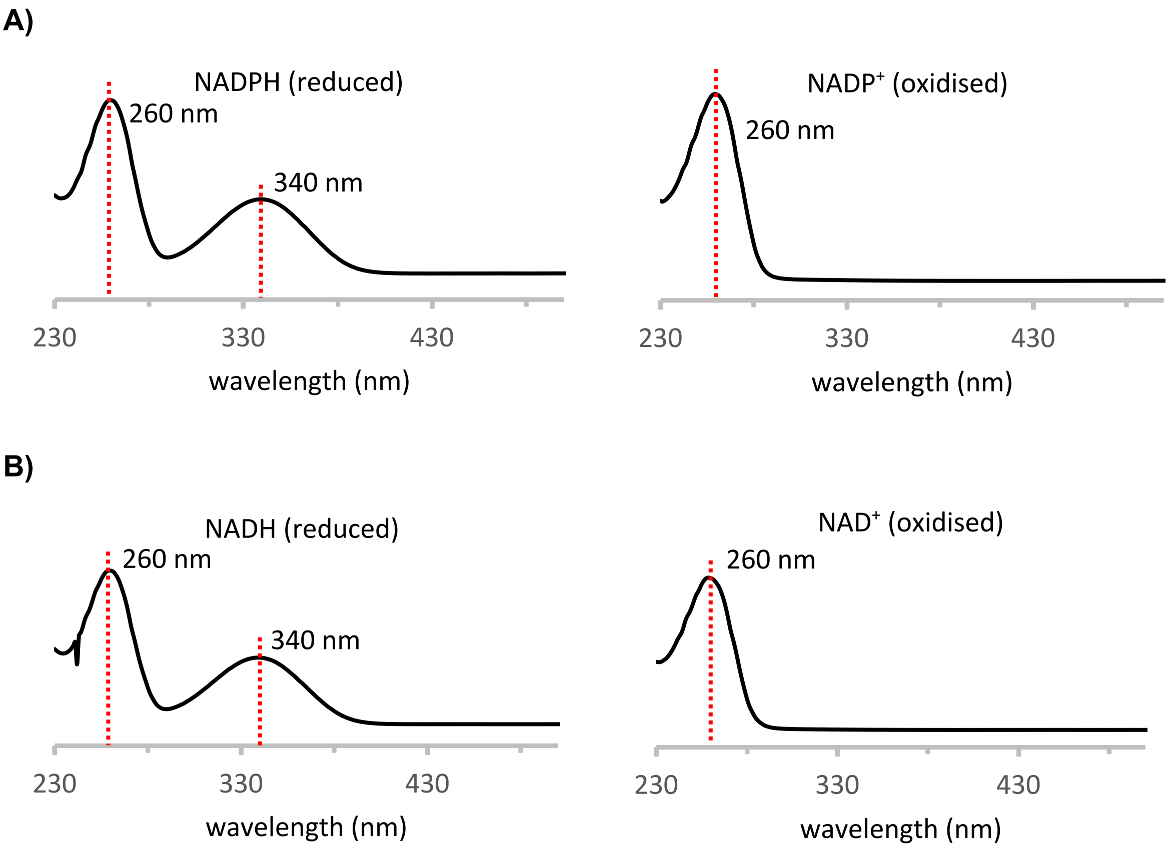

Fig. S2: Ultraviolet-visible spectra for oxidised and reduced NAD(P)H in 10 mM Tris-HCl, pH 8.0. **A)** NADP(H). **B)** NAD(H).

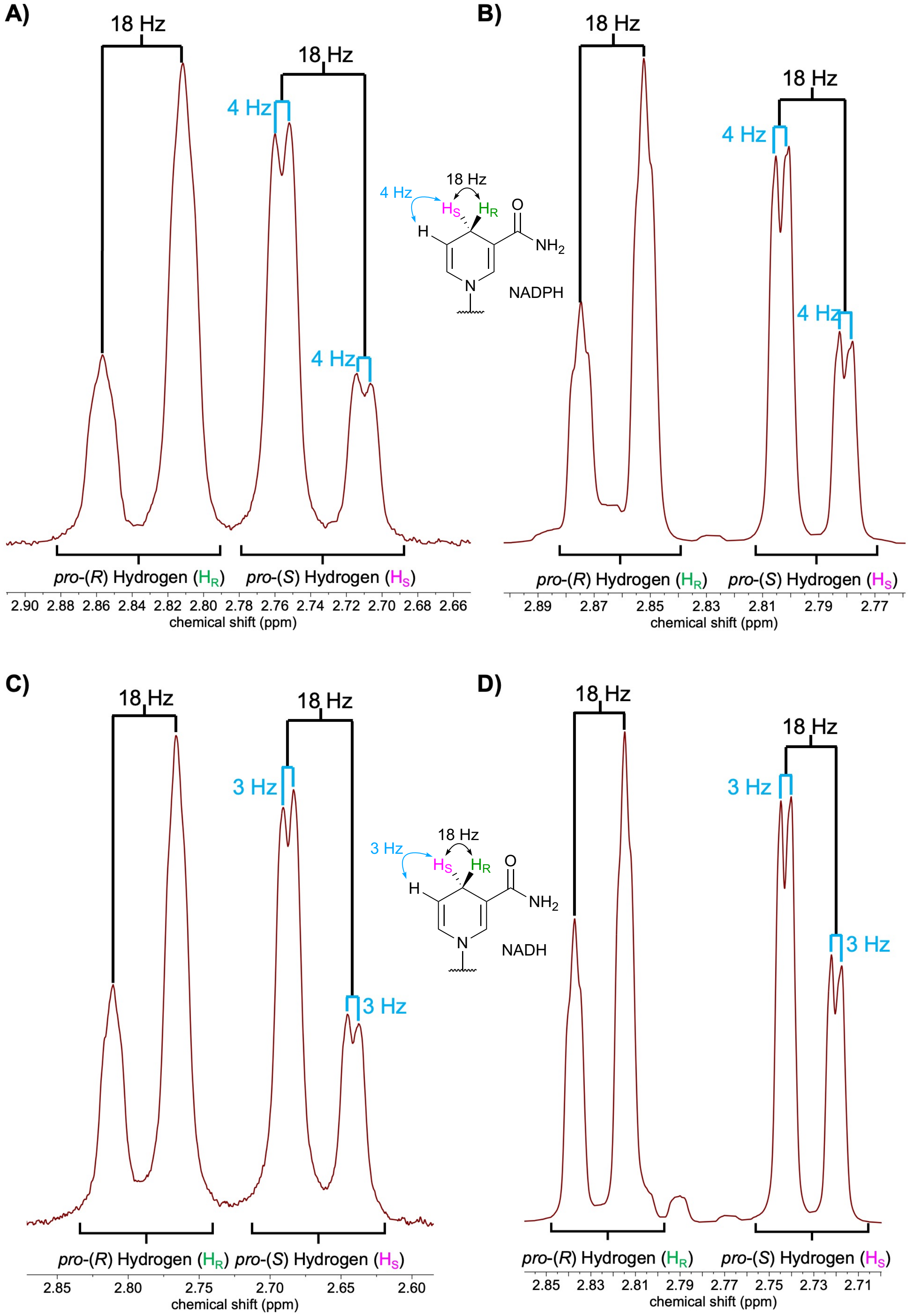

Fig. S3: ^{1}H NMR multiplet and associated *J* coupling for the diagnostic region corresponding to the redox active protons of NAD(P)H in 10 mM Tris-($^2H_5$)-Tris-^{2}HCl, p^{2}H 8.4 at 310 K. **A)** NADPH, 400 MHz. **B)** NADPH, 800 MHz. **C)** NADH, 400 MHz. **D)** NADH, 800 MHz.

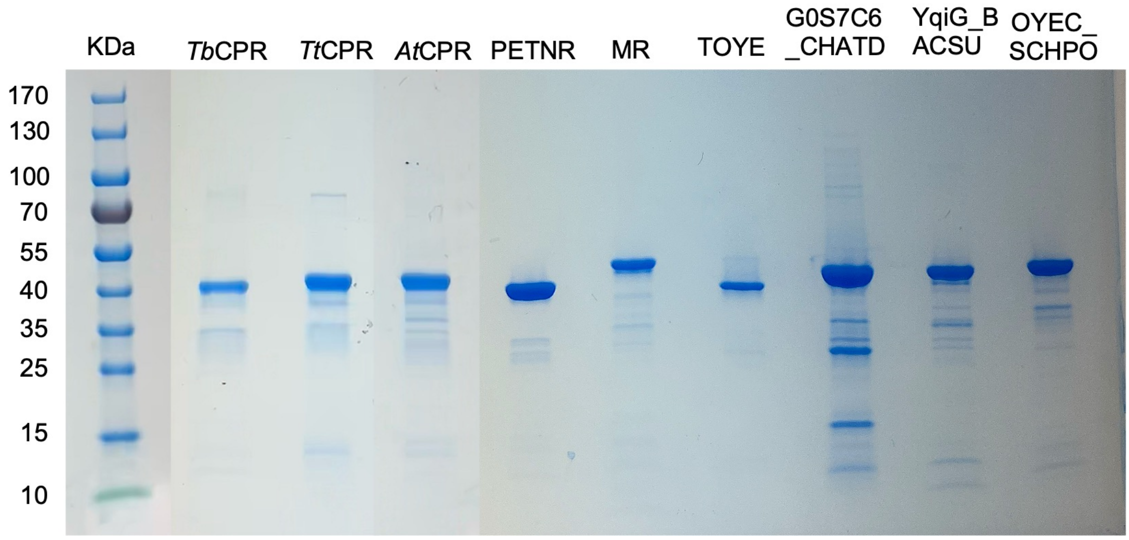

Fig. S4: SDS-PAGE gel of purified enzymes used in this study.

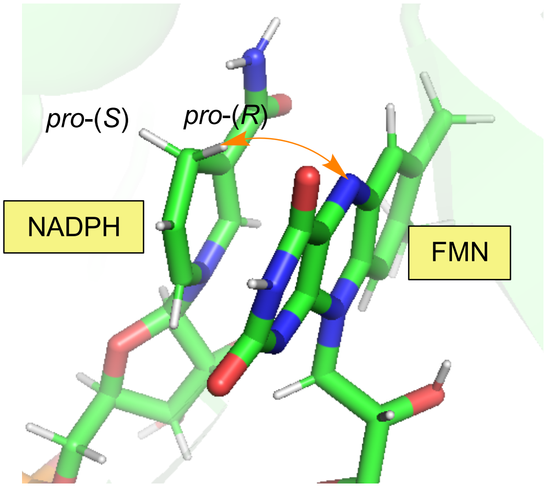

Fig. S5: Molecular docking of NADPH in the active site of *At*CPR. Transfer of the *pro*-(*R*) hydrogen of NADPH to FMN is reversible.

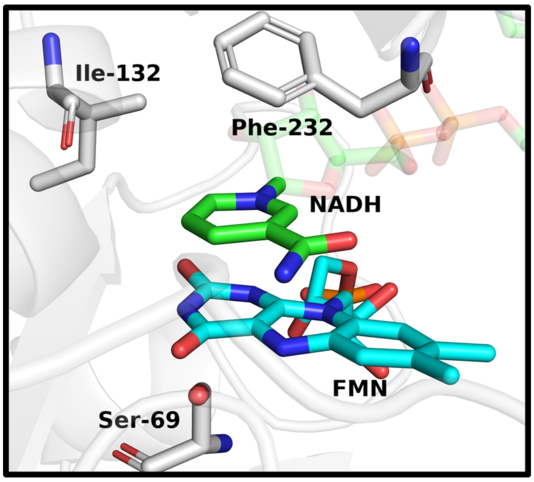

Fig. S6: MD simulation of NADH binding pose consistent with *pro*-(*R*) hydride transfer within the active site of *At*CPR. This representative structure was chosen based on a clustering analysis of the Cα (protein) RMSD.

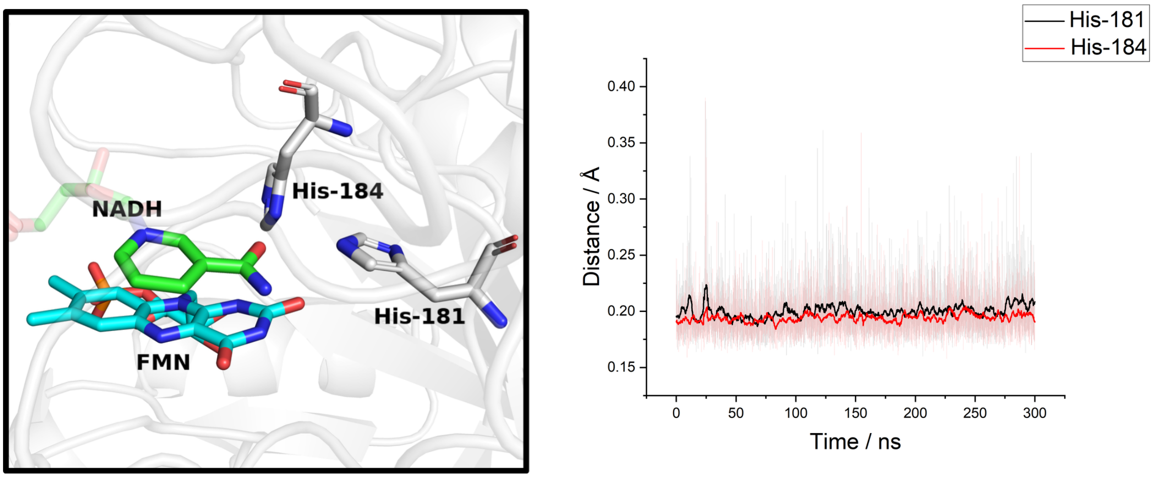

Fig. S7: Interactions between PETNR and the amide group of NADH. **Left:** NADH bound in the active site of PETNR. A representative structure was chosen based on a clustering analysis of the Cα (protein) RMSD. **Right:** Distances between the amide oxygen and the two histidine residues during the production simulation of PETNR.

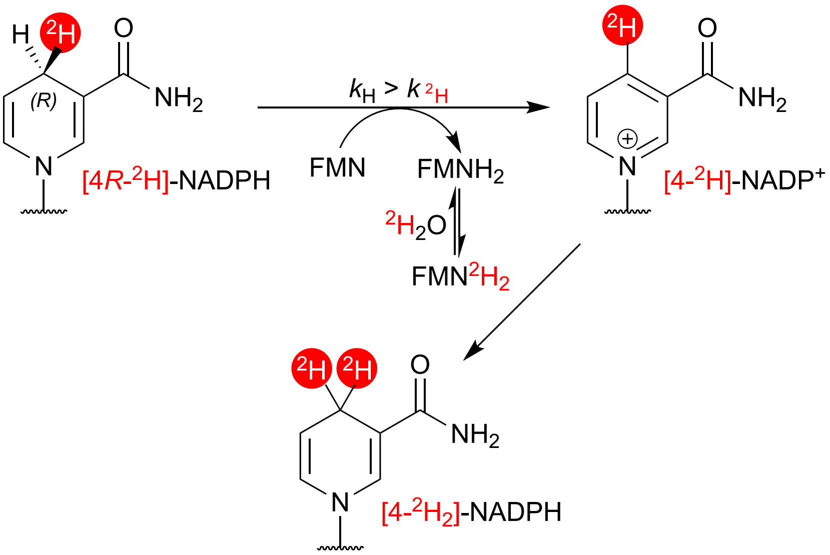

Fig. S8: Speculative mechanism depicting the formation of [4-$^{2}H_2$]-NADPH in the *At*CPR- and *Tt*CPR-catalysed HIE reactions.

S3. Methods and materials

S3.1. General information

All reactions were set up and analytical samples prepared in a glovebox under a protective N_2 atmosphere (O_2 < 0.1 ppm). Unless stated otherwise, general reagents and NAD(P)H (Sigma Aldrich, US), buffer salts and $NAD(P)^+$ (Prozomix, UK) were all used as received, without further purification. All non-deuterated solutions were prepared with MilliQ water (Millipore, 18 MΩcm) and deuterated solutions with ^2H_2O (>99.9%, Apollo Scientific, UK). All buffers and solutions were deoxygenated by sparging with dry N_2 for a minimum of 30 minutes prior to use.

S3.1.1. Preparation of deuterated buffers

($^2H_5$)-Tris-^{2}HCl was prepared by dissolving Trizma® base (Formedium, UK) in ^2H_2O followed by evaporation under reduced pressure. After two more repetitions of redissolving and reevaporating, the p^{2}H was set to 8.4 by the addition of ^{2}HCl. The buffer solution was then deoxygenated by sparging with dry N_2 for a minimum of 30 minutes.

S3.1.2. Analytical instrumentation

^{1}H NMR

For the initial screen/time-course experiments, ^{1}H NMR was measured at 310 K using a Bruker Avance III 800 MHz spectrometer equipped with a 5 mm TCI cryoprobe and SampleJet autosampler. For the 24-hour reactions, ^{1}H NMR was measured at room temperature (298 K) using a Bruker Neo nanobay 400 MHz spectrometer with BBO probe. ^2H_2O was used as the NMR solvent except for the control experiments in ^1H_2O, where the NMR solvent was 12.5% ^2H_2O in ^1H_2O. Chemical shifts (δ) are given in ppm, referenced to ($^2H_6$)-sodium trimethylsilylpropanesulfonate (DSS). All NMR experiments used solvent suppression using the Bruker noesygppr1d pulse program and carried out with 128 scans, except for the control experiments in ^1H_2O, which were performed with 16 scans.

UV-vis

Ultraviolet-visible spectroscopy (UV-vis) measurements were made in a quartz cuvette (path length = 1 cm) on a Cary 3500 UV-vis spectrophotometer (Agilent, US).

UPLC-MS

UPLC-MS was conducted with a 1290 Infinity II LC system (Agilent, US) coupled with an InfinityLab LC/MSD XT single-quadrupole Mass Selective Detector and UV-spectrophotometric detection (MSD, Agilent).

S3.2. Enzymes

S3.2.1. Commercially available enzymes

Glutathione reductase (GR) from *Saccharomyces cerevisiae* was purchased from Sigma Aldrich as an ammonium sulfate suspension. GR was used as received, without further purification. NADH diaphorase from *Clostridium kluyveri* was purchased from Sigma Aldrich as a lyophilised powder and was used as received, without further purification.

S3.2.2. Enzyme expression and purification

CYP116B reductases

A heat-shock protocol provided by New England Biolabs was used to transform pET-28a-(+) containing a codon optimised gene sequence for the reductase domains of CYP116B (CPRs) into chemically competent *E. coli* BL21 (DE3) cells. Cells were plated on Lysogeny Broth (LB) agar containing kanamycin (50 μg/mL). A single colony was picked and used to inoculate 5 mL of LB medium (Formedium, UK) containing kanamycin (50 μg/mL). Cells were incubated at 37 °C and shaken at 200 rpm overnight (overnight culture). Final cultures in 2 L Erlenmeyer baffled flasks containing 800 mL autoinduction Terrific Broth (TB) media (Formedium, UK) supplemented with 50 μg/mL kanamycin were inoculated with 5 mL of cells from the overnight culture. Cultures were incubated at 23 °C and shaken at 200 rpm for 60 hours before harvesting the biomass using centrifugation at 4,000 rpm, 4 °C for 30 minutes. Cell pellets were resuspended in 30 mL lysis buffer (100 mM KPi (pH 8.0), 300 mM NaCl, 20 mM imidazole, 10% glycerol). Cells were disrupted by sonication, and the lysate was clarified by centrifugation at 18,000 rpm, 4 °C for 30 minutes. The supernatant was combined with Ni-NTA agarose resin and incubated at 4 °C in an orbital shaker (130 rpm) for 1 hour. The mixture was loaded onto a gravity flow column, and the resin was washed with lysis buffer. The protein was eluted with elution buffer (100 mM KPi (pH 8.0), 300 mM NaCl, 100 mM imidazole, 10% glycerol) and buffer exchanged into storage buffer (100 mM KPi (pH 8.0), 300 mM NaCl, 30% glycerol) using PD-10 desalting columns packed with Sephadex G-25 resin (Cytiva, US). Unless otherwise stated, protein concentration was calculated using the flavin extinction coefficient determination method of Chapman and Reid.[1] Enzymes were either used immediately or stored at -80 °C after snap freezing.

PETNR, MR, TOYE, YqiG_BACSU, G0S7C6_CHATD and OYEC_SCHPO

The procedure for the purification of pentaerythritol tetranitrate reductase (PETNR) was adapted from previous reports,[2] and used for morphinone reductase (MR) and old yellow enzymes TOYE, YqiG_BACSU, G0S7C6_CHATD and OYEC_SCHPO. Codon-optimised DNA sequences for PETNR, MR, TOYE, YqiG_BACSU, G0S7C6_CHATD and OYEC_SCHPO were synthesised (Geneart, Life Technologies) and cloned into a pET21a-(+) plasmid. *E.coli* BL21 (DE3) were transformed and plated on LB agar containing ampicillin (0.1 mg/mL). Transformed colonies were grown overnight in LB broth (5 mL, +0.1 mg/mL ampicillin) and used to inoculate 500 mL LB in 2 L flasks. Cells were grown at 37 °C with shaking (180 rpm) until they reached 0.6 OD_{600}. Protein expression was induced with 0.5 mM isopropyl β-d-1-thiogalactopyranoside (IPTG). The temperature was lowered to 25 °C and cultures were incubated for 16-18 hours. Cells were harvested by centrifugation at 9000 x g for 20 minutes at 4 °C. Cell pellets were collected and stored at -20 °C. Cell pellets were resuspended in buffer A (50 mM KH_2PO_4/K_2HPO_4 pH 7.0, 300 mM NaCl and 10 mM imidazole, 0.25 mg ml^{-1} lysozyme and 10 µg ml^{-1} DNase) and lysed by sonication (amplitude 35%, 30 cycles of 10 seconds on/ 10 seconds off). Protease inhibitor cocktail tablets were added to prevent proteolysis. The lysate was clarified by centrifugation at 30000 x g for 30 minutes at 4 °C. The supernatant was incubated with Ni-NTA agarose resin (pre-equilibrated with buffer A) for 1 hour at 4 °C with gentle mixing. The resin was transferred to a gravity flow column and washed with buffer B (50 mM KH_2PO_4/K_2HPO_4 pH 7.0, 300 mM NaCl and 20 mM imidazole) to remove non-specifically bound proteins. Target proteins were eluted with Buffer C (50 mM KH_2PO_4/K_2HPO_4 pH 7.0, 300 mM NaCl and 250 mM imidazole). Desalting was performed by gel filtration using a Zetadex-50 (EMP Biotech) using a storage buffer (50 mM KH_2PO_4/K_2HPO_4 pH 7.0, 300 mM NaCl). Purified proteins were frozen in liquid nitrogen and stored at -80 °C.

S3.2.3. Digestion of *At*CPR for the removal of [2Fe-2S] cluster

Table S2: Primers used for the digestion of *At*CPR for the removal of the [2Fe-2S] cluster.

Primer name	Primer sequence	Melting temperature (Tm)	GC%
*At*CPR-FMN-F	GGGAATTCCATATGGTGAGCGTCGTGTCGGCCG	67	61
*At*CPR-FMN-R	CCCAAGCTTTAGGGTGAAGTGCTCGACGTGCAGC	65	59

Genes were amplified using *Phusion* polymerase according to the manufacturer's protocol with 30 seconds of elongation time. The amplified genes were digested with *NdeI* and *HindIII*, and the digested gene was cloned into pET28a(+) plasmid. *At*CPR-FMN was expressed and purified using the aforementioned method for the CYP116B reductases.

S3.2.4. Calculation of enzyme concentration

Flavoenzyme concentrations were calculated using the Beer-Lambert law. Unknown flavin extinction coefficients were determined using an adapted method by Chapman and Reid.[1] ~10 mg/mL of flavoprotein was diluted in 10 mM Tris-HCl, pH 8.0 to give a final volume of 900 μL. Samples were transferred to cuvettes and the absorbance at 446 nm was recorded using a UV-vis spectrophotometer (measurement A). 100 μL of 50% trichloroacetic acid was added to each sample, followed by centrifugation at 13.3 k rpm for 2 minutes. Samples were neutralised with the addition of 1 M $NaCO_3$ (200 μL) and the absorbance of the supernatant was measured at 446 nm. The obtained absorbances were corrected for the dilution factor (measurement B) and extinction coefficients calculated using the following:

$$\varepsilon_{\text{unknown}} = 12{,}200 \times \text{measurement A}/\text{measurement B}$$

S3.2.5. Protein sequences

*At*CPR – MW: 35.7 kDa

MGSSHHHHHHSSGLVPRGSHMASMTGGQQMGRGSSIAELRRPVRIGEPSKRGVARTVTV
VSVERAAEDVVEVTLAATDGKPLPKWSPGAHVDLQLGELSRQYSLCGDPAEAGTYRIAVLK
DPDSRGGSRYMHENLRPGTTLSLRGPRNHFRLDPTARHYVLVAGGIGITPIIAMADHVKAVG
GSYELHYCGRDTTAMALLDRCRRDHGDRLHVHTSADGTRLDIAALLANPAEGTQIYACGPE
RLLDALAEASAHWPDDALHVEHFTTTQQVLDPAVEHAFDVDLADSGLTVRVAADQTVLAAL
RAAGVDVPSDCEEGLCGTCEVPVLDGEVDHRDVVLTKAERAAGTRMMTCCSRACGDRITL
RL

*Tb*CPR – MW: 36.2 kDa

MGSSHHHHHHSSGLVPRGSHMASMTGGQQMGRGSEILRRRLPVQIGSARRPLPRSVTVL
AIEPEAEGIVRITLADAHGKPLPAWSPGAHVDLELGGLTRQYSLCGDPDDRSTYQIAVLKDP
DSRGGSRYVHERLAVGDVLLMRGPRNNFPLDPGARRYVFIAGGIGITPIITMADRARRRGAD
YHIHYCGRSAAAMAFLGRLRRDHGDRLTLYRSAEGTRLDIARLLAEPQEGTQIYVCGPQRM
LDAVAEATRHWPDDAVRVESFVSALDRFDPAKNHAFDVRLADSGRVIRVPADQTVLGALRA
AGIDVLSDCEEGLCGTCEVPVAEGEVDHRDMVLTKAERAQHSSMMTCCSRARGGSITLRL

*Tt*CPR – MW: 38.1 kDa

MGSSHHHHHHSSGLVPRGSHMASMTGGQQMGRGSDILRLRQPVRIGPPRAKDVVRTMEV
AAVERPSEDIVVLHLTRPDRRPLPRWSPGAHIDIECGEPDRSRQYSLCSDPENRDAWRVAV
QRDPASRGGSRWIHEEVRPGMLLRVRGPRNSFRLDEHAPRYLFLAGGIGITPIMTMAARAK
ELGTDYELHYSVRSRTSLIFVDELRQIHGDRLHVYVSEEGVRNDLAALIRRASAGTQIYACG

PQRMLDTLERLIENRPEVTLRVEHFFGEPSHLDPAKERPFQVVLRNSGLTVEVPADKTLLEV
LRAYNIEVQSDCEEGLCGTCEVSVVEGEVDHRDSVLTRAERRENRRMMCCCSRAKTERLV
LDL

PETNR – MW: 39.5 kDa

MSAEKLFTPLKVGAVTAPNRVFMAPLTRLRSIEPGDIPTPLMGEYYRQRASAGLIISEATQISA
QAKGYAGAPGLHSPEQIAAWKKITAGVHAEDGRIAVQLWHTGRISHSSIQPGGQAPVSASAL
NANTRTSLRDENGNAIRVDTTTPRALELDEIPGIVNDFRQAVANAREAGFDLVELHSAHGYL
LHQFLSPSSNQRTDQYGGSVENRARLVLEVVDAVCNEWSADRIGIRVSPIGTFQNVDNGPN
EEADALYLIEELAKRGIAYLHMSETDLAGGKPYSEAFRQKVRERFHGVIIGAGAYTAEKAEDLI
GKGLIDAVAFGRDYIANPDLVARLQKKAELNPQRPESFYGGGAEGYTDYPSL

MR – MW: 41.3 kDa

MPDTSFSNPGLFTPLQLGSLSLPNRVIMAPLTRSRTPDSVPGRLQQIYYGQRASAGLIISEAT
NISPTARGYVYTPGIWTDAQEAGWKGVVEAVHAKGGRIALQLWHVGRVSHELVQPDGQQP
VAPSALKAEGAECFVEFEDGTAGLHPTSTPRALETDEIPGIVEDYRQAAQRAKRAGFDMVE
VHAANACLPNQFLATGTNRRTDQYGGSIENRARFPLEVVDAVAEVFGPERVGIRLTPFLELF
GLTDDEPEAMAFYLAGELDRRGLAYLHFNEPDWIGGDITYPEGFREQMRQRFKGGLIYCGN
YDAGRAQARLDDNTADAVAFGRPFIANPDLPERFRLGAALNEPDPSTFYGGAEVGYTDYPF
LDNGHDRLG

OYEC_SCHPO – MW: 43.7 kDa

MTIVNEGAENVGYFTPAQKIPAGAAIGVPQTKLFTPLKIRGVEFHNRMFVSPMCTYSADQEG
HLTDFHLVHLGAMGMRGPGLVMVEATAVSPEGRISPNDSGLWMESQMKPLRRIVEFAHSQ
NQKIGIQLAHAGRKASTTAPYRGYTVATEAQGGWENDVYGPNEDRWDENHAQPHKLTEKQ
YDELVDKFVVAAKRAVEIGFDVIEIHGAHGYLISSTVSPATNDRNDKYGGTFEKRILFPMEVV
HSVRKAIPDSMPLFYRVTATDWLPKGQGWEIEDTVALAARLRDGGVDLIDVSSGGNHKDQR
IEVKDCYQVPFAEKIKDQVNGILLGAVGMIRDGLTANEILESGKADVTFVAREFLRNPSLVLDS
ANQLGENVAWPVQYDYAVKGHRKLR

TOYE – MW: 38.2 kDa

MSILHMPLKIKDITIKNRIMMSPMCMYSASTDGMPNDWHIVHYATRAIGGVGLIMQEATAVES
RGRITDHDLGIWNDEQVKELKKIVDICKANGAVMGIQLAHAGRKCNISYEDVVGPSPIKAGD
RYKLPRELSVEEIKSIVKAFGEAAKRANLAGYDVVEIHAAHGYLIHEFLSPLSNKRKDEYGNS
IENRARFLIEVIDEVRKNWPENKPIFVRVSADDYMEGGINIDMMVEYINMIKDKVDLIDVSSG

GLLNVDINLYPGYQVKYAETIKKRCNIKTSAVGLITTQELAEEILSNERADLVALGRELLRNPY
WVLHTYTSKEDWPKQYERAFKK

YqiG_BACSU – MW: 40.8 kDa

MNPKYKPLFEPFTFKSGVTINNRIAVAPMTHYASNEDGTISEAELDYIIPRSKEMGMVITACAN
VTPDGKAFPGQPAIHDDSNIPGLKKLAQAIQAQGAKAVVQIHHGGIECPSELVPQQDVVGPS
DVFDNGKQIARALTEEEVENIVKAFGEATRRAIEAGFDGVEIHGANGYLIQQFYSPKTNQRT
DRWGGSDEKRLAFPLAIVDEVKKAASEHAKGAFLVGYRLSPEEPETPGLTMTETYTLVDAL
GDKELDYLHISLMDVNSKARRGADPTRTRMDLLNERVGNKVPLIAVGSIHSADDALAVIENGI
PLVAMGREILVDPNWTVKVKEGREKQIETVIKGTDKEKYHLPEPLWQAIVNTQGWVPYKD

G0S7C6_CHATD – MW: 41.2 kDa

MSPSNNTRLFEPLQLGTVTLSHRIAMAPLTRFRALDSHVPQLPLVAEYYTQRASIPGTLLITE
GTFIAQHAGGLPNIPGIWNEDQIKAWKVVTDAVHAKGSFIFCQLWALGRAANPKVAEAEGFK
VKSSSAVPIEEGGVVPEEMTVEEIKEMVKAYANAARNAIKAGFDGVEIHGANGYLVDQFIQD
KCNQRTDEYGGSVENRSKFAVEVVKAVAGAVGPEKTAIRLSPWSRFQGMKMDDPRPQFLD
VIRKISGLGLAYLHLVRSGVGGPNDFNQAGEDETLDFAVDLWDGPVLIAGKLTPETARDLVD
HQYKDKKVVATFGKYFISNPDLPFRIKEGIPLNPYDRSTFYTPKSPVGYTDQPFSKEFQESQ
TSTL

*At*CPR-FMN

MGSSHHHHHHSSGLVPRGSHVSVVSAERAAEDVVEVTLAATDGKPLPKWSPGAHVDLQL
GELSRQYSLCGDPAEACTYRIAVLKDPDSRGGSRYVHENLRPGTTLSLRGPRNHFRLDPTA
RHYVFVAGGIGITPIIAMADHVKAAGGSYELHYCGRDTTAMALLDRCRRDHGDRLHVHTSA
DGTRLDVAALLANPAEGTQIYACGPERLLDALAEASAHWPDDALHVEHFT

S3.3. Biocatalysis

Prior to all experiments, enzymes were exchanged from $^{1}H_2O$ storage buffer into $^{2}H_2O$ by diluting the desired volume of enzyme in 20 mL $^{2}H_2O$, followed by centrifugation in a protein concentrator with a 10 kDa molecular weight cutoff filter (Cytiva, US).

S3.3.1. General procedure for the ^{1}H NMR (800 MHz) time-course

600 μL reaction mixtures containing 5 mM NAD(P)H, 30 μM flavoenzyme, 0.5 mM DSS internal standard and 10 mM ($^{2}H_5$)-Tris-^{2}HCl in $^{2}H_2O$, p^{2}H 8.4 were incubated in high-throughput NMR

sample tube racks at 37 °C in the SampleJet autosampler. NMR spectra were measured at 37 °C. An initial measurement was made 30 minutes after the addition of enzyme. Subsequent measurements were made in parallel for 24 reactions, every 4 hours for a maximum reaction time of 20 hours. Data was processed and analysed with MestReNova.

S3.3.2. General procedure for the 24-hour HIE reactions

A 750 μL reaction mixture containing 5 mM NAD(P)H, 30 μM flavoenzyme and 10 mM ($^2H_5$)-Tris-^{2}HCl in ^2H_2O, p^{2}H 8.4 was stirred in an aluminium foil covered 4 mL glass vial at 37 °C for 24 hours. Following clarification of the reaction mixture by centrifugation, analysis by UPLC-MS, ^{1}H NMR and UV-vis spectroscopy was performed.

S3.3.3. Sample preparation and procedure for UPLC-MS analysis

Enzymes were removed from reaction mixtures by centrifugation at 9,800 x g for 5 minutes through 500 μL Vivaspin® centrifugal concentrators with 10 kDa molecular weight cutoff filters (Sartorius, Germany). Filtrates were collected, diluted with MilliQ water to a final cofactor concentration of 1 mg/mL, and added to HPLC filter vials (Thomson, 0.45 μm). Unless otherwise stated, UPLC-MS separations were performed according to the following method:

Separation mode: hydrophilic interaction liquid chromatography (HILIC)
Column: Waters Acquity UPLC Glycan BEH Amide Column (130 Å, 1.7 μm, 2.1 mm x 150 mm) equipped with a Waters Acquity UPLC Glycan BEH Amide VanGuard Pre-column (130 Å, 1.7 μm, 2.1 mm x 5 mm)
Buffer A: 90% MeCN: 10% HPLC water, 20 mM ammonium acetate, pH 7.5
Buffer B: 100% HPLC water, 20 mM ammonium acetate, pH 7.5
Column temperature: 40 °C
Flow rate: 0.3 mL/min
Injection volume: 2 μL
UV detection: 260, 340, 345 and 395 nm
Mass detection: Electrospray ionisation (ESI) in negative ion mode
Mobile phase profile: 0 → 1 min: 100% A, 0% B (isocratic); 1 → 15 mins: 100% A, 0% B to 30% A, 70% B (linear gradient); 15 → 17 mins: 30% A, 70% B (isocratic); 17 → 18 mins: 30% A, 70% B to 100% A, 0% B (re-equilibration); 18 → 19 mins: 100% A, 0% B (isocratic).

S3.3.4. Sample preparation for UV-vis spectrophotometric analysis

20 µL of clarified reaction mixture was diluted in 10 mM Tris, pH 8.0 (980 µL) for a final cofactor concentration of 0.1 mM. This solution was then added to a quartz cuvette with a path length of 1 cm. A background spectrum of 10 mM Tris, pH 8.0 was subtracted from the sample spectrum.

S3.3.5. Determination of cofactor proportions by ^{1}H NMR spectroscopy

30 mM DSS in $^{2}H_2O$ (100 µL) was added to 700 µL of clarified reaction mixture. 600 µL of this was added to a standard NMR sample tube and sealed with parafilm. The ^{1}H NMR spectra of the nicotinamide cofactors and their deuterated analogues was assigned according to established reports.[3] The proportions of NAD(P)H : NAD(P)$^{+}$: [4*R*-$^{2}H_2$]-NAD(P)H : [4*S*-$^{2}H_2$]-NAD(P)H : [4-$^{2}H_2$]-NAD(P)H shown in table 1 were calculated as follows: Firstly, the proportion of NAD(P)$^{+}$ in the reaction mixture was determined by calculating the percentage increase of the integral of the singlet at 9.32 ppm (for NADP^{+}) or 9.38 ppm (for NAD^{+}) compared to commercial standards of NAD(P)$^{+}$. Secondly, the proportion of dideuteration product ([4-$^{2}H_2$]-NAD(P)H) in the reaction mixture was determined by calculating the percentage decrease of the integral of the multiplet between ~2.69 – ~2.88 ppm (corresponding to H4 of the dihydronicotinamide ring of NAD(P)H, fig. S9) relative to the integral of the singlet corresponding to H2 of the dihydronicotinamide ring (6.93 ppm for NADPH, 6.94 ppm for NADH). Thirdly, the sum of the percentages calculated for NAD(P)$^{+}$ and [4-$^{2}H_2$]-NAD(P)H were deducted from 100% and the multiplet between ~2.69 – ~2.88 ppm was used to determine the remaining relative proportions of [4*R*-^{2}H]-NAD(P)H and [4*S*-^{2}H]-NAD(P)H by integrating each signal and using the equations shown in fig. S9.[4] Finally, the remaining percentage was attributed to unreacted NAD(P)H.

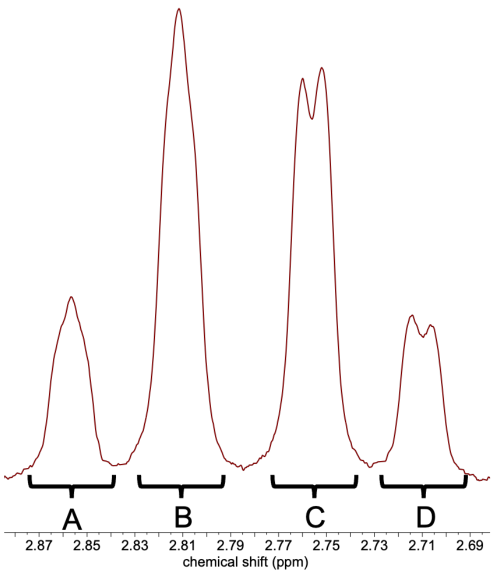

$$\% \ [4R\text{-}^2\text{H}]\text{-NAD(P)H} = \frac{(\text{D} - \text{A})}{(\text{B} + \text{D})} \times 100$$

$$\% \ [4S\text{-}^2\text{H}]\text{-NAD(P)H} = \frac{(\text{B} - \text{C})}{(\text{B} + \text{D})} \times 100$$

Fig. S9: Characteristic ^{1}H NMR multiplet for the diagnostic region corresponding to the redox active protons of NAD(P)H and equations used to calculate the relative proportions of [4*R*-^{2}H]- and [4*S*-^{2}H]-NAD(P)H.

The equations in S9 are derived from the following rationale, from consideration of the peak various splittings and coincident peaks:

For 1,4-NAD(P)H

$H_{Pro\text{-}(R)}$ gives rise to a minor peak (**A**) and a major peak (**B**)
$H_{Pro\text{-}(S)}$ gives rise to a minor peak (**D**) and a major peak (**C**)

Integrating 2 minor and 2 major peaks together gives the area for 2 protons
Integrating 1 minor and 1 major peak together gives the area for 1 proton

For [4*S*-^{2}H]-NAD(P)H

$H_{(R)}$ gives rise to a single peak (**B**), which integrates to 1 proton

For [4*R*-^{2}H]-NAD(P)H

$H_{(S)}$ gives rise to a single peak (**D**), which integrates to 1 proton

For a mixture of 1,4-NAD(P)H, [4*S*-^{2}H]-NAD(P)H, and [4*R*-^{2}H]-NAD(P)H

Peak **A** represents 1,4-NAD(P)H_{minor}
Peak **B** represents convoluted [4*S*-^{2}H]-NAD(P)H and 1,4-NAD(P)H_{major}
Peak **C** represents 1,4-NAD(P)H_{major}
Peak **D** represents convoluted [4*R*-^{2}H]-NAD(P)H and 1,4-NAD(P)H_{minor}

Hence:

B + **D** = [4*S*-^{2}H]-NAD(P)H + [4*R*-^{2}H]-NAD(P)H + [1,4-$NADPH_{major}$ + 1,4-$NADPH_{minor}$]
= [4*S*-^{2}H]-NAD(P)H + [4*R*-^{2}H]-NAD(P)H + [Area from 1 proton of 1,4-NADPH]
D – **A** = ([4*R*-^{2}H]-NAD(P)H + 1,4-$NADPH_{minor}$) – 1,4-$NADPH_{minor}$ = [4*R*-^{2}H]-NAD(P)H
B – **C** = ([4*S*-^{2}H]-NAD(P)H + 1,4-$NADPH_{major}$) – 1,4-$NADPH_{major}$ = [4*S*-^{2}H]-NAD(P)H

S3.3.6. Preparative-scale synthesis of [4*S*-^{2}H]-NADPH

NADPH tetrasodium salt (25 mg, 0.03 mmol) was added to an aluminium foil covered 4 mL glass vial containing a magnetic stirrer. 10 mM ($^2H_5$)-Tris-^{2}HCl in ^2H_2O, p^{2}H 8.4 and 34 U glutathione reductase (GR) was added for a final reaction volume of 2 mL. The reaction mixture was stirred at 37 °C for 24 hours and subsequently quenched by the addition of acetonitrile (1 volume). Precipitated biomolecules were pelleted at 6,000 rpm for 10 minutes. Purification was performed using a modified method by Basran and co-authors.[5] Briefly, the crude reaction mixture was loaded onto a Q-Sepharose column (Cytiva, US) which was pre-equilibrated with 10 mM $(NH_4)HCO_3$, pH 9.0. The column was run with 50 mM increases of $(NH_4)HCO_3$ from 10 mM $(NH_4)HCO_3$ to 510 mM $(NH_4)HCO_3$, pH 9.0 (step gradient). The A_{260}/A_{340} ratio of cofactor-containing fractions was determined by UV-vis spectrophotometry, and fractions with a ratio of ≤2.5 were combined and freeze-dried.

S3.3.7. Preparative-scale synthesis of [4*R*-^{2}H]-NADPH

NADPH tetrasodium salt (25 mg, 0.03 mmol) was added to an aluminium foil covered 4 mL glass vial containing a magnetic stirrer. 10 mM ($^2H_5$)-Tris-^{2}HCl in ^2H_2O, p^{2}H 8.4 and 90 μM pentaerythritol tetranitrate reductase (PETNR) was added for a final reaction volume of 2 mL. The reaction mixture was stirred at 37 °C for 24 hours and subsequently quenched by the addition of acetonitrile (1 volume). Precipitated biomolecules were pelleted at 6,000 rpm for 10 minutes. Purification was performed using a modified method by Basran and co-authors.[5] Briefly, the crude reaction mixture was loaded onto a Q-Sepharose column (Cytiva, US) equilibrated with 10 mM $(NH_4)HCO_3$, pH 9.0. The column was run with 50 mM increases of $(NH_4)HCO_3$ from 10 mM $(NH_4)HCO_3$ to 510 mM $(NH_4)HCO_3$, pH 9.0 (step gradient). The A_{260}/A_{340} ratio of cofactor-containing fractions was determined by UV-vis spectrophotometry, and fractions with a ratio of ≤2.5 were combined and freeze-dried.

S3.3.8. Preparative-scale synthesis of [4-$^2H_2$]-NADPH

NADPH tetrasodium salt (25 mg, 0.03 mmol) was added to an aluminium foil covered 4 mL glass vial containing a magnetic stirrer. 10 mM ($^2H_5$)-Tris-^{2}HCl in ^2H_2O, p^{2}H 8.4, 90 μM PETNR and 34 U glutathione reductase was added for a final reaction volume of 2 mL. The reaction was stirred at 37 °C for 24 hours and subsequently quenched by the addition of acetonitrile (1 volume). Precipitated biomolecules were pelleted at 6,000 rpm for 10 minutes. Purification was performed using a modified method by Basran and co-authors.[5] Briefly, the crude reaction mixture was loaded onto a Q-Sepharose column (Cytiva, US) equilibrated with 10 mM $(NH_4)HCO_3$, pH 9.0. The column was run with 50 mM increases of $(NH_4)HCO_3$ from 10 mM

$(NH_4)HCO_3$ to 510 mM $(NH_4)HCO_3$, pH 9.0 (step gradient). The A_{260}/A_{340} ratio of cofactor-containing fractions was determined by UV-vis spectrophotometry, and fractions with a ratio of ≤2.5 were combined and freeze-dried.

S3.4. Molecular modelling

S3.4.1. Model setup

Structures of the ternary complexes of *At*CPR and PETNR were predicted using AlphaFold 3, with FAD and NADH included as ligands.[6] In this version of AlphaFold, FMN was not available as a ligand option. To assess whether the predicted FAD binding pose was reasonable, *At*CPR was aligned with a reported homologue (PDB ID: 6LAA)[7] and PETNR was aligned with a reported crystal structure (PDB ID: 3KFT).[8] Both reference structures contain an FMN cofactor, and the alignments revealed similar binding poses for the FAD cofactor. In both predicted structures, the FAD adenosine extends outside the protein and was not involved in the model (Fig. S10).

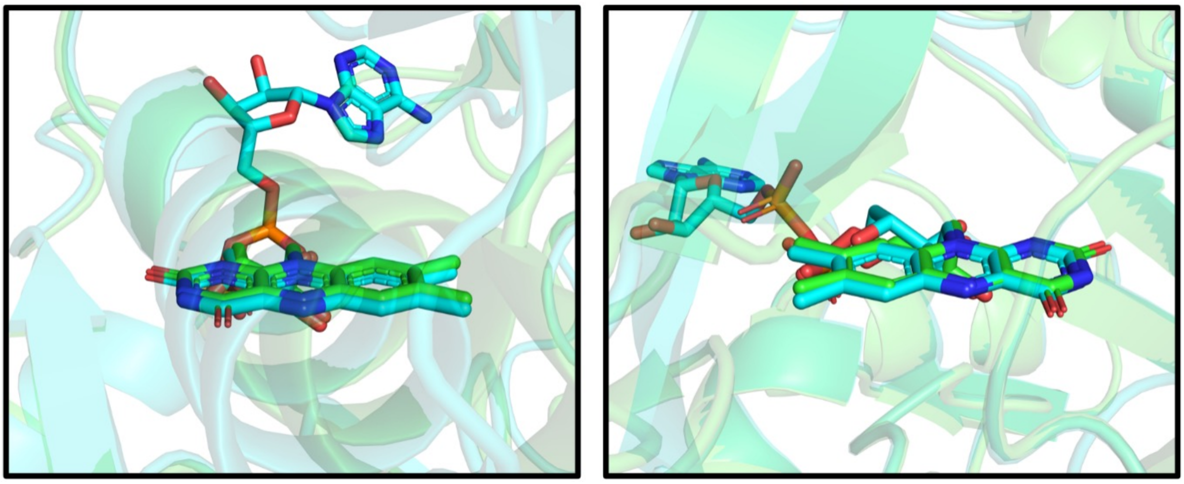

Fig. S10: Structural alignments between reported structures (green) and AlphaFold predicted structures (blue) for *At*CPR and PETNR. **Left:** *At*CPR alignment with an overall RMSD of 0.264 Å. **Right:** PETNR alignment with an overall RMSD of 0.236 Å.

In the AlphaFold model of *At*CPR, the nicotinamide ring of NADH was positioned outside the active site, while the side chain of Phe-232 formed a π-stacking interaction with the isoalloxazine ring of the FAD cofactor. The FAD cofactors were truncated to FMN with the phosphate group fully deprotonated (overall charge of -2). For *At*CPR, the [2Fe-2S] cluster was inserted by aligning the AlphaFold model with a reported homologue (PDB ID: 6LAA).[7] Protonation states were assigned using the H++ web server at pH 7.0, and cysteine residues coordinating the [2Fe-2S] cluster were manually deprotonated.[9] Each system was solvated in a cubic box of TIP3P water molecules extending 10 Å beyond the protein surface, with counterions added to neutralise the total charge.[10]

Merz-Kollman restrained electrostatic potential (RESP) charges were generated in Gaussian09.[11] For the [2Fe-2S] cluster and FMN + NADH, calculations were performed at the B3LYP/6-31G(d) and HF/6-31G(d) level of theory, respectively.[12] Force field parameters for the [2Fe-2S] cluster were taken from Carvalho *et al*. and RESP charge fitting was performed using MCPB.py (part of AmberTools).[13] For FMN and NADH, RESP charge fitting was performed in Antechamber (part of AmberTools) and force field parameters were assigned using GAFF2 atom types. The Amber ff14SB force field was applied to every protein atom.[14] Topology files were converted between Amber and GROMACS using ParmEd.[15]

S3.4.2. System equilibration and molecular dynamics

All energy minimisations, equilibrations, and production simulations were carried out in GROMACS 2021.5.[16] Energy minimisation was followed by multi-step equilibration under NVT and NPT ensembles, with gradual release of position restraints (1000 → 500 → 250 → 100 kJ mol^{-1} nm^{-2} in 500 ps increments). Temperature coupling was achieved using the V-rescale thermostat, and pressure coupling was applied independently in all directions using the Berendsen barostat.[17]

After equilibrating *At*CPR, pulling (steered MD) was performed between the nicotinamide ring of NADH and the isoalloxazine ring of FMN. To accommodate for this, a flat-bottom-high distance restraint was first applied between the side chain of Phe-232 and the isoalloxazine ring. An umbrella potential was then applied between the nicotinamide and isoalloxazine rings at a pulling rate of -0.05 nm ns^{-1}.[18] During the simulations, temperature coupling was achieved using the Nose-Hoover thermostat and pressure coupling was maintained using the Parrinello-Rahman barostat. Configurations in which the nicotinamide ring was positioned within the active site of *At*CPR were extracted, equilibrated with new initial velocities, and used for 1 μs production simulations employing the same coupling schemes. Production simulations were conducted from two different NADH binding poses that were consistent with either *pro-R* or *pro-S* hydride transfer. A 300 ns production simulation of PETNR was performed using identical temperature and pressure coupling conditions. To ensure that the coenzyme stayed in the active site of *At*CPR, a flat-bottom distance restraint was applied between the nicotinamide ring and the isoalloxazine ring during the production simulation.

All analyses were conducted using tools within the GROMACS package. Representative structures from each production simulation were selected via clustering analysis of the Cα (protein) RMSDs (gromos clustering algorithm).[19] For *At*CPR and PETNR, RMSD cutoff values of 0.20 and 0.15 nm were used, respectively. Large motions were

observed in the *N*-terminal linker region of *At*CPR and in the NADH adenine in both *At*CPR and PETNR.

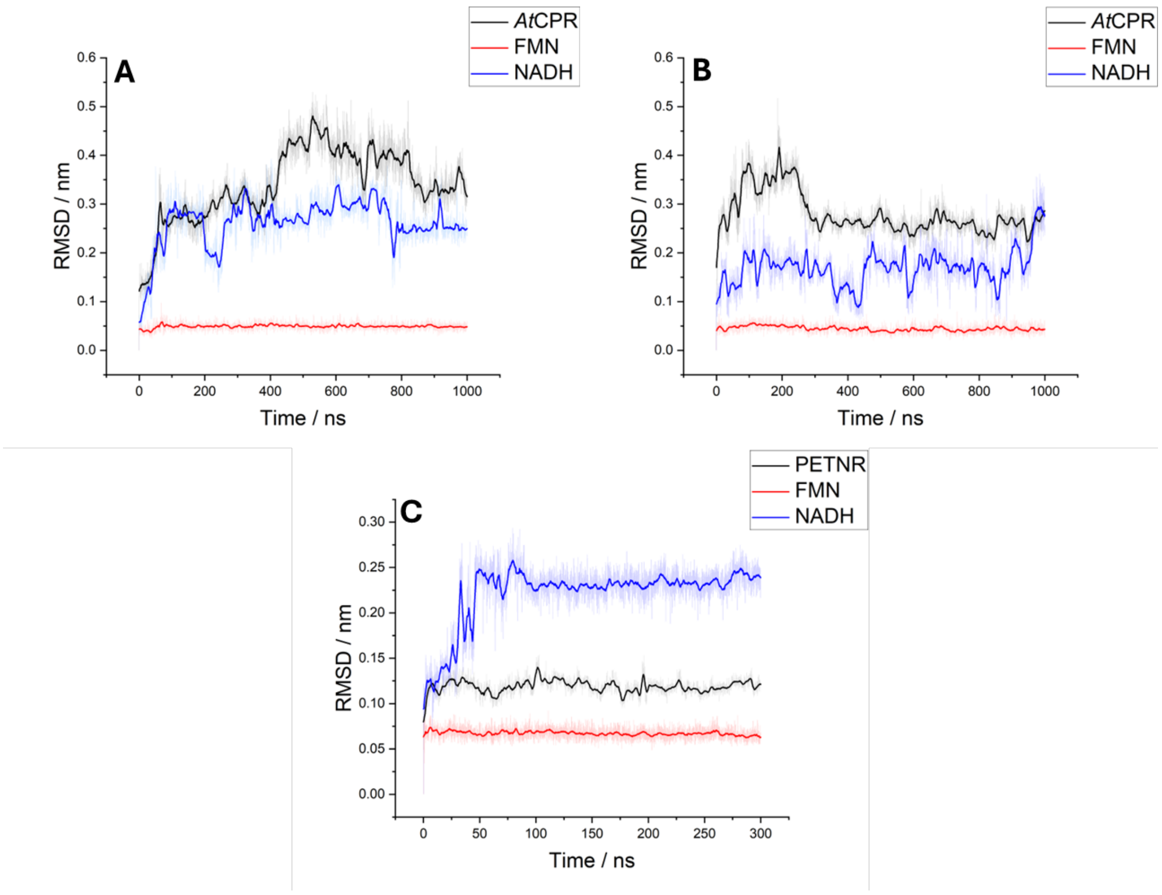

Fig. S11: Backbone RMSDs (protein) and the all-atom RMSDs for FMN and NADH during the production simulation of *At*CPR and PETNR. For *At*CPR, simulations were conducted from two different NADH binding poses that were consistent with either *pro*-*R* or *pro*-*S* hydride transfer. **A:** *At*CPR and an NADH binding pose consistent with *pro*-*R* hydride transfer. **B:** *At*CPR and an NADH binding pose consistent with *pro*-*S* hydride transfer. **C:** PETNR and NADH.

NAD.frcmod

```
MASS

BOND
ca-nv  347.10   1.386       same as ca-nh, penalty score=  0.0
hn-nv  529.50   1.012       same as hn-nh, penalty score=  0.0
c -nt  356.20   1.379       same as  c- n, penalty score=  0.0
hn-nt  527.30   1.013       same as hn- n, penalty score=  0.0

ANGLE
nb-ca-nv  112.100     116.940   same as nb-ca-nh, penalty score=  0.0
ce-c -nt   85.700     115.220   same as ce-c -n , penalty score=  0.0
ca-ca-nv   86.200     120.950   same as ca-ca-nh, penalty score=  0.0
ca-nv-hn   48.800     116.070   same as ca-nh-hn, penalty score=  0.0
c -nt-hn   48.700     117.550   same as c -n -hn, penalty score=  0.0
nt-c -o   113.800     123.050   same as n -c -o , penalty score=  0.0
hn-nv-hn   39.500     115.120   same as hn-nh-hn, penalty score=  0.0
hn-nt-hn   39.000     117.950   same as hn-n -hn, penalty score=  0.0

DIHE
nb-ca-nv-hn   4    4.200       180.000          2.000      same as X -ca-nh-X , penalty score=  0.0
c2-c3-ce-c2   1    0.112         0.000          2.000      same as c2-c2-c3-c2, penalty score=237.0
hc-c3-ce-c2   1    0.360       180.000         -3.000      same as hc-c3-c2-c2
hc-c3-ce-c2   1    1.470         0.000          1.000      same as hc-c3-c2-c2, penalty score=237.0
ce-c -nt-hn   4   10.000       180.000          2.000      same as X -c -n -X , penalty score=  0.0
ca-ca-nv-hn   4    4.200       180.000          2.000      same as X -ca-nh-X , penalty score=  0.0
c2-c3-ce-c    1    0.112         0.000          2.000      same as c2-c2-c3-c2, penalty score=324.0
hc-c3-ce-c    1    0.360       180.000         -3.000      same as hc-c3-c2-c2
hc-c3-ce-c    1    1.470         0.000          1.000      same as hc-c3-c2-c2, penalty score=324.0
o -c -nt-hn   1    2.500       180.000         -2.000      same as hn-n -c -o
o -c -nt-hn   1    2.000         0.000          1.000      same as hn-n -c -o , penalty score=  0.0

IMPROPER
c2-c2-nh-c3         1.1          180.0         2.0          Using the default value
h5-nb-ca-nb         1.1          180.0         2.0          Same as X -X -ca-ha, penalty score= 44.6
(use general term))
ce-h4-c2-nh         1.1          180.0         2.0          Same as X -X -ca-ha, penalty score= 75.4
(use general term))
c -c2-ce-c3         1.1          180.0         2.0          Using the default value
ca-na-ca-nb         1.1          180.0         2.0          Using the default value
ca-ca-ca-nc         1.1          180.0         2.0          Using the default value
c2-c3-c2-ha         1.1          180.0         2.0          Same as X -X -ca-ha, penalty score= 47.1
(use general term))
ca-nb-ca-nv         1.1          180.0         2.0          Using the default value
c2-h4-c2-nh         1.1          180.0         2.0          Same as X -X -ca-ha, penalty score= 75.4
(use general term))
ca-hn-nv-hn         1.1          180.0         2.0          Using the default value
ce-nt-c -o         10.5          180.0         2.0          Using general improper torsional angle  X- X-
c- o, penalty score=  6.0)
c -hn-nt-hn         1.1          180.0         2.0          Same as X -X -n -hn, penalty score=  6.0 (use
general term))
h5-na-cd-nc         1.1          180.0         2.0          Same as X -X -ca-ha, penalty score= 67.5
(use general term))
c3-ca-na-cd         1.1          180.0         2.0          Using the default value
```

NONBON

FMN.frcmod

MASS

BOND
c -ns 356.20   1.379   same as c- n, penalty score= 0.0
hn-ns 527.30   1.013   same as hn- n, penalty score= 0.0

ANGLE
nc-c -ns 110.400   117.110   same as n -c -nc, penalty score= 0.0
c -ns-c   65.600   127.080   same as c -n -c , penalty score= 0.0
c -ns-hn   48.700   117.550   same as c -n -hn, penalty score= 0.0
ns-c -o   113.800   123.050   same as n -c -o , penalty score= 0.0
cd-c -ns   87.100   112.700   same as cd-c -n , penalty score= 0.0

DIHE
nc-c -ns-c   4   10.000   180.000   2.000   same as X -c -n -X , penalty score= 0.0
nc-c -ns-hn   4   10.000   180.000   2.000   same as X -c -n -X , penalty score= 0.0
cd-c -ns-c   4   10.000   180.000   2.000   same as X -c -n -X , penalty score= 0.0
o -c -ns-c   4   10.000   180.000   2.000   same as X -c -n -X , penalty score= 0.0
o -c -ns-hn   1   2.500   180.000   -2.000   same as hn-n -c -o
o -c -ns-hn   1   2.000   0.000   1.000   same as hn-n -c -o , penalty score= 0.0
cd-c -ns-hn   4   10.000   180.000   2.000   same as X -c -n -X , penalty score= 0.0

IMPROPER
nc-ns-c -o   10.5   180.0   2.0   Using general improper torsional angle X- X-c- o, penalty score= 6.0)
c -c -ns-hn   1.1   180.0   2.0   Same as X -X -n -hn, penalty score= 6.0 (use general term))
cd-ns-c -o   10.5   180.0   2.0   Using general improper torsional angle X- X-c- o, penalty score= 6.0)
c -cd-cd-nc   1.1   180.0   2.0   Using the default value
ca-ca-ca-nc   1.1   180.0   2.0   Using the default value
ca-ca-ca-ha   1.1   180.0   2.0   Using general improper torsional angle X- X-ca-ha, penalty score= 6.0)
ca-ca-ca-na   1.1   180.0   2.0   Using the default value
cd-na-cd-nc   1.1   180.0   2.0   Using the default value
c3-ca-na-cd   1.1   180.0   2.0   Using the default value

NONBON

S4. Supplementary results and discussion

S4.1. Control experiments in ^1H_2O

Control experiments were run in ^1H_2O to prove that all deuterium incorporated into NAD(P)H was sourced from ^2H_2O (Table S3). Any ^2H incorporated from the natural abundance of ^2H in ^1H_2O is negligible such that it does not affect the shape of the diagnostic ^1H NMR peak.

Table S3: Control experiments in performed in ^1H_2O.

Entry	Enzyme	Cofactor: NADPH					Cofactor: NADH				
		[4-$^1H_2$] (%)	[4*R*-^{2}H] (%)	[4*S*-^{2}H]- (%)	[4-$^2H_2$]- (%)	NADP$^+$ (%)	[4-$^1H_2$] (%)	[4*R*-^{2}H]- (%)	[4*S*-^{2}H]- (%)	[4-$^2H_2$]- (%)	NAD$^+$ (%)
1	None	99	0	0	0	1	>99	0	0	0	0
2	GR[a]	98	0	0	0	2	-	-	-	-	-
3	MR	-	-	-	-	-	83	0	0	0	17

Reaction conditions: 5 mM NAD(P)H, 10 mM Tris-HCl, pH 8.0, 30 μM flavoenzyme, 37 °C, 24 hours. [a]Reaction carried out with 15 unit/mL enzyme. [^{2}H] determined by ^{1}H NMR on a 400 MHz spectrometer (12.5% ^2H_2O in ^1H_2O, 298 K).

S4.2. Optimisation of reactivity for full HIE of NAD(P)H redox active hydrogen

In efforts to identify flavoenzymes capable of complete deuteration of the redox active hydrogen of NAD(P)H, promising reactions from the initial screening were repeated for 24 hours. The relative proportions of NAD(P)$^+$, undeuterated NAD(P)H, [4*R*-^{2}H]-, [4*S*-^{2}H]- and [4-$^2H_2$]-NAD(P)H were then determined in each of the reaction mixtures (Table S4). Undesired cofactor oxidation (formation of NAD(P)$^+$) remained minimal in all instances. This was also the case in the initial HIE screening experiments.

Table S4: Flavoenzyme-catalysed HIE of NADPH and NADH after 24 hours.

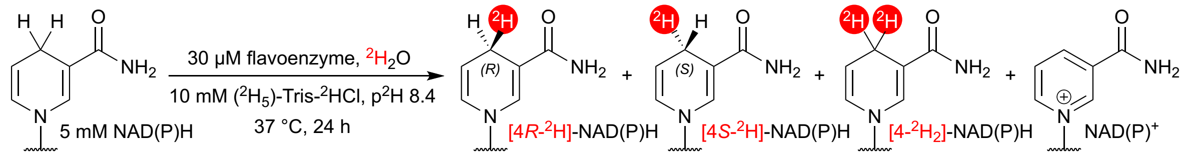

Entry	Enzyme	Cofactor: NADPH					Cofactor: NADH				
		[4-$^1H_2$]- (%)	[4*R*-^{2}H]- (%)	[4*S*-^{2}H]- (%)	[4-$^2H_2$]- (%)	NADP$^+$ (%)	[4-$^1H_2$]- (%)	[4*R*-^{2}H]- (%)	[4*S*-^{2}H]- (%)	[4-$^2H_2$]- (%)	NAD$^+$ (%)
1	None	>99	0	0	0	0	>99	0	0	0	0
2	FAD[a]	89	0	0	0	11	87	0	0	0	13
3	*At*CPR	0	64	0	31	5	-	-	-	-	-
4	*Tt*CPR	3	56	0	37	4	15	16	19	45	5
5	PETNR	0	98	0	0	2	-	-	-	-	-
6	GR[b]	0	0	99	0	1	-	-	-	-	-
7	MR	-	-	-	-	-	6	89	0	0	5
8	OYEC_SCHPO	0	97	0	0	3	-	-	-	-	-
9	YqiG_BACSU	0	98	0	0	2	-	-	-	-	-
10	G0S7C6_CHATD	-	-	-	-	-	1	1	37	46	15

[a]Carried out with 0.5 mM FAD-Na_2. [b]Reaction carried out with 15 unit/mL enzyme. [^{2}H] determined by ^{1}H NMR (400 MHz, ^2H_2O, 298 K).

*At*CPR was able to catalyse HIE of NADPH with an exceptional total level of ^{2}H-incorporation (95%) and a preference for [4*R*-^{2}H]-NADPH. However, a considerable proportion of this ^{2}H-incorporation is attributed to dideuteration product (31%, Table S4, entry 3, blue). In a similar fashion, *Tt*CPR catalysed HIE of NADPH with 93% total ^{2}H-incorporation, 37% of which was [4-$^2H_2$]-NADPH (Table S4, entry 4, blue). Higher levels of dideuterated NADH compared to NADPH shows that *Tt*CPR has less stereochemical control over ^{2}H delivery to NADH compared to NADPH (45% [4-$^2H_2$]-NADH, Table S4, entry 4, orange). The PETNR-catalysed HIE of NADPH resulted in the formation of [4*R*-^{2}H]-NADPH with exceptional ^{2}H-incorporation (98%) and stereoselectivity (Table S4, entry 5, blue). GR was able to catalyse HIE of NADPH with excellent ^{2}H-incorporation (99%) and complete stereoselectivity for [4*S*-^{2}H]-NADPH (Table S4, entry 6, blue). MR-catalysed HIE of NADH gave [4*R*-^{2}H]-NADH with a high level of ^{2}H-incorporation and stereoselectivity (89% [4*R*-^{2}H]-, 0% [4*S*-^{2}H]-, Table S4, entry 7, orange). In the same vein as *At*CPR and PETNR, OYEC_SCHPO and YqiG_BACSU successfully catalysed stereoselective HIE of NADPH to afford [4*R*-^{2}H]-NADPH with 97% and 98% ^{2}H-incorporation, respectively (Table S4, entries 8 and 9, blue). G0S7C6_CHATD

exhibited a preference for [4*S*-^{2}H]-NADH (37%), although a significant proportion of [4-$^2H_2$]-NADH was detected in this reaction mixture (46%, Table S4, entry 10, orange).

S4.3. HIE of 1-methyl-1,4-dihydronicotinamide

The flavoenzyme panel was tested for HIE activity on the biomimetic cofactor 1-methyl-1,4-dihydronicotinamide (MNAH). Under the standard HIE reaction conditions, no deuterated MNAH was detected in any of the reaction mixtures after 24 hours (Fig. S12).

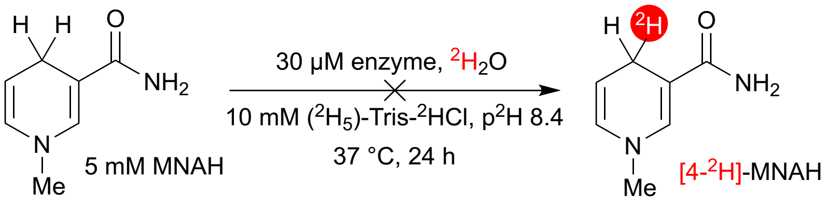

Fig. S12: Incubation of the flavoenzymes with 5 mM MNAH resulted in no incorporation of ^{2}H.

S4.4. Formation of α-NAD(P)$^+$

In the case of the G0S7C6_CHATD-catalysed HIE of NADH, substantial side product formation can be seen on the UPLC trace with UV detection at 260 nm (Fig. S13, retention time = 10.35 mins). In fact, this side product can be observed in all UPLC chromatograms obtained at the end of the 24-hour HIE reactions and is generally most present in samples containing greater proportions of NAD(P)$^+$ (see sections S5.2.1 and S5.2.2 for all UPLC chromatograms). This side product can be discounted as any form of reduced NAD(P)H, since no UV absorbance at 340, 345 or 395 nm was detected (corresponding to 1,4-, 1,6- and 1,2-NAD(P)H, respectively).[20] The mass spectrum corresponding to the side product from the G0S7C6_CHATD reaction chromatogram shows a molecular ion with the same mass as that of NAD$^+$. This led us to identify the side product as the α-anomer of NAD(P)$^+$, which is known to form via NAD(P)H peroxide when NAD(P)H is incubated under anaerobic, slightly alkaline

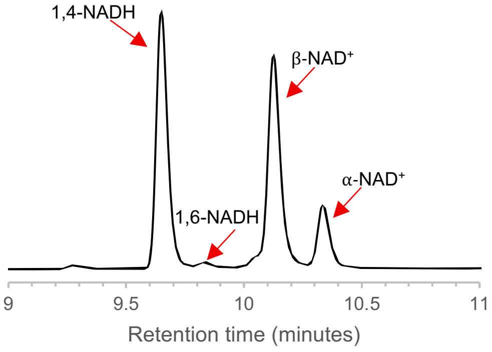

Fig. S13: UPLC chromatogram (UV detection at 260 nm) obtained after 24 hours for the G0S7C6-catalysed HIE of NADH.

conditions.[21] The assignments of signals labelled as 1,4-NADH, β-NAD^+ and α-NAD^+ in fig. S13 were confirmed by comparison of retention times to commercially available standards. The assignment of 1,6-NADH was confirmed by reducing NAD^+ with $NaBH_4$ in 100 mM Tris-HCl, pH 8.5 buffer to give a mixture of 1,4-, 1,6- and 1,2-NADH.[22] This reaction mixture was analysed by UPLC-MS and peaks assigned using known UV absorptions.[23]

S4.5. Formation of 1,6-NAD(P)H

Close inspection of all ^{1}H NMR data reveals side product formation when NAD(P)H is incubated in solution. The singlet at δ = 6.95 ppm and the doublet at δ = 5.85 ppm correspond to H2 and H4 of 1,6-NADH, respectively (Fig. S14A).[24] With regards to NADPH, the singlet at δ = 6.99 ppm and the doublet at δ = 5.92 ppm correspond to H2 and H4 of 1,6-NADPH, respectively (Fig. S14B).

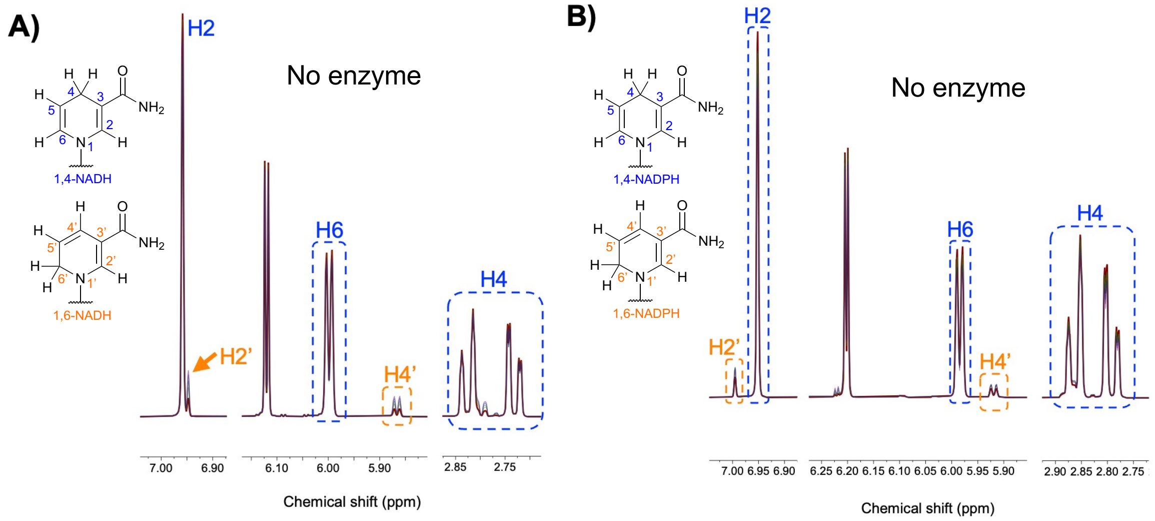

Fig. S14: 800 MHz ^{1}H NMR (^2H_2O, 310 K) spectra showing the signals corresponding to 1,4-NAD(P)H and the formation of 1,6-NAD(P)H over 16 hours. **A)** 1,4- and 1,6-NADH. **B**) 1,4- and 1,6-NADPH.

1,6-NAD(P)H is a biologically inactive isomer of 1,4-NAD(P)H which can form in non-selective cofactor regeneration systems.[25] In our case, 1,6-NAD(P)H formation was found to be non-enzymatic given that it was detected in the controls which contained no enzyme (Fig. S14).

The 800 MHz ^{1}H NMR spectra show new peaks arising in the chemical shift region associated with the redox-active protons (H4) of NAD(P)H (Fig. S15A for NADH, Fig. S15B for NADPH). Although smaller in magnitude, the new signals closely resemble the characteristic splitting pattern of the multiplet corresponding to H4 of 1,4-NADH. These new signals could correspond to H6' of 1,6-NADH. 1,6-NAD(P)H formation is most likely to proceed via a non-enzymatic bimolecular hydride transfer from NAD(P)H to $NAD(P)^+$ (Fig. S15C).[26]

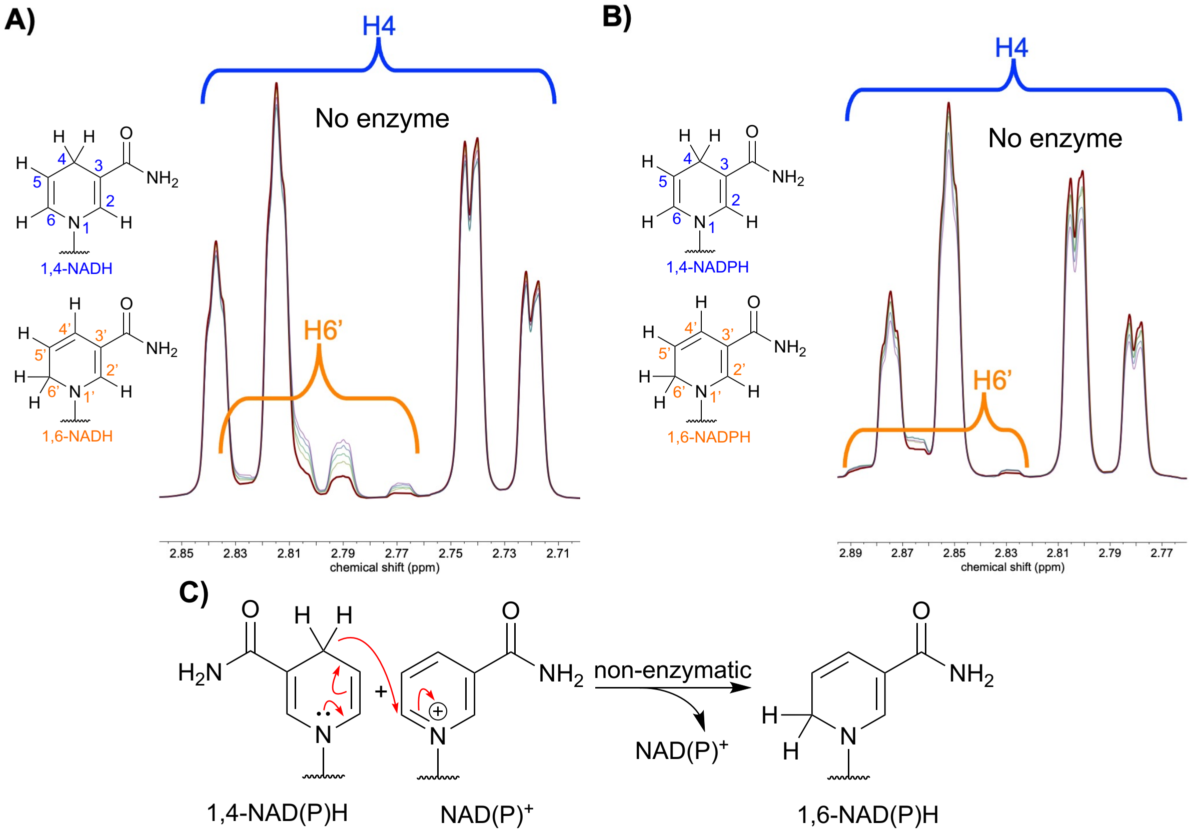

Fig. S15: 800 MHz ^1H NMR (^2H_2O, 310 K) multiplets corresponding to H4 of 1,4-NAD(P)H and H6' of 1,6-NAD(P)H over 16 hours of incubation in ^2H_2O with 10 mM ($^2H_5$)-Tris-^{2}HCl, p^{2}H 8.4. **A)** 1,4- and 1,6-NADH **B)** 1,4- and 1,6-NADPH. **C)** Proposed mechanism for 1,6-NAD(P)H formation.

^{1}H NMR spectra obtained for the enzymatic reactions which resulted in high levels of ^{2}H-incorporation show a collapse of the multiplet corresponding to H6' of 1,6-NAD(P)H, indicating that deuteration occurs in this proton environment. For NADH, this is particularly apparent in the MR-catalysed reaction (Fig. S16A), while for NADPH, this can be seen in the YqiG_BACSU-catalysed reaction (Fig. S16B). The deuterated 1,6-NAD(P)H side product likely forms via a non-enzymatic reaction between [4*R*-^{2}H]-NAD(P)H and NAD(P)$^+$ (Fig. S16C). These peaks appear in other literature reports containing ^{1}H NMR spectra of deuterated NAD(P)H, but they are usually overlooked.[27]

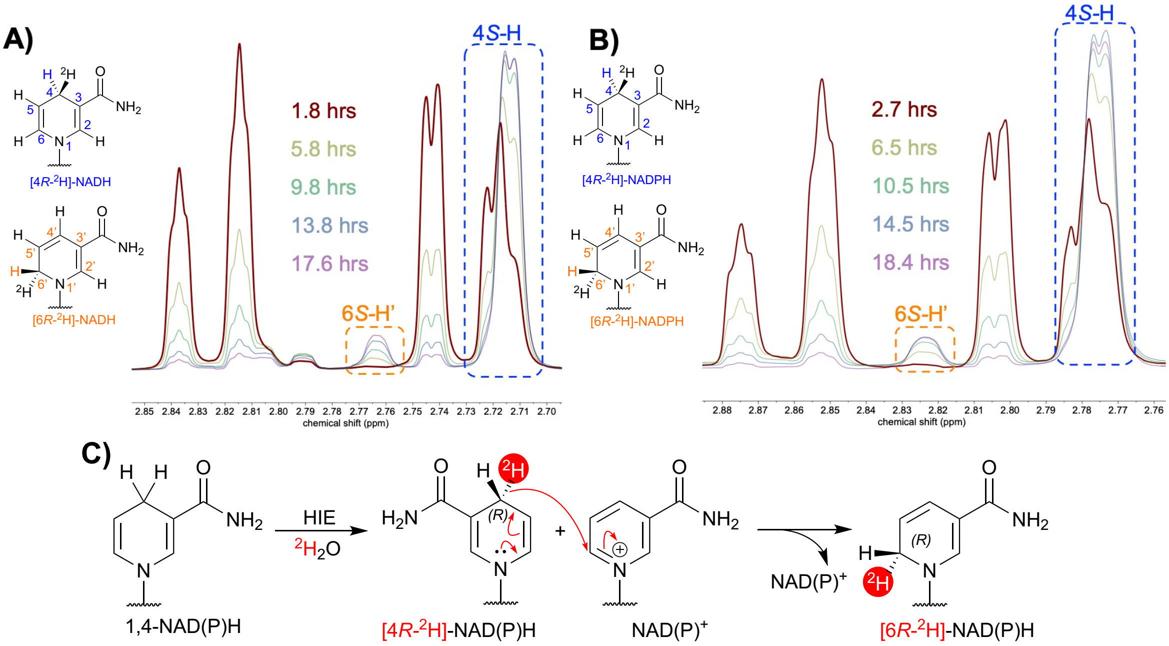

Fig. S16: 800 MHz ^{1}H NMR (^2H_2O, 310 K) time-course showing the formation of [4*R*-^{2}H]-NAD(P)H (major product) and [6*R*-^{2}H]-NAD(P)H (minor product). **A)** MR-catalysed HIE of NADH. **B)** YqiG_BACSU-catalysed HIE of NADPH. **C)** Proposed mechanism for [6*R*-^{2}H]-NAD(P)H side product formation.

The emergence of [6*R*-^{2}H]-NADH ^{1}H NMR signals which correspond to all protons of the dihydronicotinamide ring is shown over time in Fig. S17.

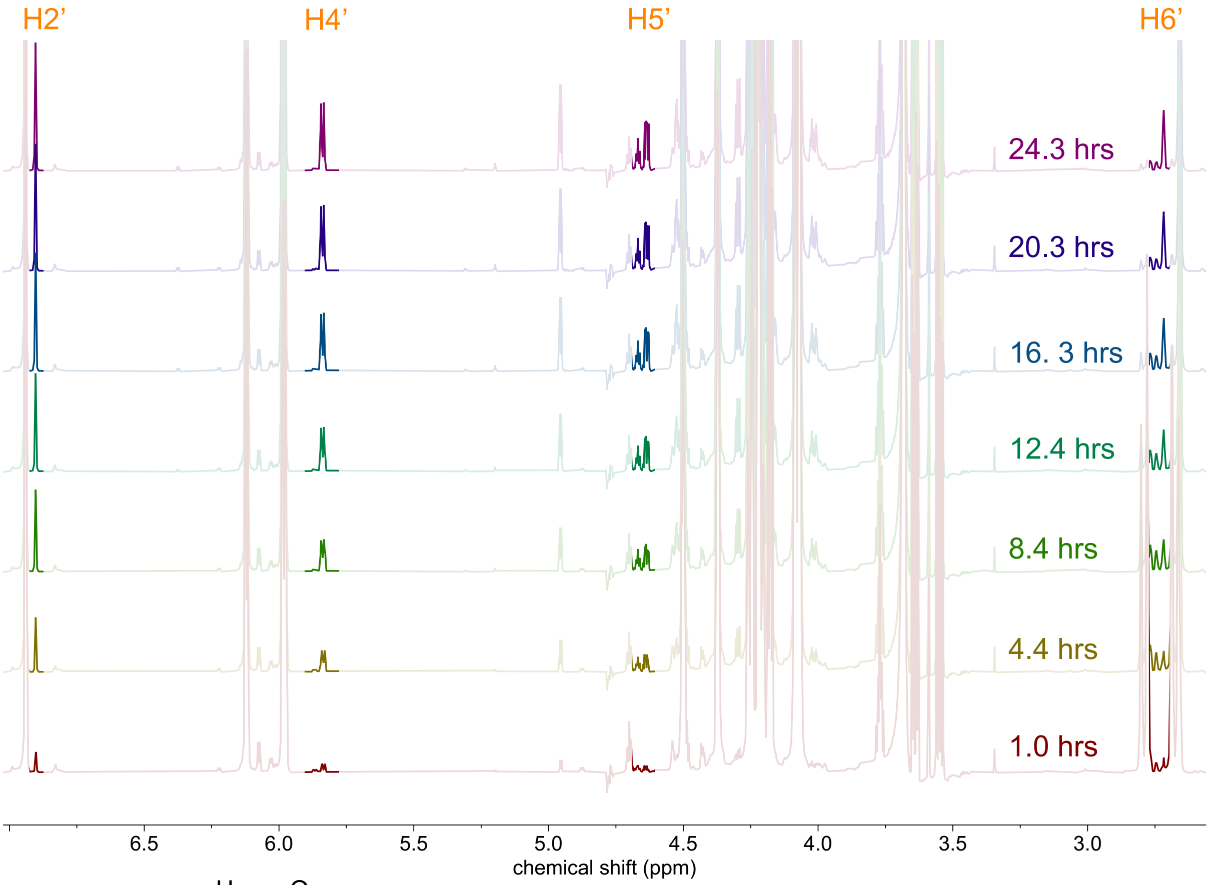

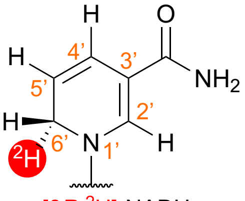

[6*R*-^{2}H]-NADH

Proton assignment	Chemical shift range (ppm)	∫ (Normalised)	∫ (Absolute)
H2'	6.92 - 6.89	1.0000	24380.4926
H4'	5.85 - 5.83	1.0402	25360.7338
H5'	4.68 - 4.62	1.1353	27680.0495
H6'	2.77 - 2.71	1.1201	27309.6477

Fig. S17: Emergence and associated integration of ^{1}H NMR (800 MHz, ^2H_2O, 310 K) signals corresponding to the protons of the dihydronicotinamide ring of [6*R*-^{2}H]-NADH formed in the MR-catalysed HIE of NADH.

An equimolar mixture of NADH and NAD$^+$ (5 mM) was incubated in ^2H_2O containing 10 mM ($^2H_5$)-Tris-^{2}HCl at 37 °C for 24 hours. Two-dimensional ^{1}H correlation spectroscopic analysis (COSY) of this reaction mixture shows coupling between the H6' and H5' protons, thereby confirming the assignment of these signals to the 1,6-isomer rather than the other possible inactive isomer of NADH (1,2-NADH) (Fig. S18). ^4J allylic coupling can also be observed between H6' and H4'.

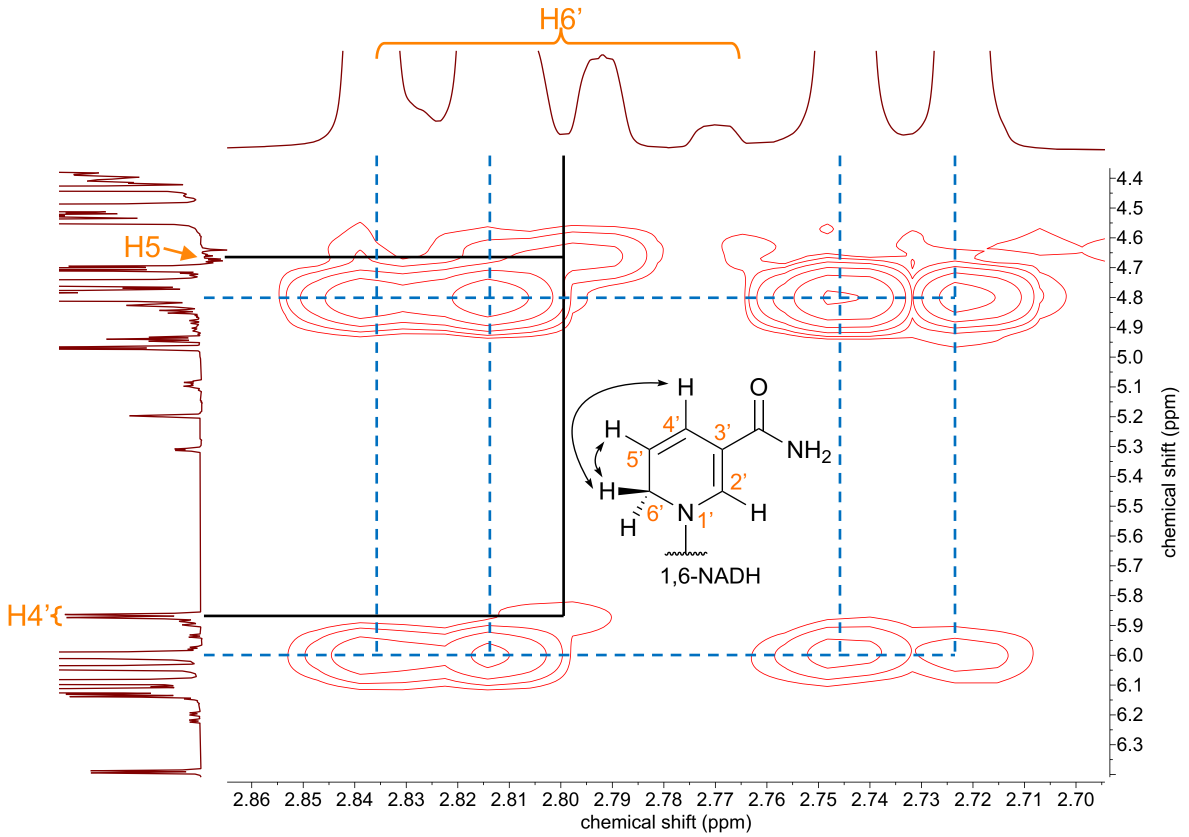

Fig. S18: ^{1}H COSY (800 MHz, $^{2}H_2O$, 310 K) spectrum showing the coupling of the H6' protons to H5' and H4' of 1,6-NADH. Coupling of protons in the dihydronicotinamide ring of 1,6-NADH is shown by the solid black lines. Coupling of protons from 1,4-NADH is shown by the dashed blue lines.

It should be noted that, under the HIE conditions, 1,6-NAD(P)H formation occurred in negligible amounts such that it did not significantly reduce the purity of the desired 1,4-isomer.

S4.6. Truncation of *At*CPR for the removal of [2Fe-2S] cluster

To assess whether the [2Fe-2S] cluster of the CPRs play any role in the HIE reaction between $^{2}H_2O$ and NAD(P)H, *At*CPR was expressed without its [2Fe-2S] cluster (*At*CPR-FMN) and subjected to the standard HIE reaction conditions on NADPH. No significant differences in product formation were observed between the *At*CPR-FMN- and full-length *At*CPR-catalysed reactions after 24 hours of incubation at 37 °C (Table S5).

Table S5: Comparison of full-length *At*CPR- and *At*CPR-FMN-catalysed HIE on NADPH.

		Cofactor: NADPH				
**Entry**	**Enzyme**	**[$^{1}H_2$] (%)**	**[4*R*-^{2}H]- (%)**	**[4*S*-^{2}H]- (%)**	**[4-$^{2}H_2$]- (%)**	**NADP^{+} (%)**
1	Full-length *At*CPR	0	64	0	31	5
2	*At*CPR-FMN	4	65	0	25	6

Reaction conditions: 5 mM NADPH, 10 mM ($^{2}H_5$)-Tris-^{2}HCl, $^{2}H_2O$, p^{2}H 8.4, 30 µM flavoenzyme, 37 °C, 24 hours. [^{2}H] determined by ^{1}H NMR on a 400 MHz spectrometer ($^{2}H_2O$, 298 K).

S5. Supplementary data

S5.1. HIE reaction time-course

S5.1.1. Stereochemical outcome of HIE over time

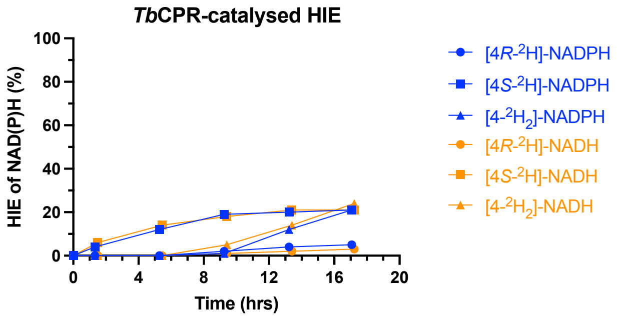

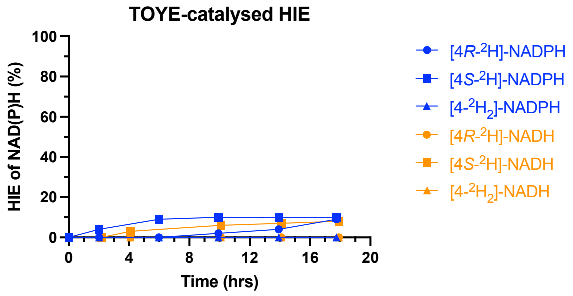

S5.1.2. ^{1}H NMR (800 MHz) data

<u>PETNR-catalysed HIE of NADPH</u>

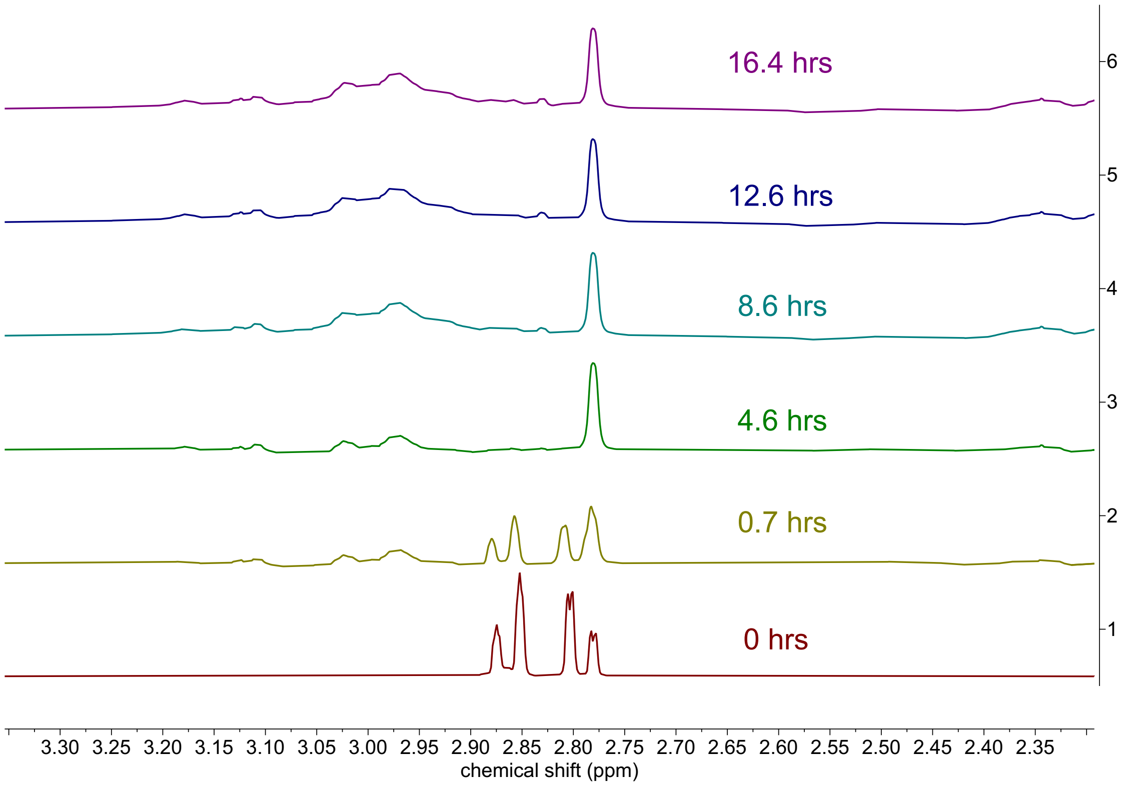

PETNR-catalysed HIE of NADH

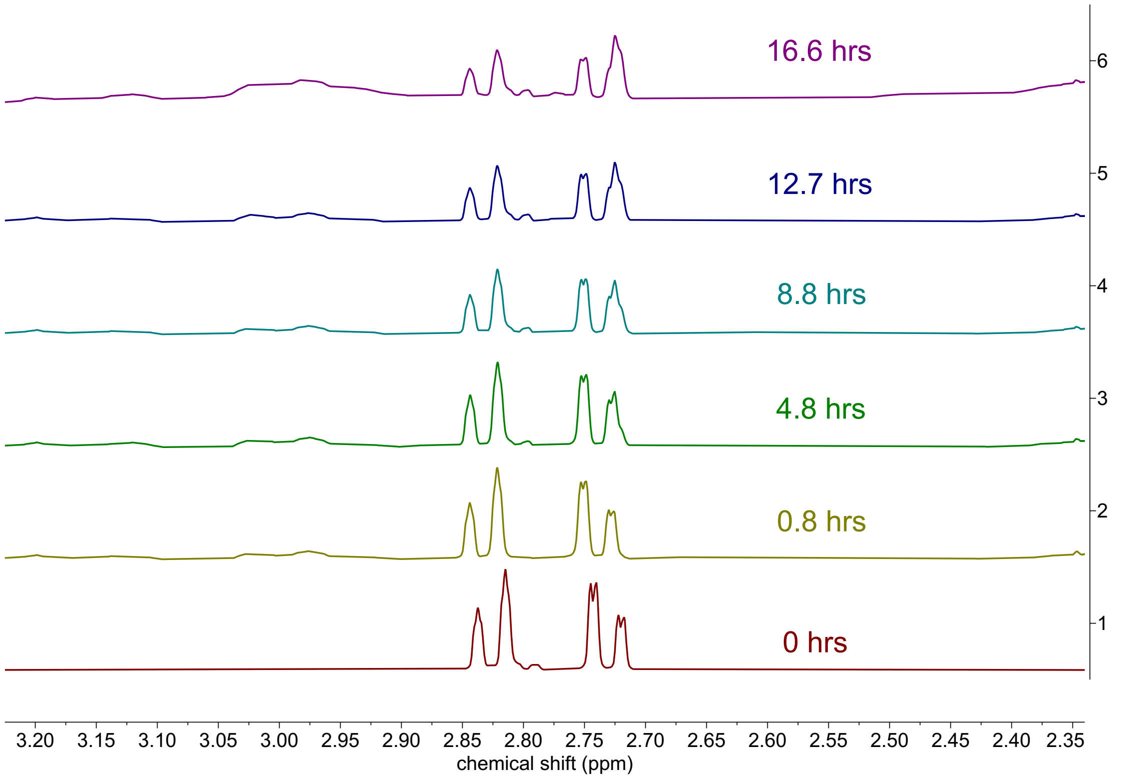

GR-catalysed HIE of NADPH

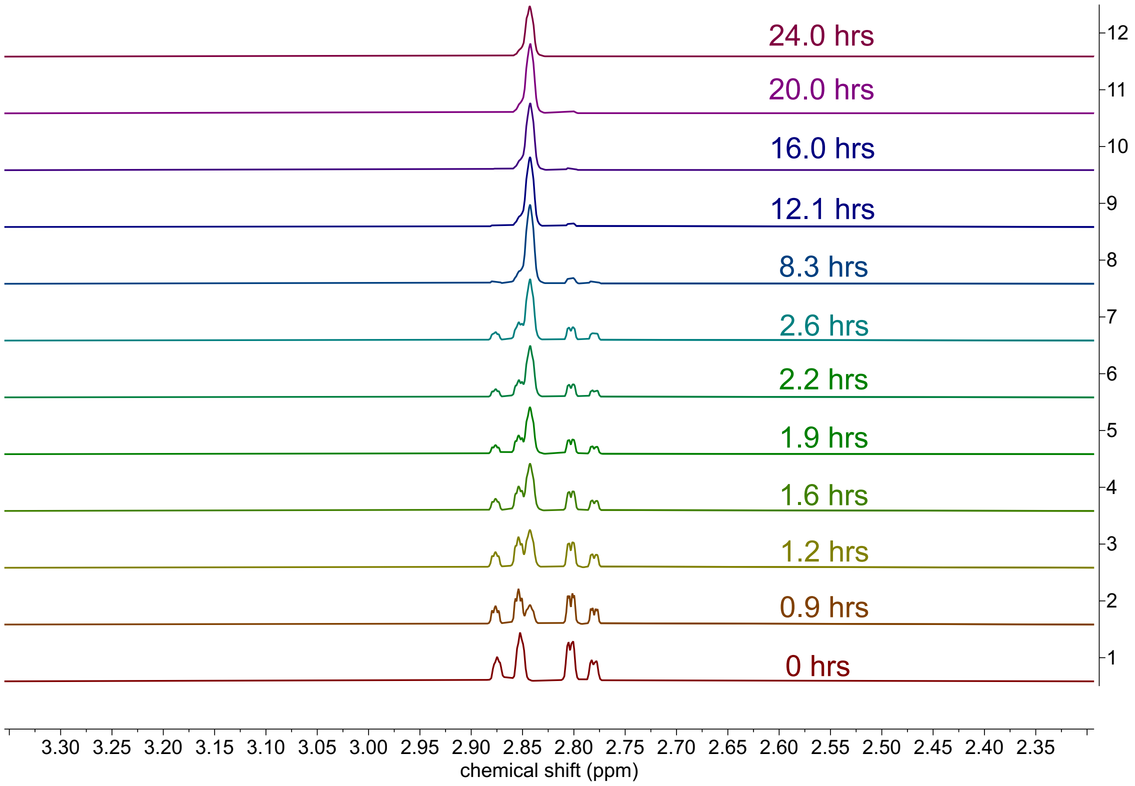

GR-catalysed HIE of NADH

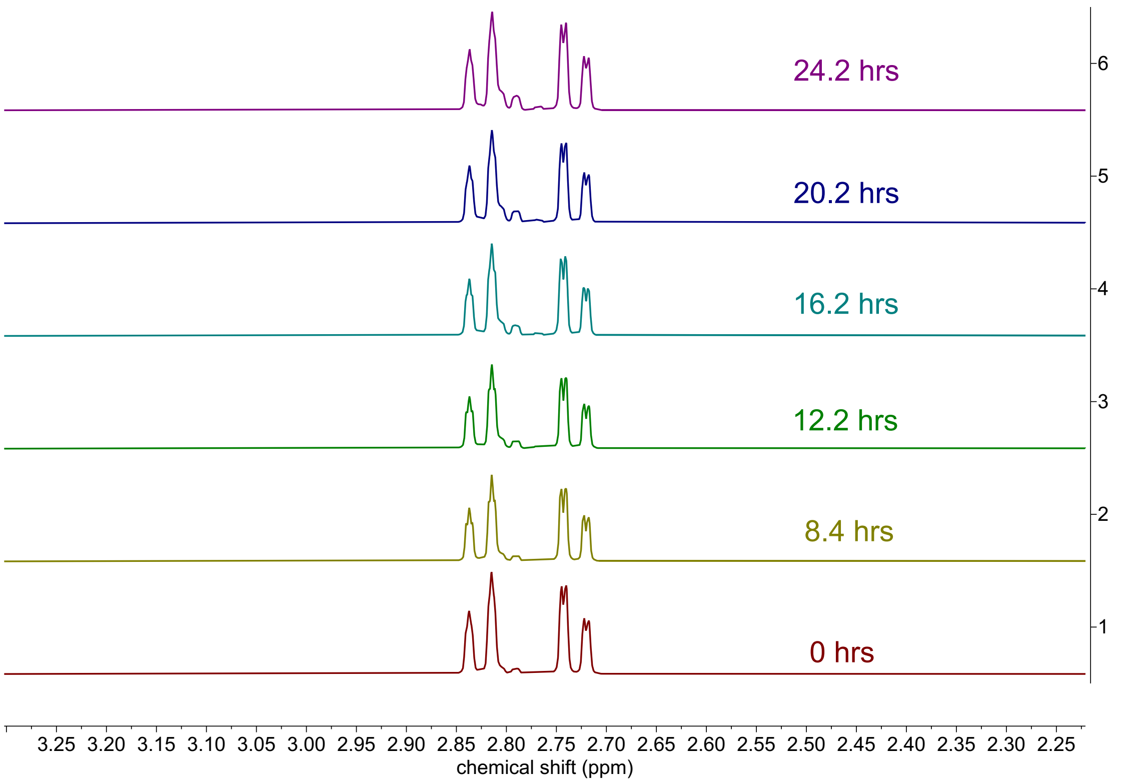

*At*CPR-catalysed HIE of NADPH

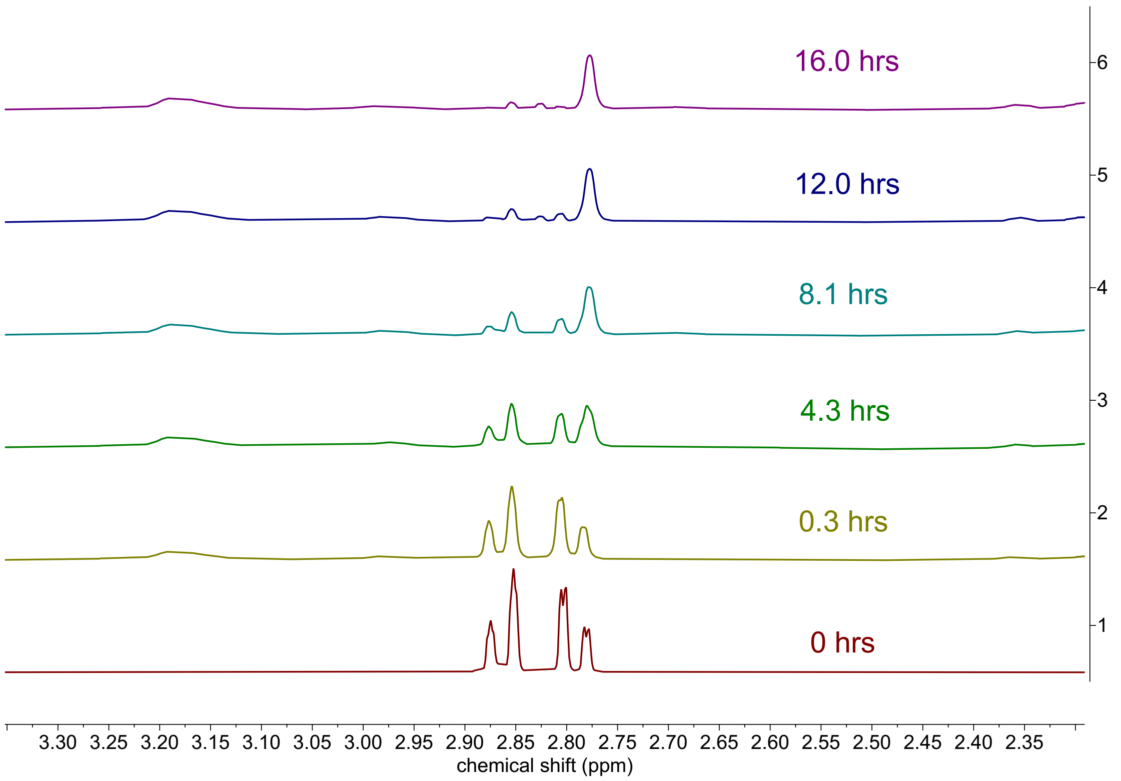

*At*CPR-catalysed HIE of NADH

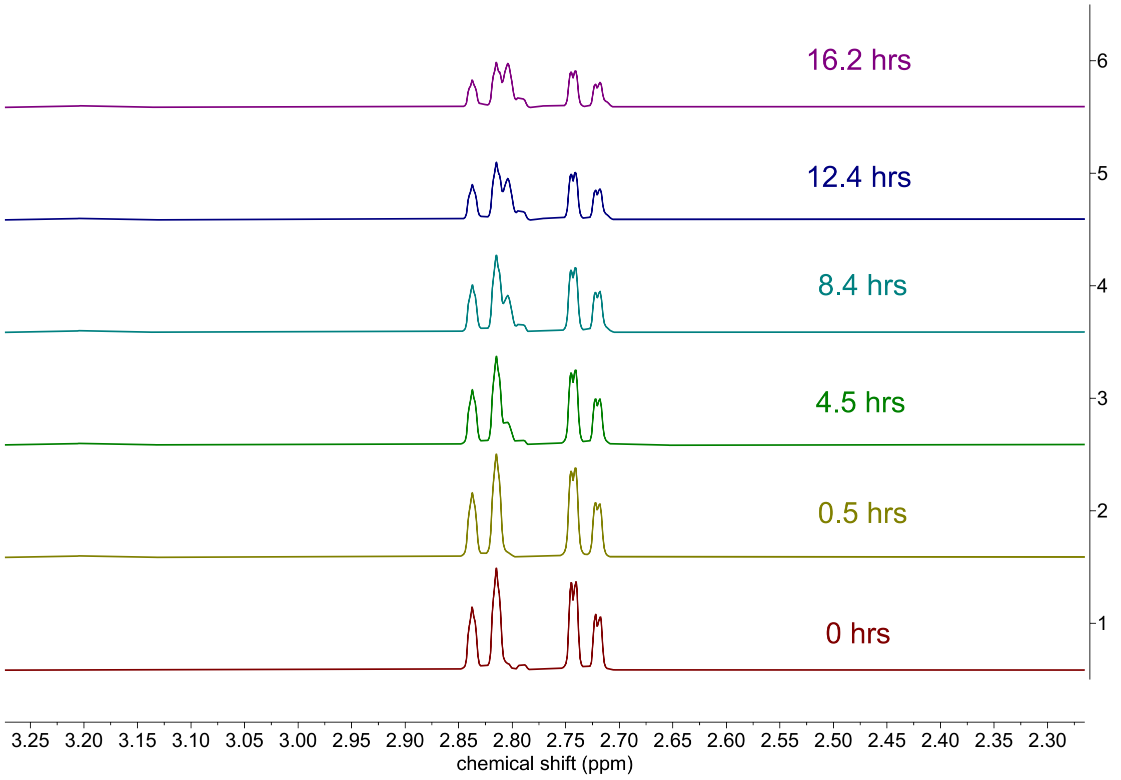

*Tb*CPR-catalysed HIE of NADPH

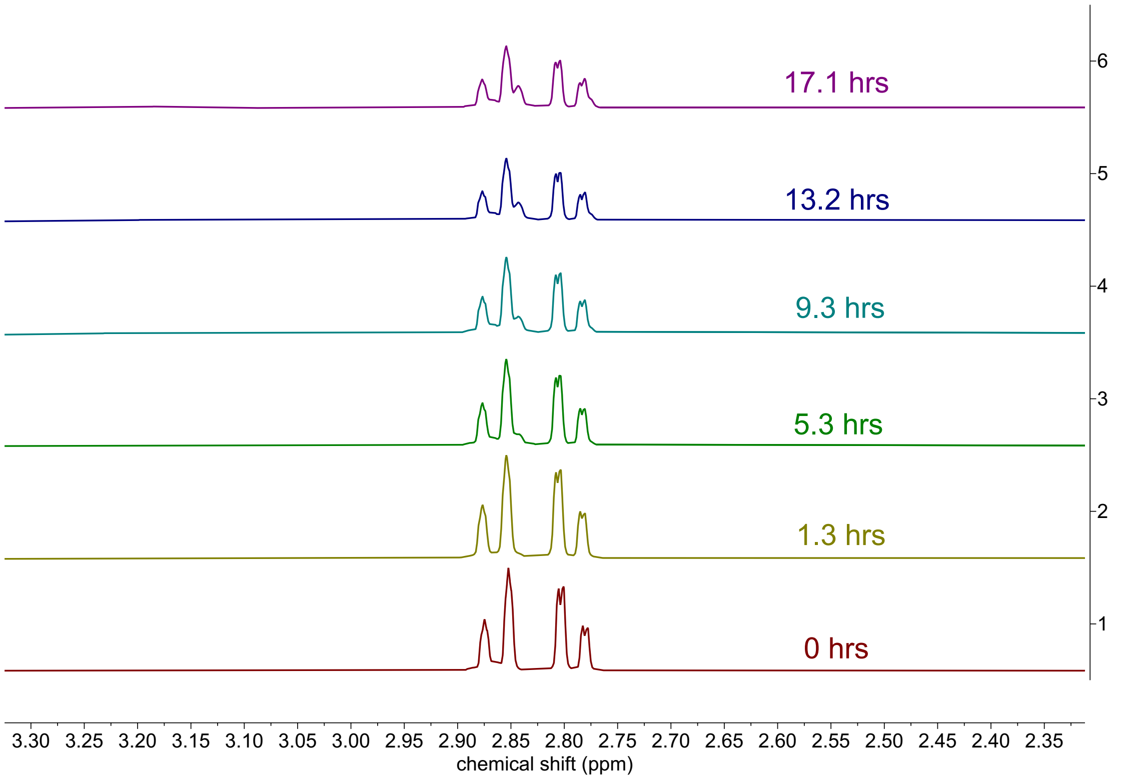

<u>*Tb*CPR-catalysed HIE of NADH</u>

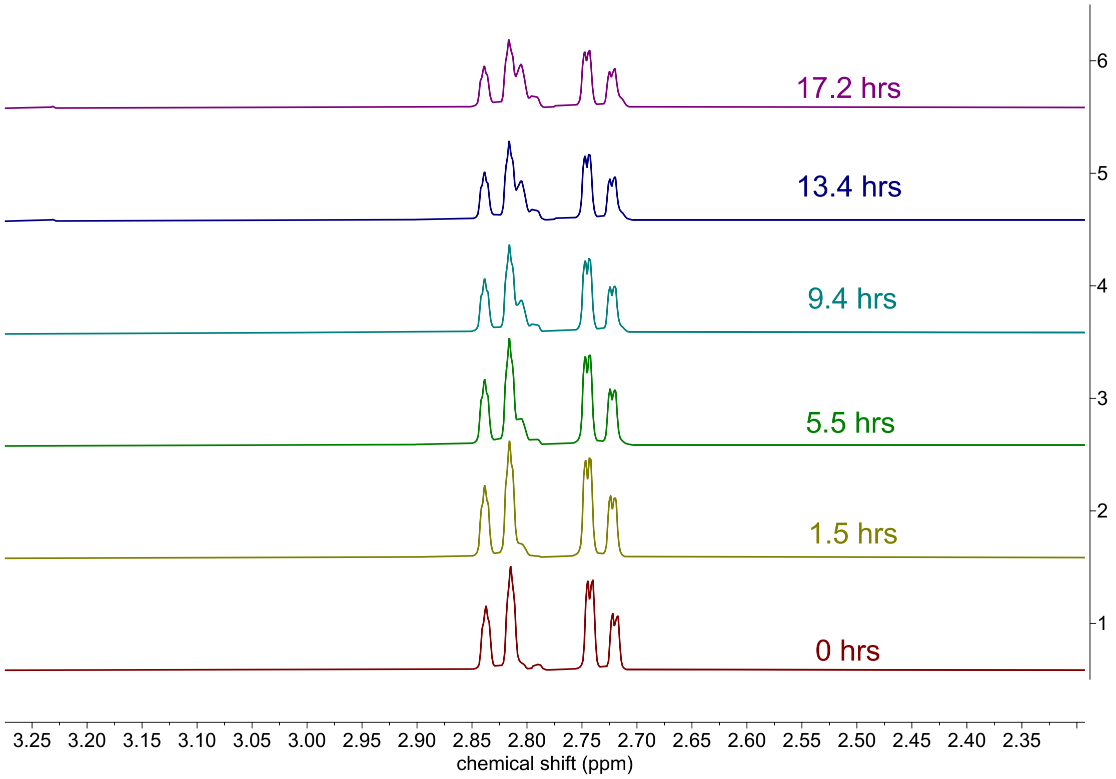

<u>*Tt*CPR-catalysed HIE of NADPH</u>

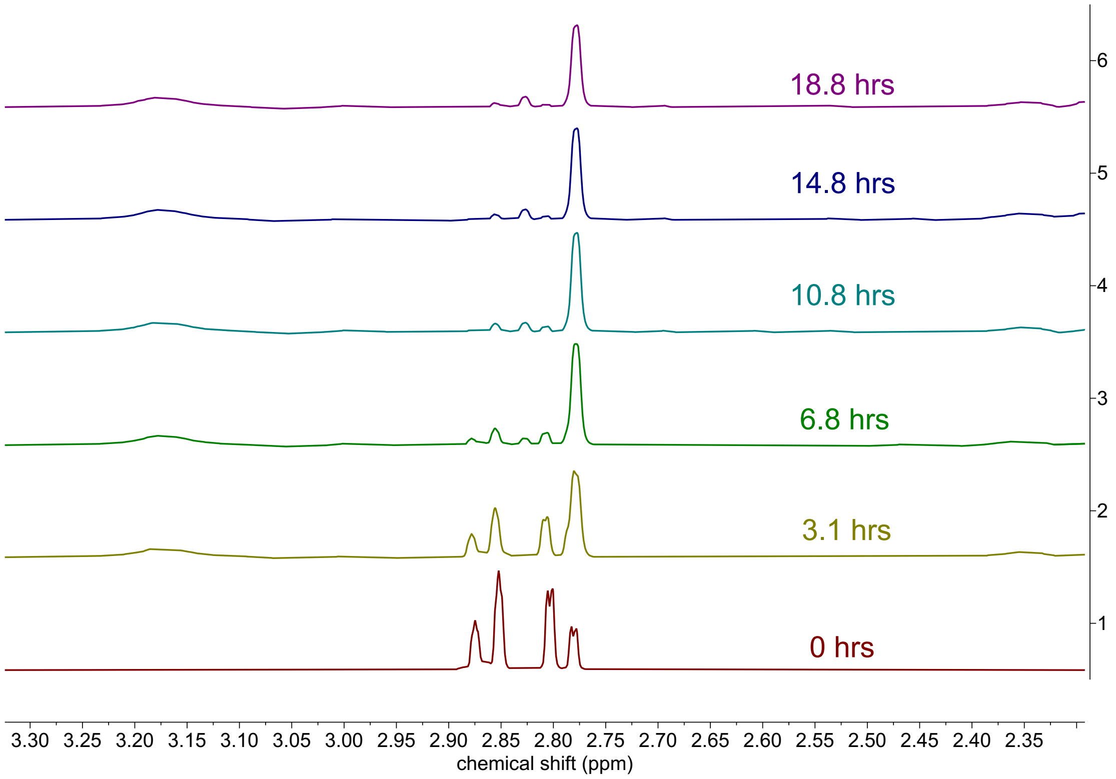

<u>*Tt*CPR-catalysed HIE of NADH</u>

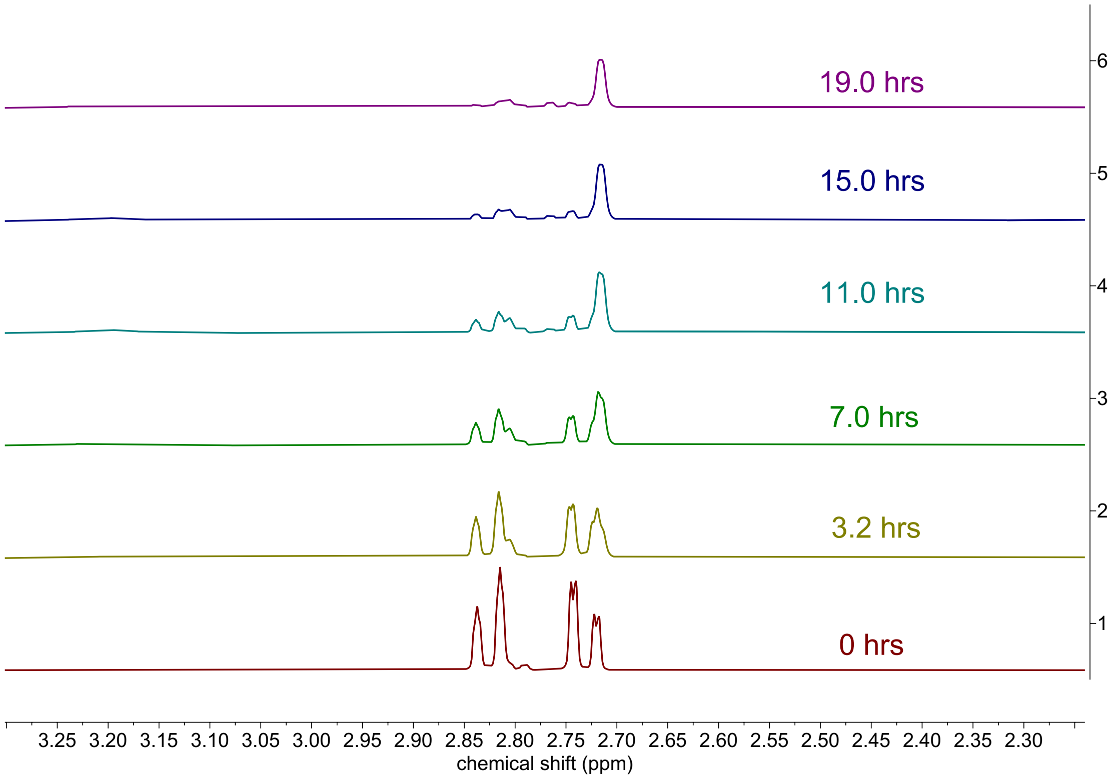

<u>MR-catalysed HIE of NADPH</u>

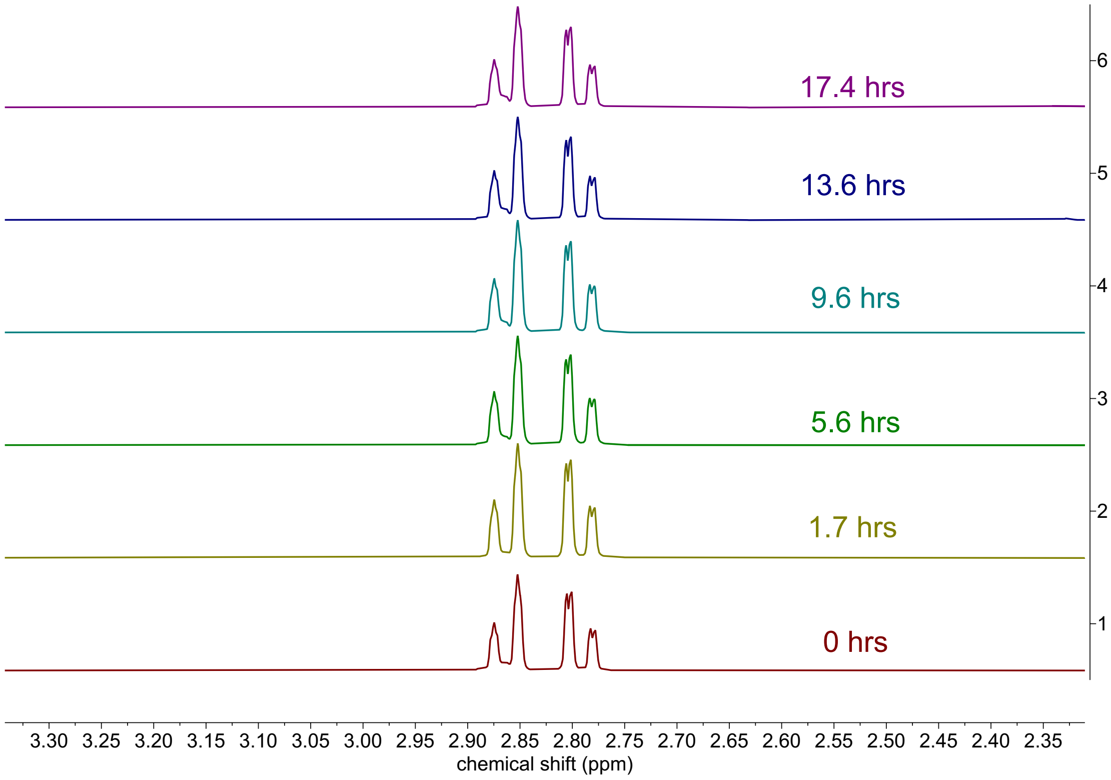

MR-catalysed HIE of NADH

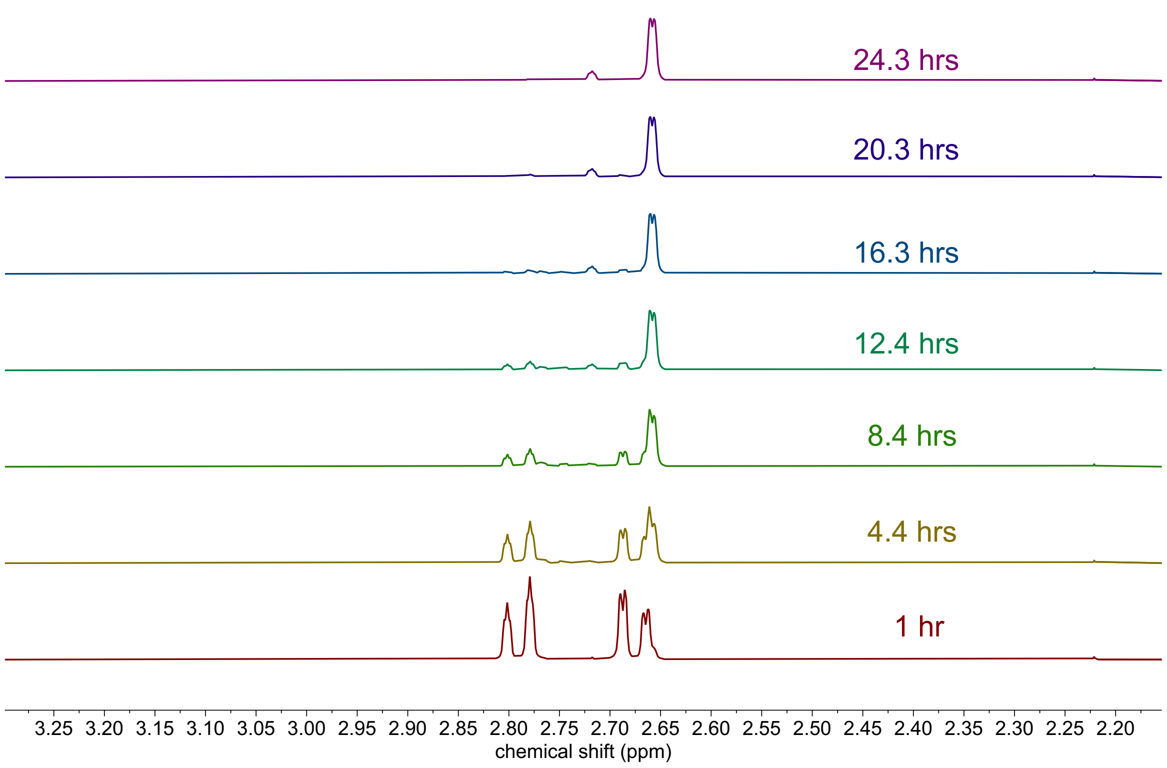

OYEC_SCHPO-catalysed HIE of NADPH

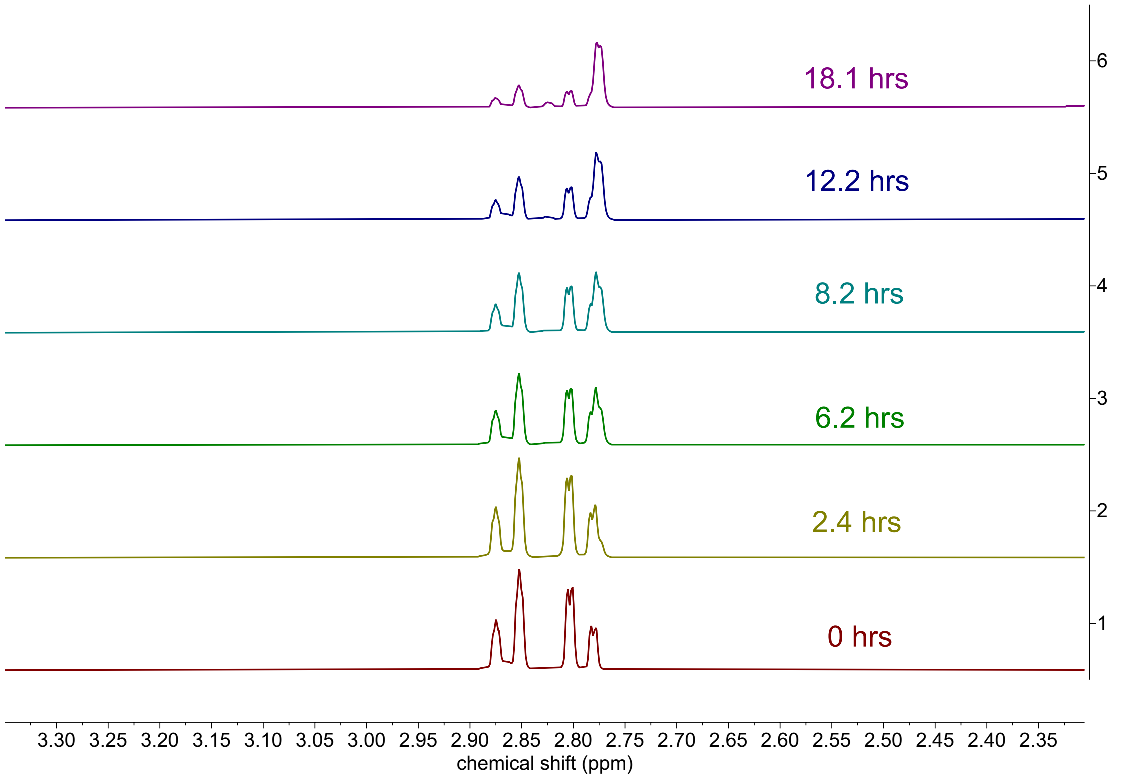

OYEC_SCHPO-catalysed HIE of NADH

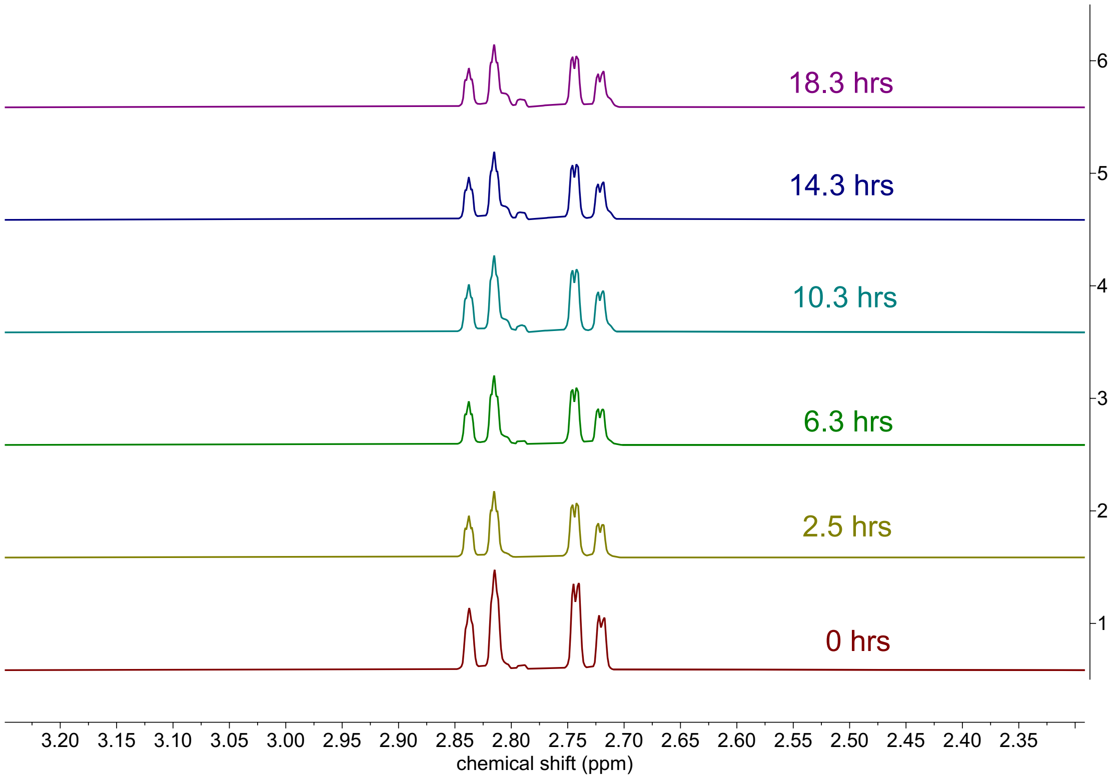

NADH diaphorase-catalysed HIE of NADPH

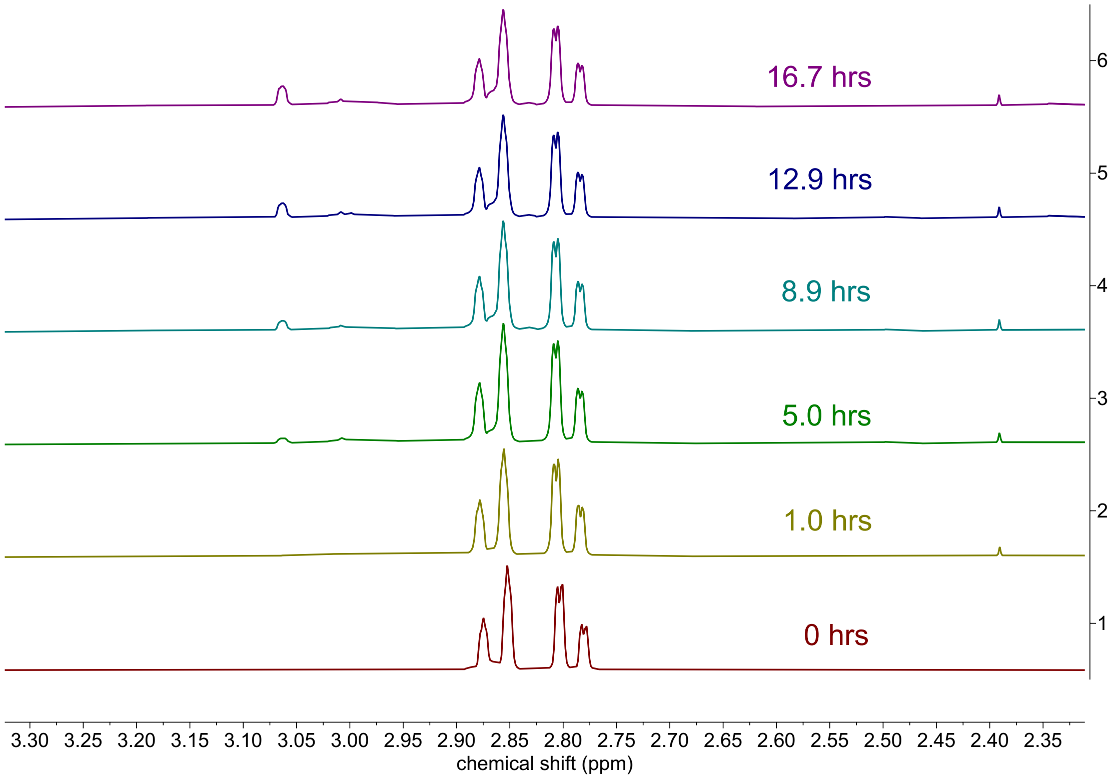

NADH diaphorase-catalysed HIE of NADH

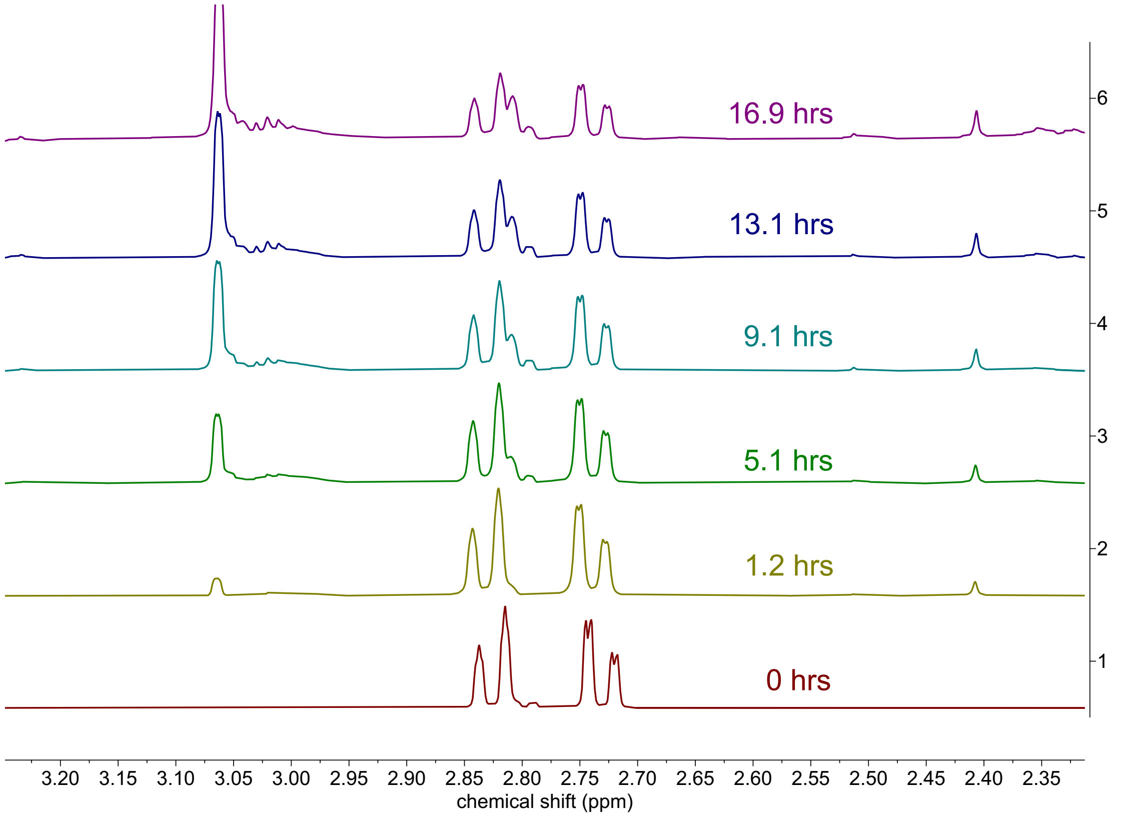

TOYE-catalysed HIE on NADPH

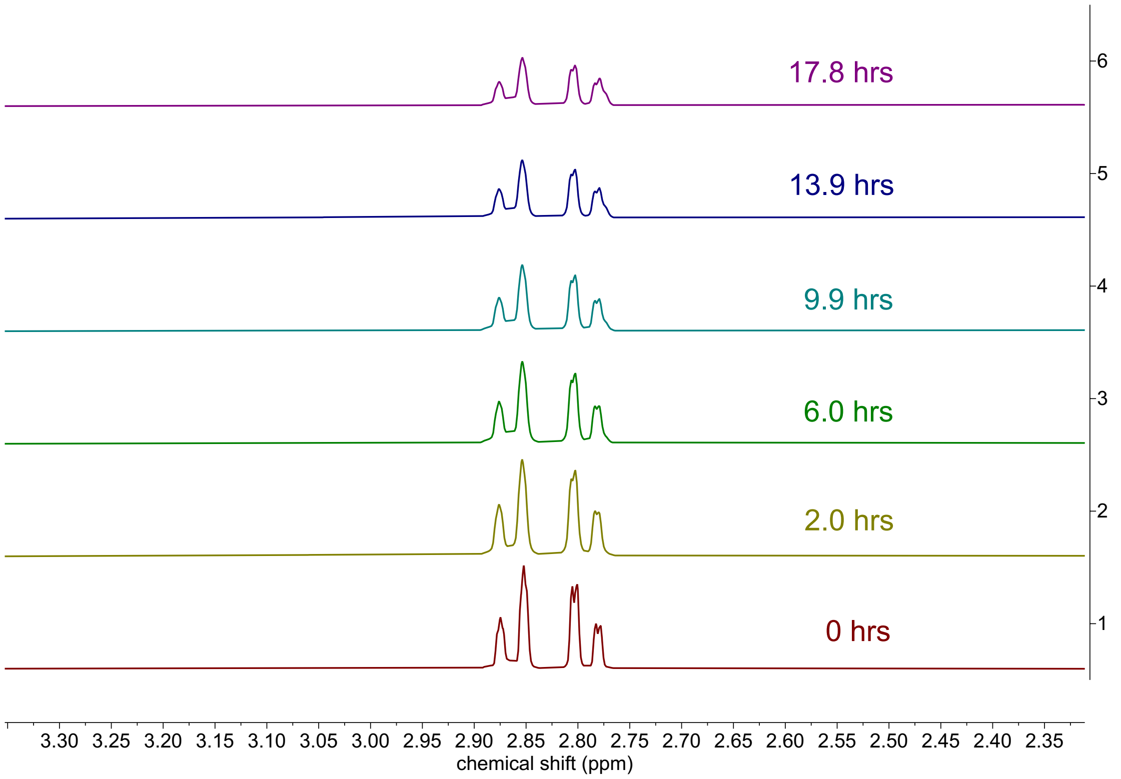

TOYE-catalysed HIE on NADH

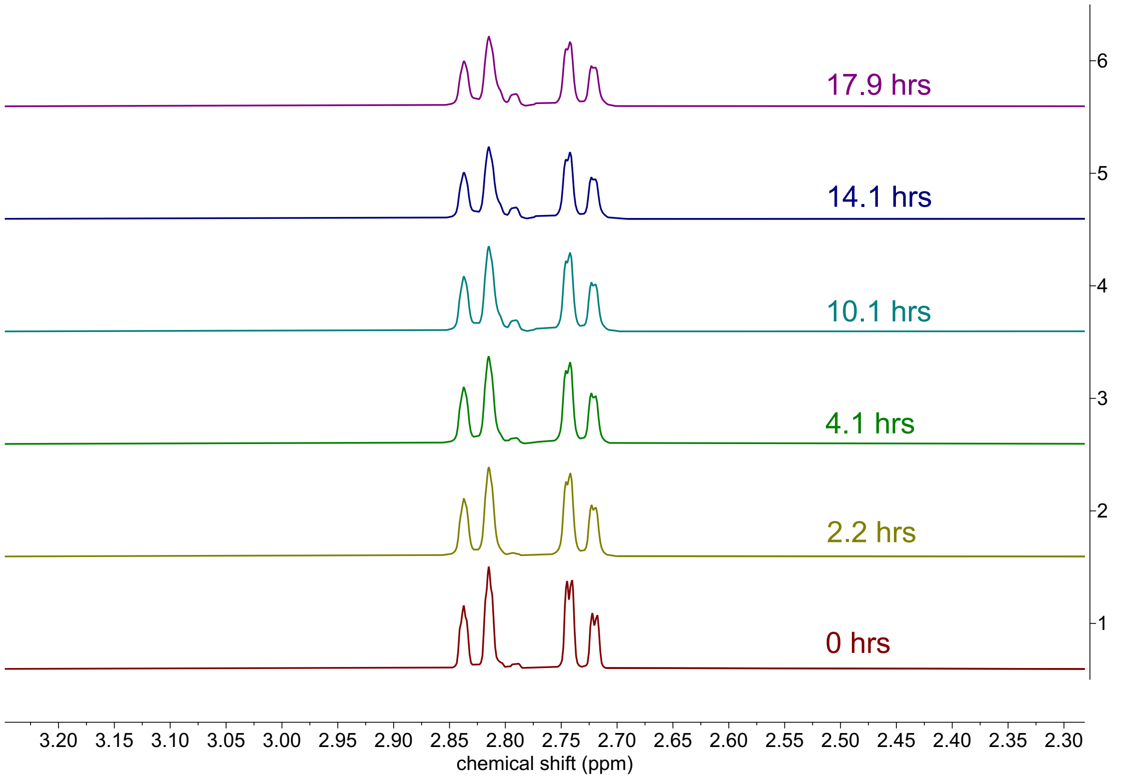

YqiG_BACSU-catalysed HIE on NADPH

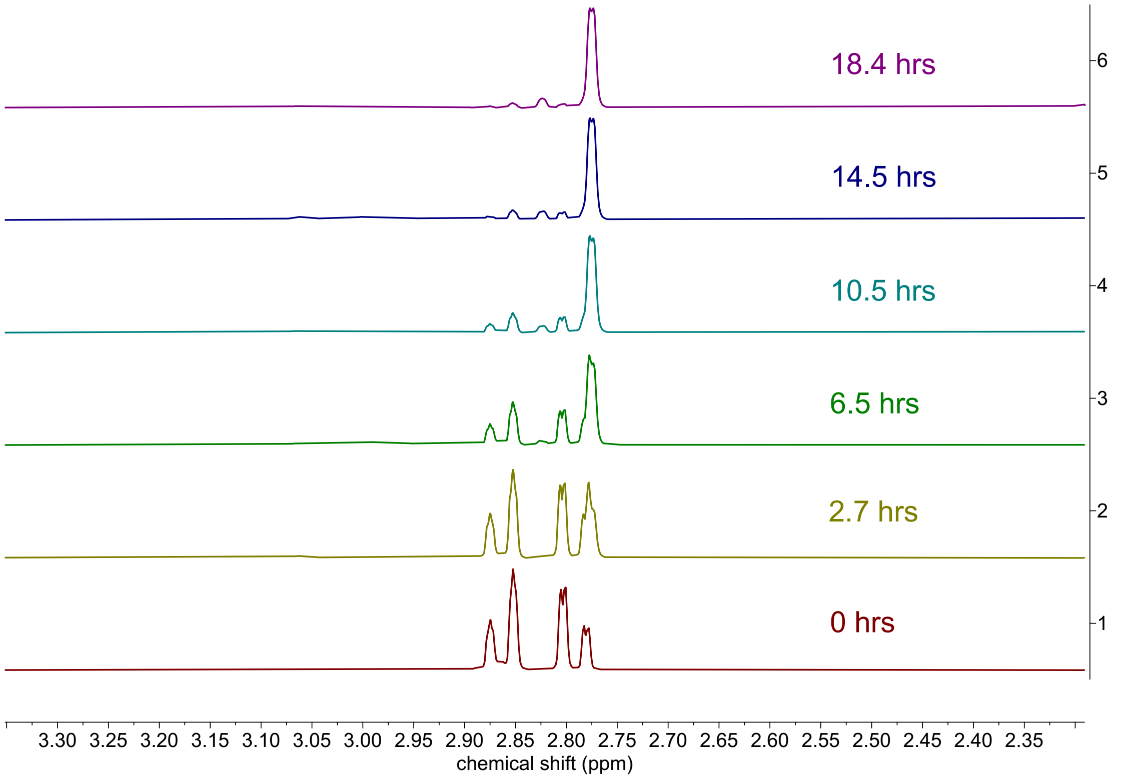

YqiG_BACSU-catalysed HIE on NADH

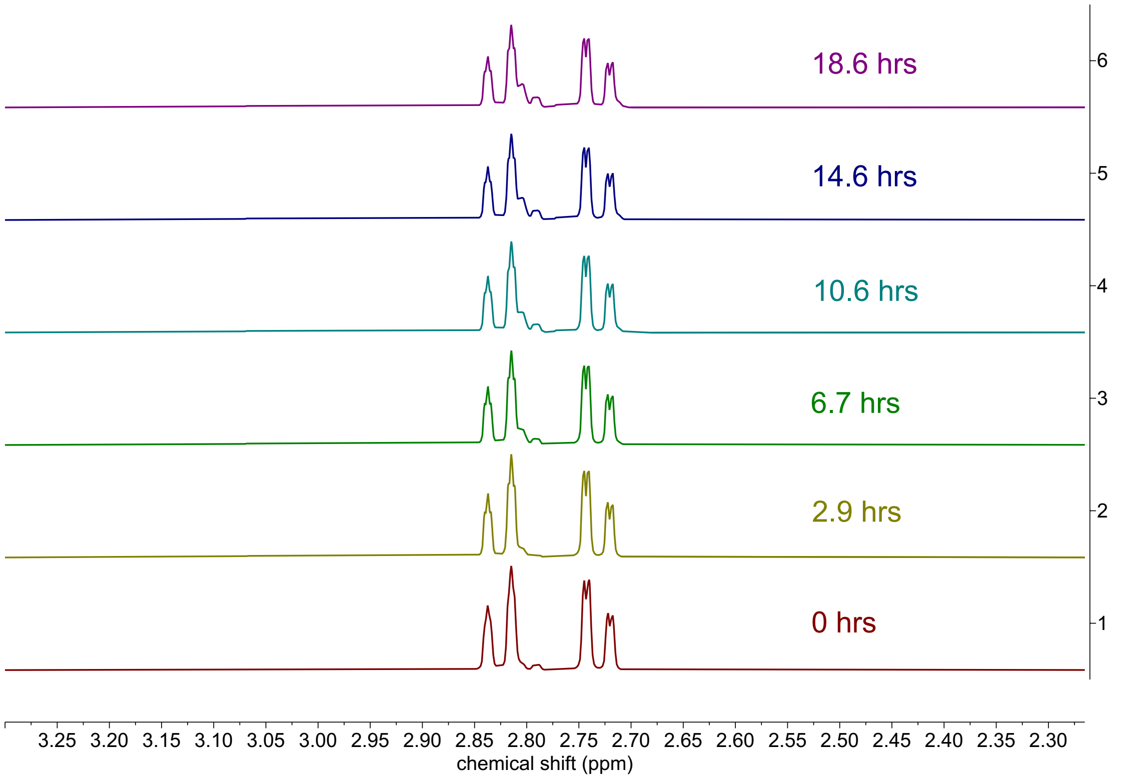

G0S7C6_CHATD-catalysed HIE on NADPH

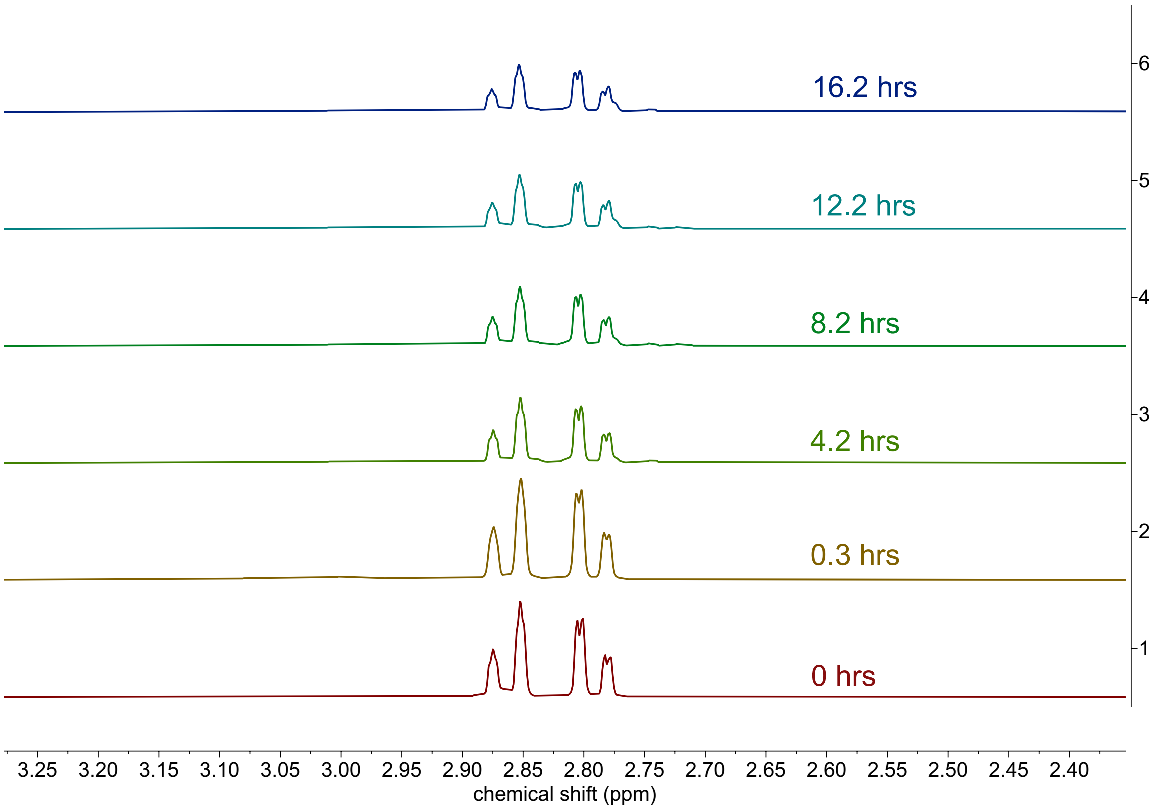

G0S7C6_CHATD-catalysed HIE on NADH

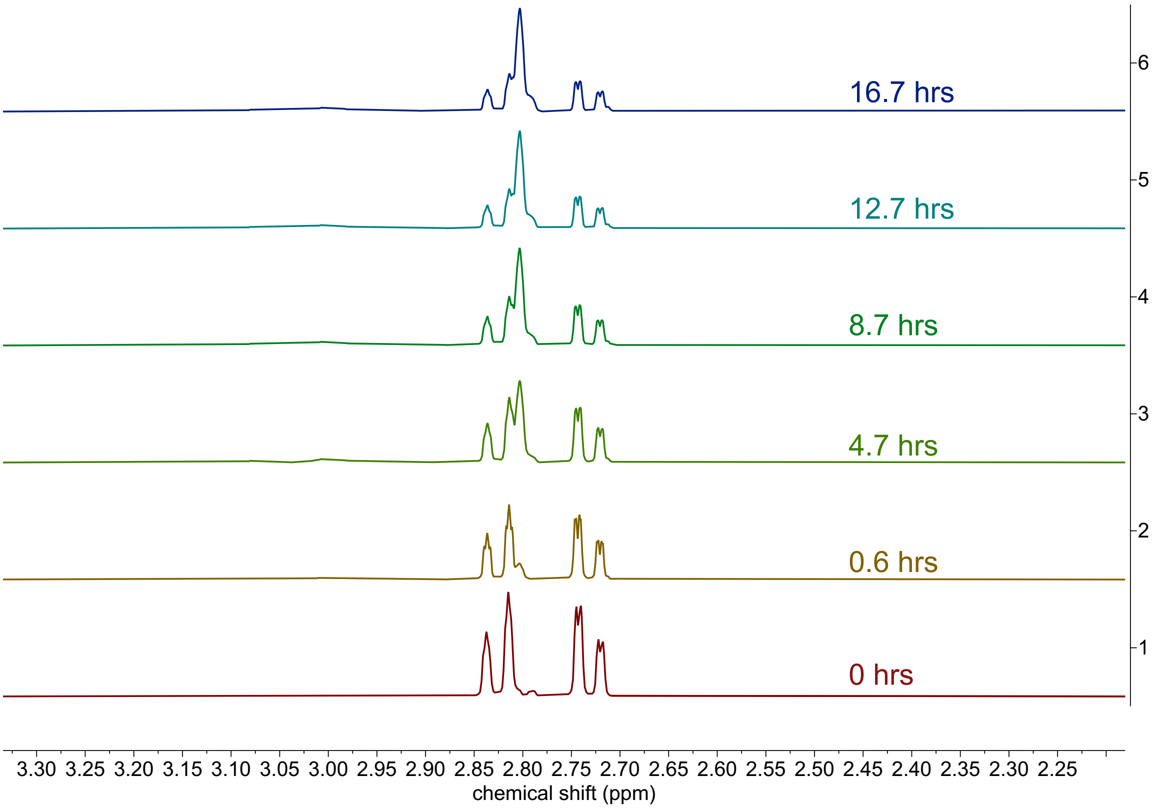

S5.2. 24-hour HIE reactions

S5.2.1. UPLC-MS data – HIE of NADPH

	UV absorbance at 260 nm	UV absorbance at 340 nm	Mass detection (ESI) negative ion mode
β-NADP⁺ standard	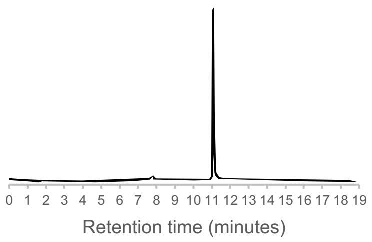	-	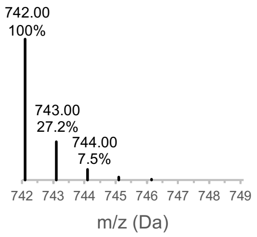
1,4-NADPH standard	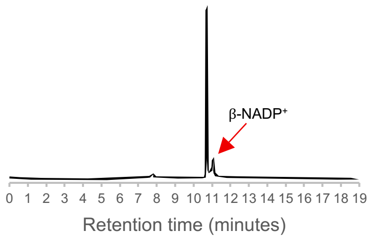	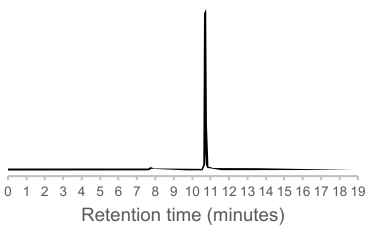	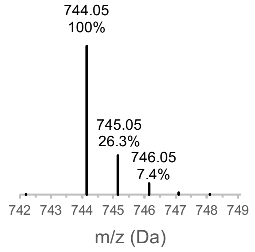

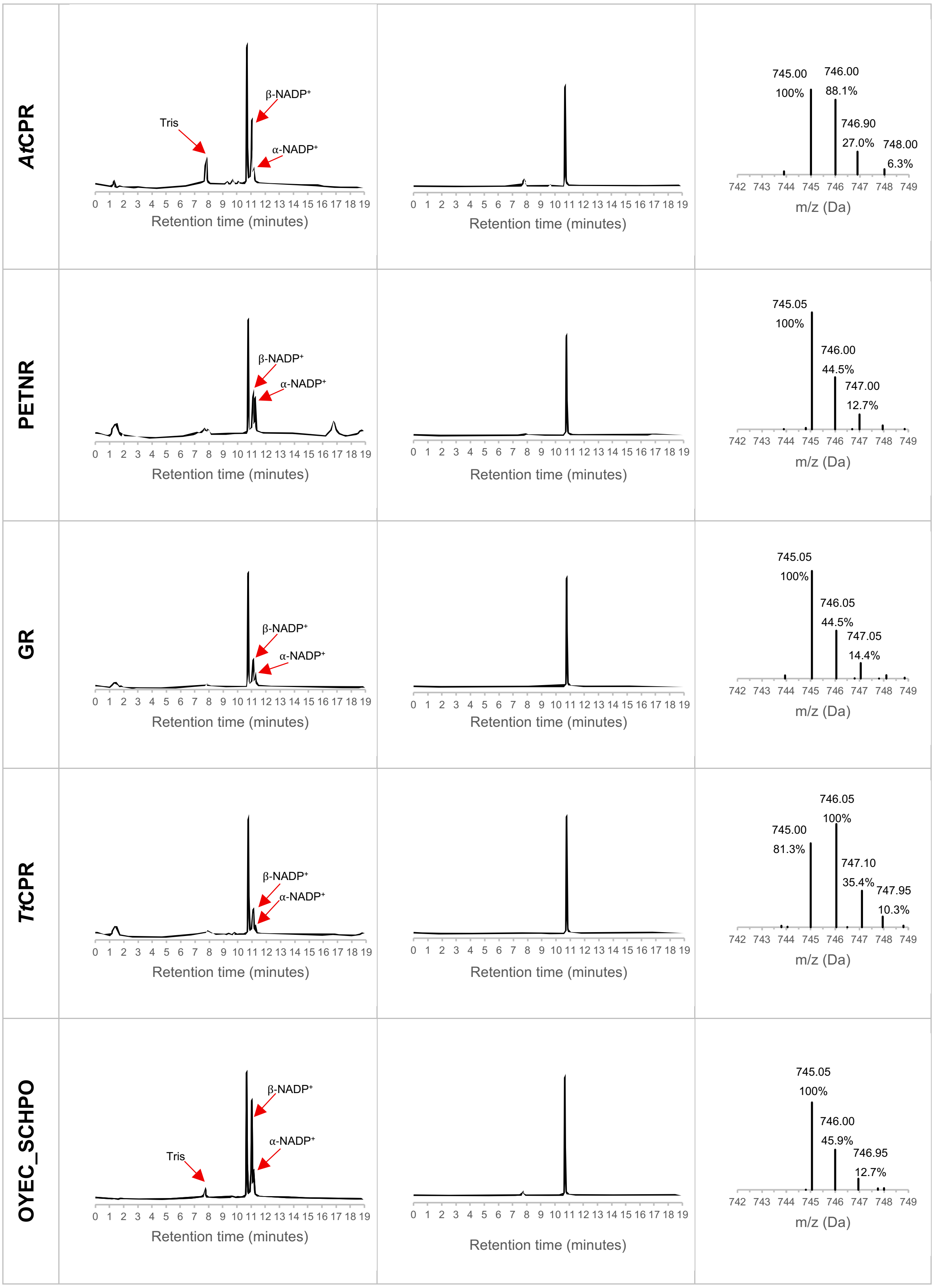
AtCPR
PETNR
GR
TtCPR
OYEC_SCHPO
Tris
β-NADP⁺
α-NADP⁺
0 1 2 3 4 5 6 7 8 9 10 11 12 13 14 15 16 17 18 19
Retention time (minutes)
742 743 744 745 746 747 748 749
m/z (Da)
745.00
100%
746.00
88.1%
746.90
27.0%
748.00
6.3%
745.05
100%
746.00
44.5%
747.00
12.7%
745.05
100%
746.05
44.5%
747.05
14.4%
746.05
100%
745.00
81.3%
747.10
35.4%
747.95
10.3%
745.05
100%
746.00
45.9%
746.95
12.7%

<table>
<tr><td>YqiG_BACSU</td><td>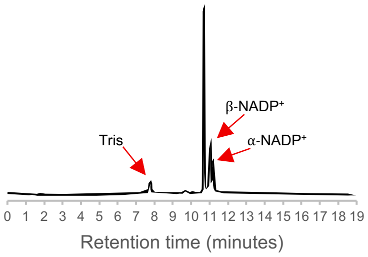
</td><td>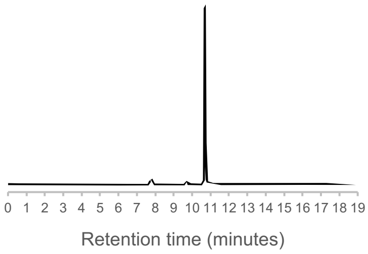
</td><td>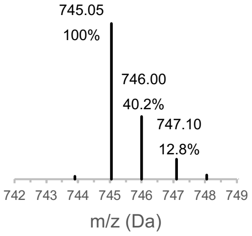
</td></tr>
</table>

Mass spectra correspond to the 1,4-NAD(P)H peak. Peaks labelled with the red arrows (Tris, α-NAD(P)$^+$ and β-NAD(P)$^+$) were assigned by comparison against commercial standards.

S5.2.2. UPLC-MS data – HIE of NADH

<table>
<tr><th></th><th>UV absorbance at 260 nm</th><th>UV absorbance at 340 nm</th><th>Mass detection (ESI) negative ion mode</th></tr>
<tr><td>β-NAD$^+$ standard</td><td>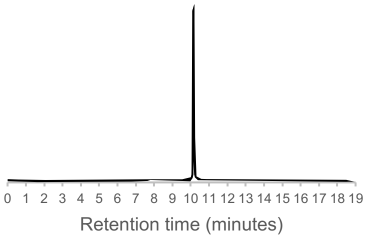
</td><td>-</td><td>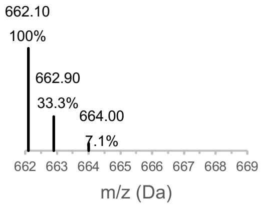
</td></tr>
<tr><td>1,4-NADH standard</td><td>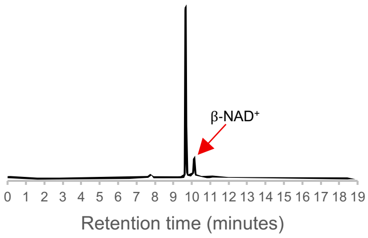
</td><td>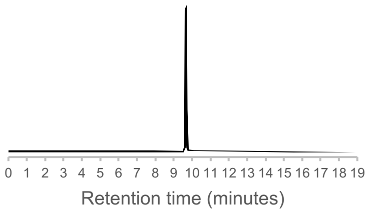
</td><td>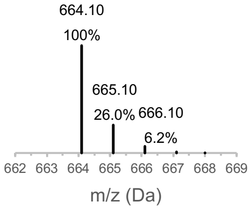
</td></tr>
<tr><td>MR</td><td>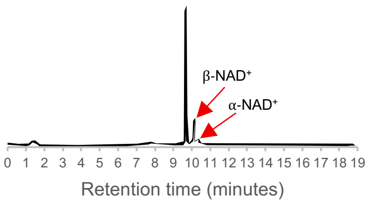
</td><td>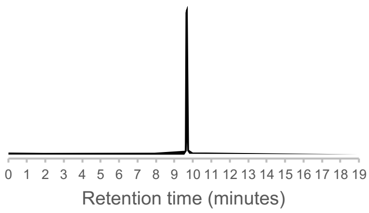
</td><td>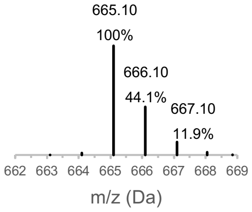
</td></tr>
</table>

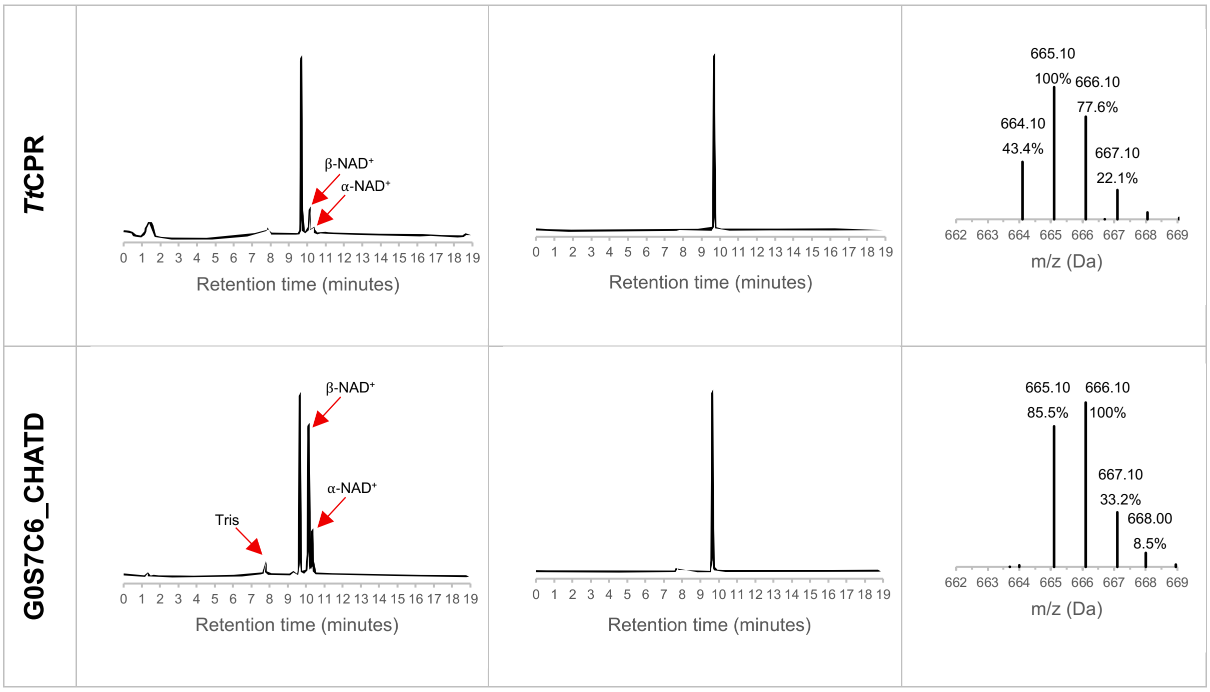

Mass spectra correspond to the 1,4-NAD(P)H peak. Peaks labelled with the red arrows (Tris, α-NAD(P)$^+$ and β-NAD(P)$^+$) were assigned by comparison against commercial standards.

S5.2.3. ^{1}H NMR (400 MHz, ^2H_2O, p^{2}H 8.4, 298 K) and UV-vis data – HIE of NADPH

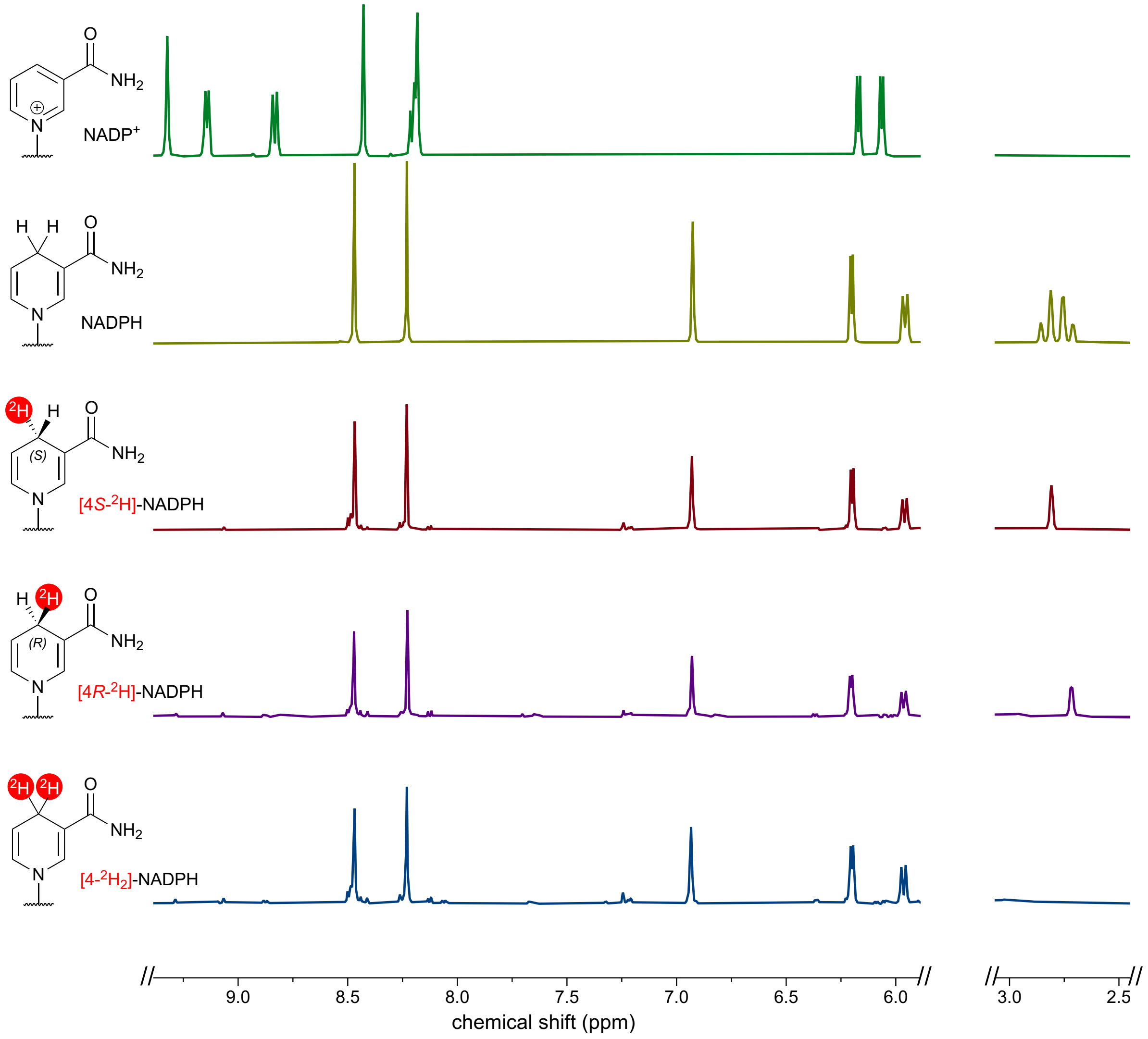

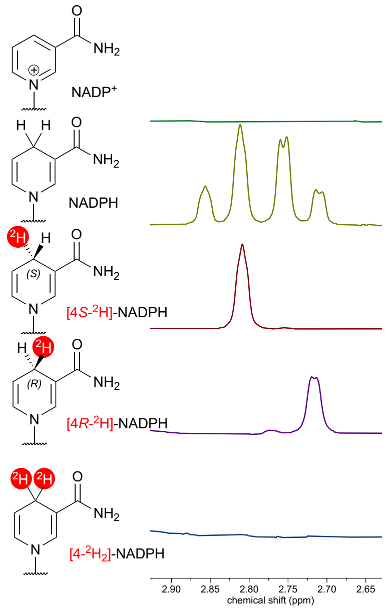

NADPH incubated in $^{1}H_{2}O$ with no enzyme

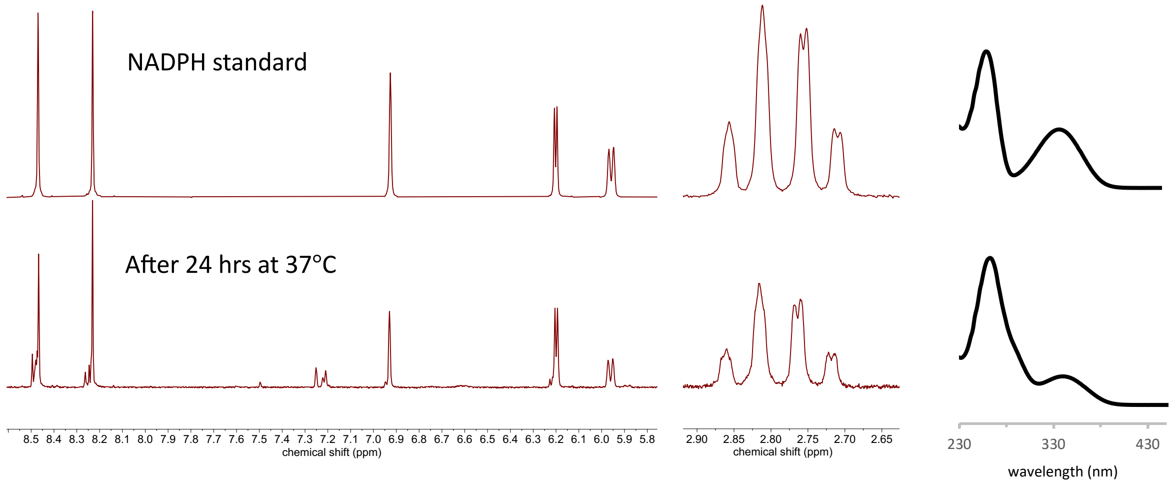

NADPH incubated in $^{1}H_{2}O$ with GR

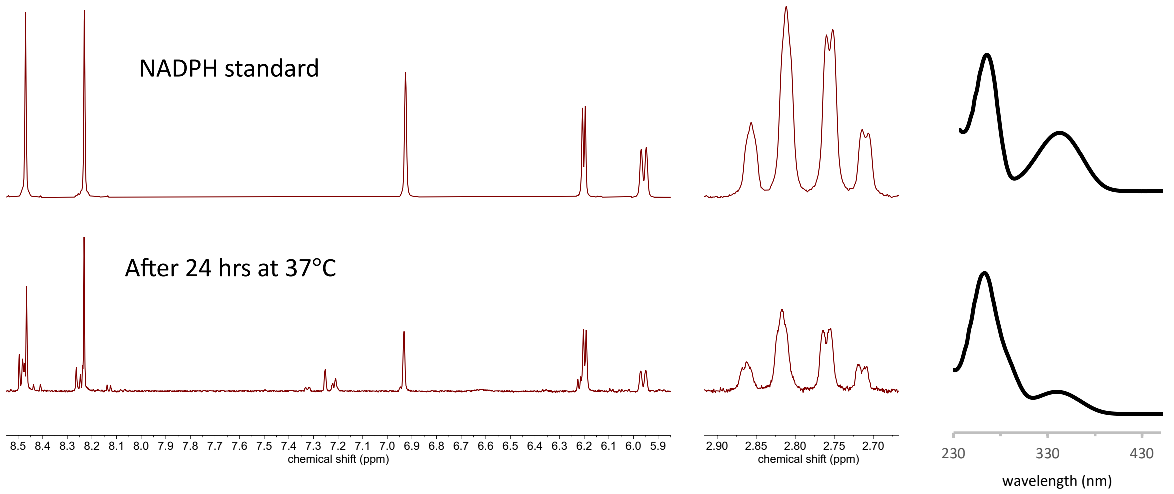

NADPH incubated in $^{2}H_{2}O$ with no enzyme

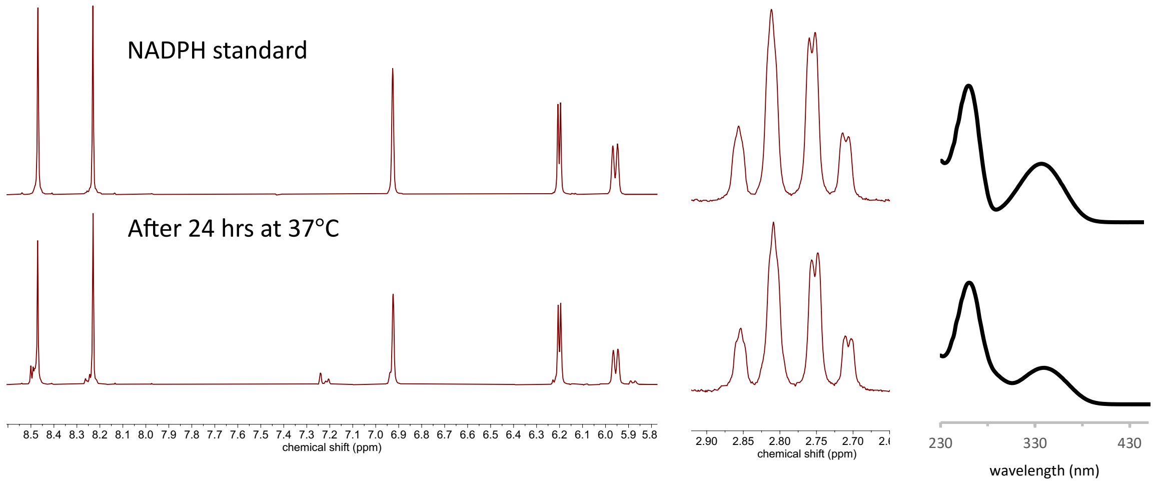

NADPH incubated in $^{2}H_2O$ with FAD

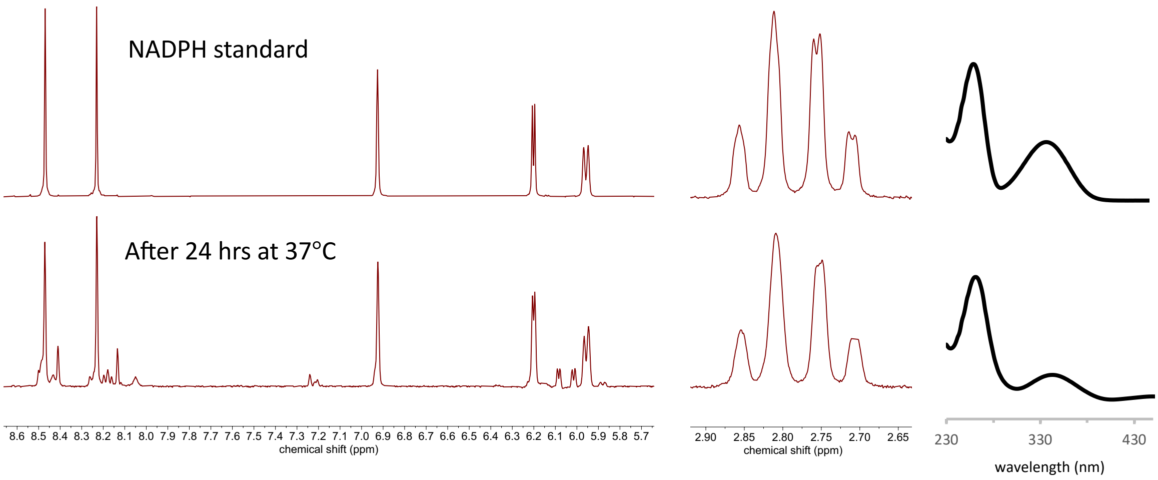

*At*CPR-catalysed HIE of NADPH

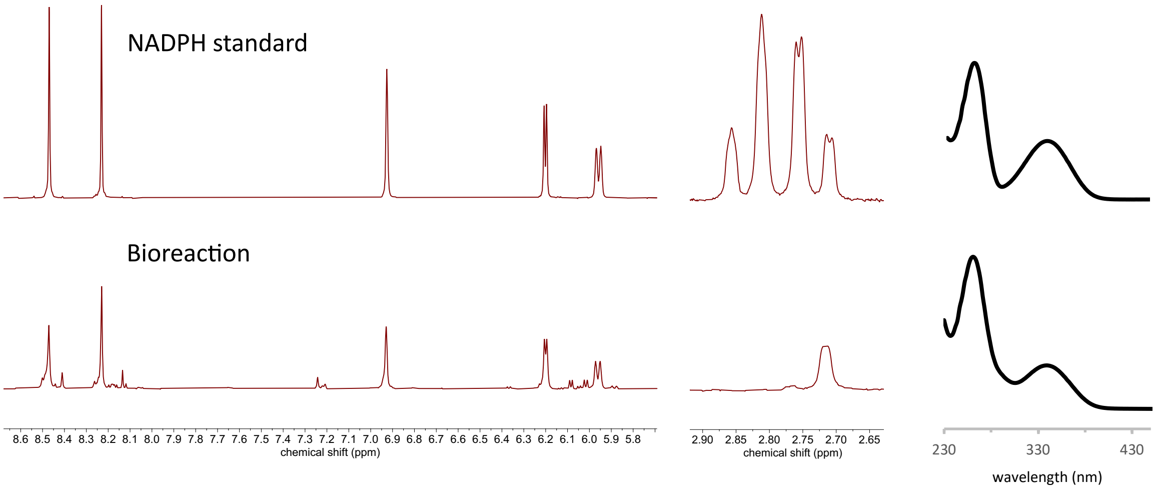

*Tt*CPR-catalysed HIE of NADPH

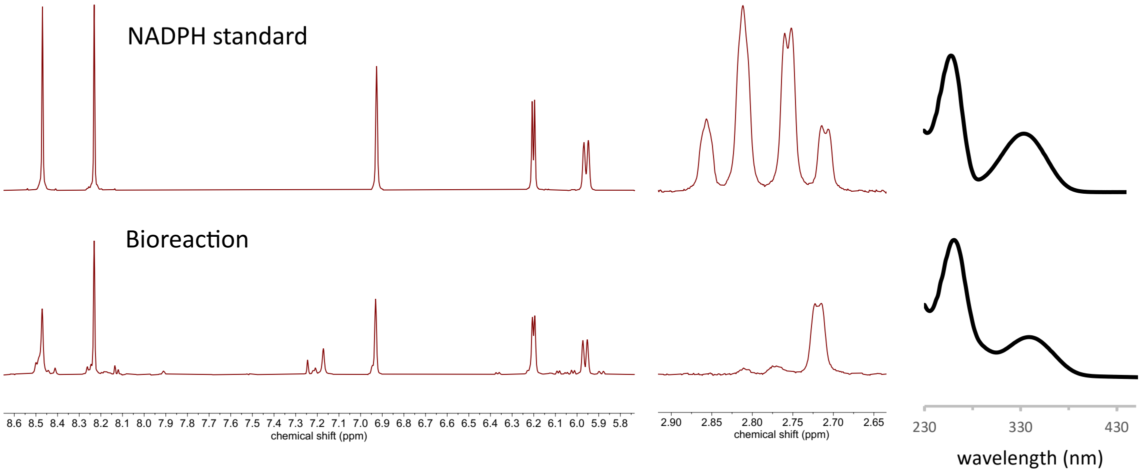

PETNR-catalysed HIE of NADPH

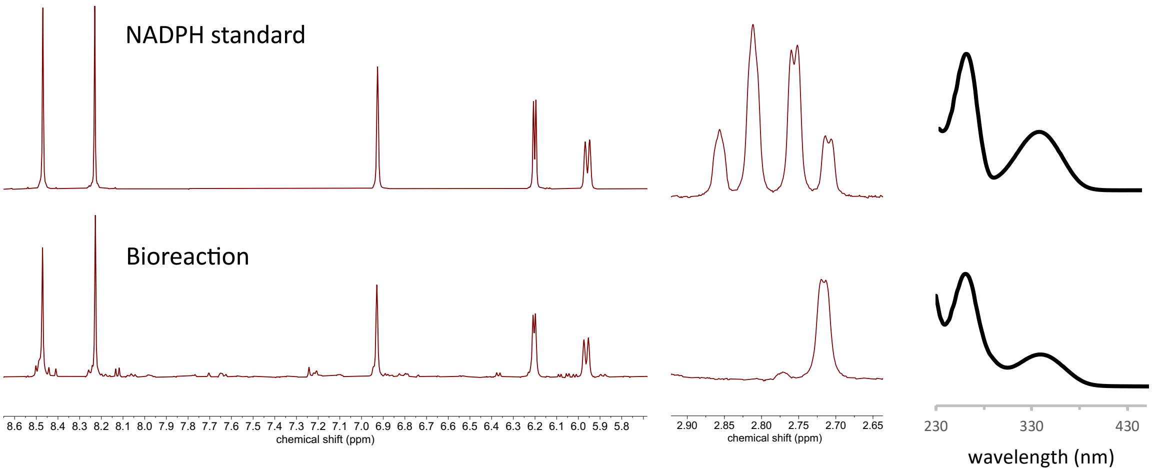

GR-catalysed HIE of NADPH

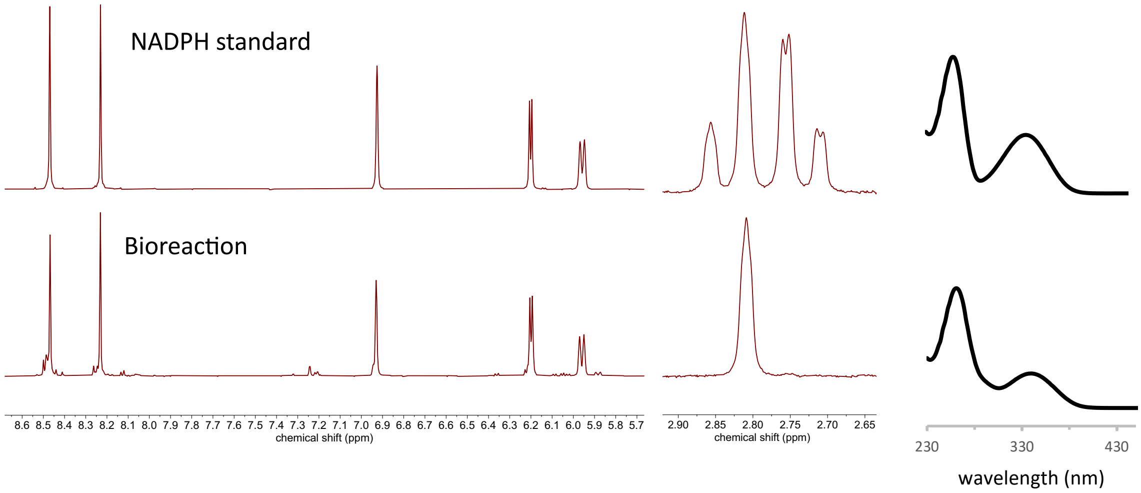

OYEC_SCHPO-catalysed HIE of NADPH

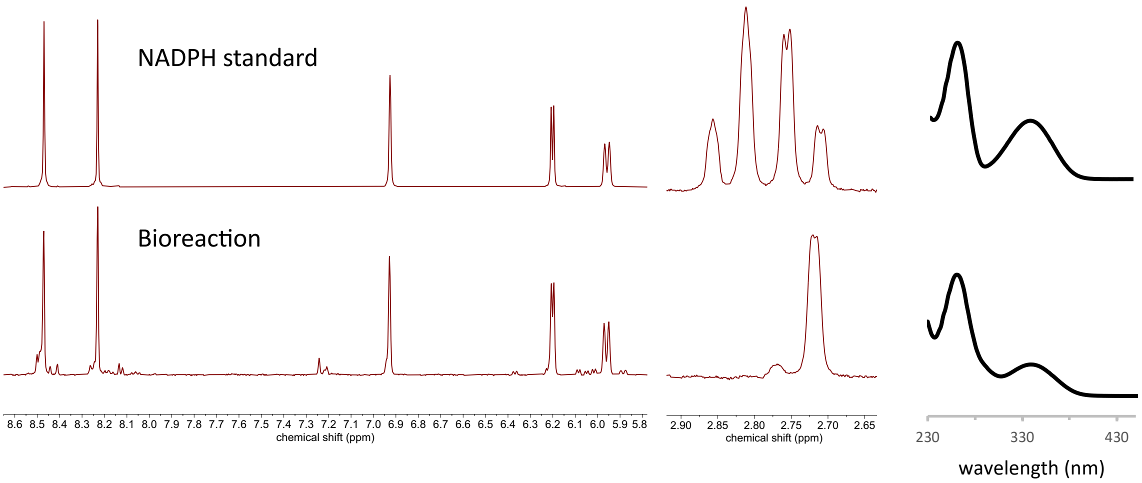

YqiG_BACSU-catalysed HIE of NADPH

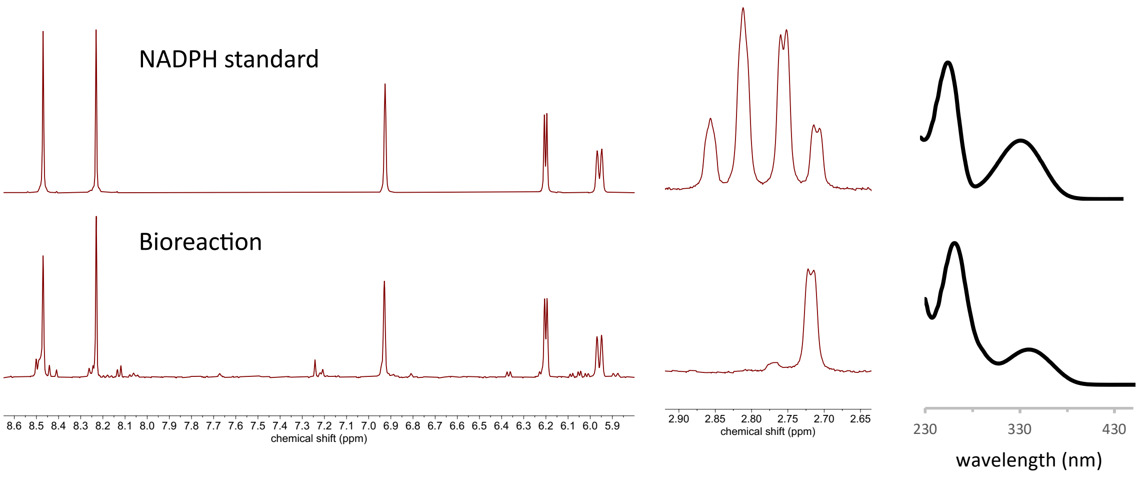

PETNR + GR-catalysed HIE of NADPH

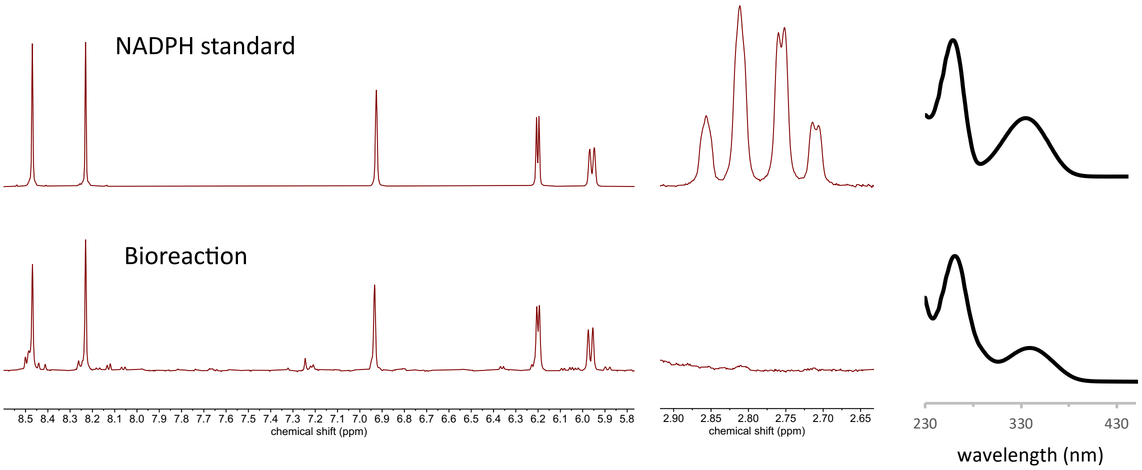

S5.2.4. ^{1}H NMR (400 MHz, ^2H_2O, p^{2}H 8.4, 298 K) and UV-vis data – HIE of NADH

NADH incubated in ^1H_2O with no enzyme

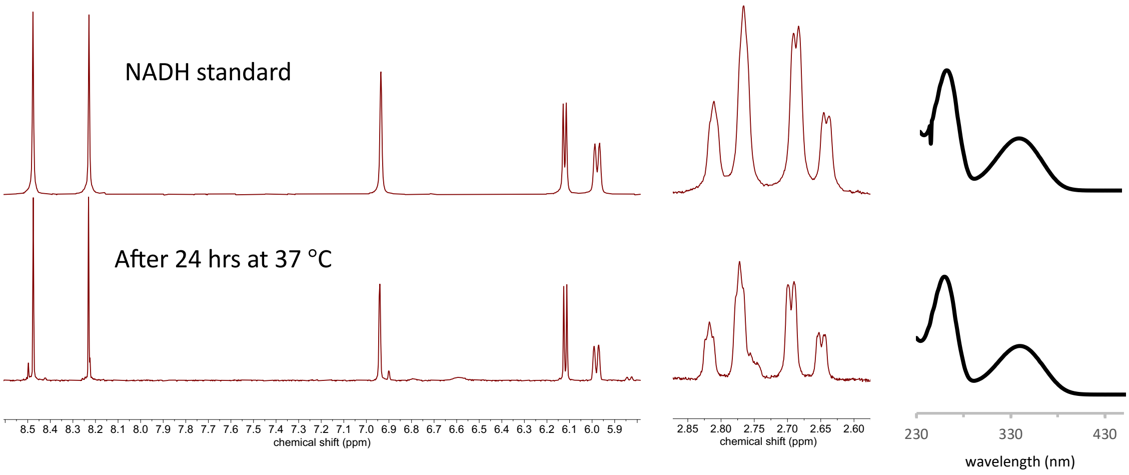

NADH incubated in $^{1}H_2O$ with MR

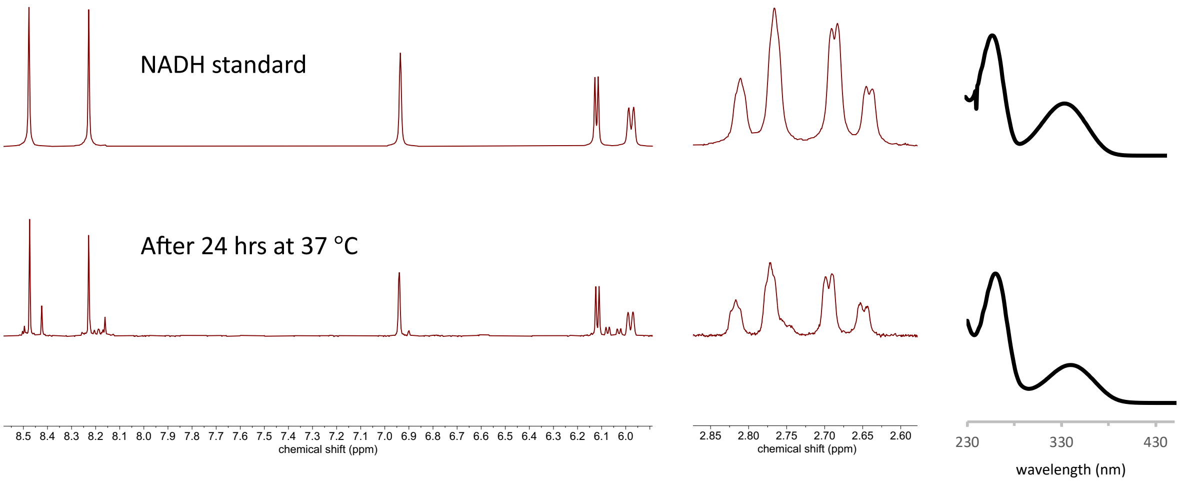

NADH incubated in $^{2}H_2O$ with no enzyme

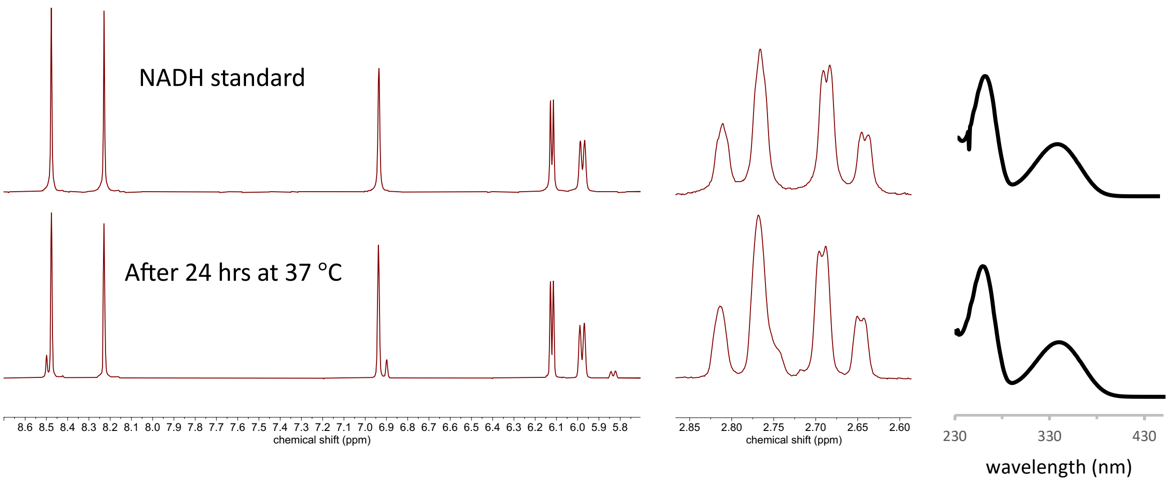

NADH incubated in $^{2}H_2O$ with FAD

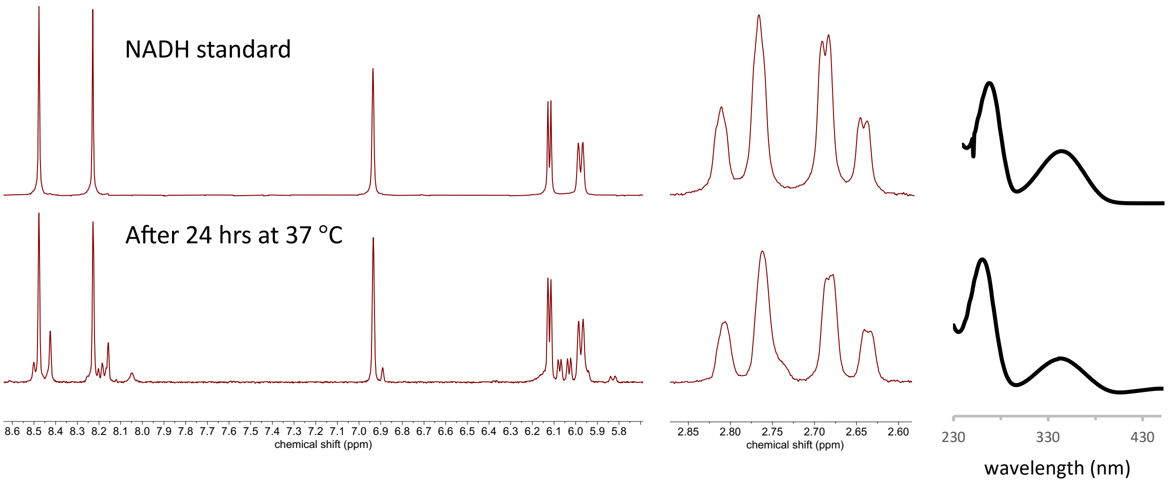

*Tt*CPR-catalysed HIE of NADH

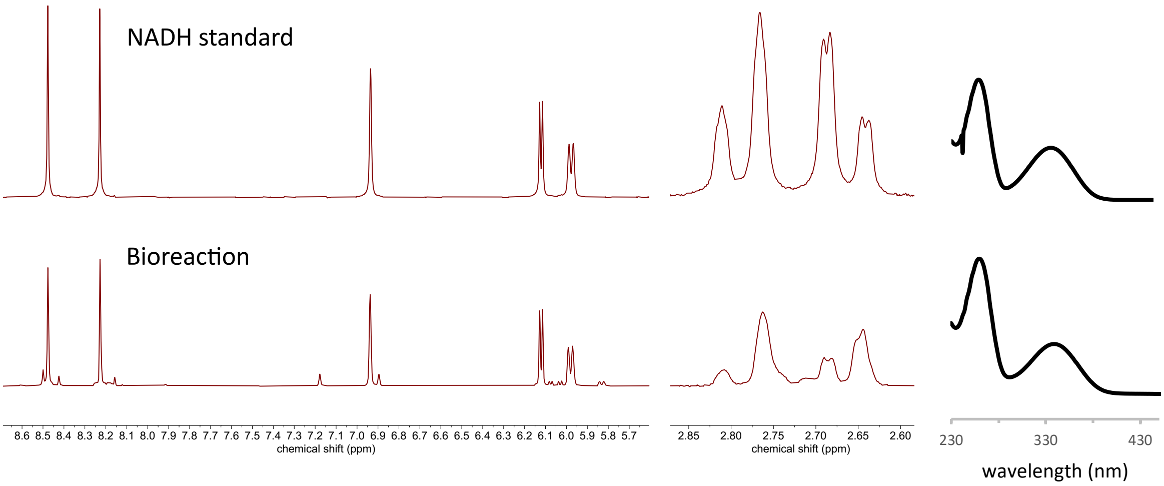

MR-catalysed HIE of NADH

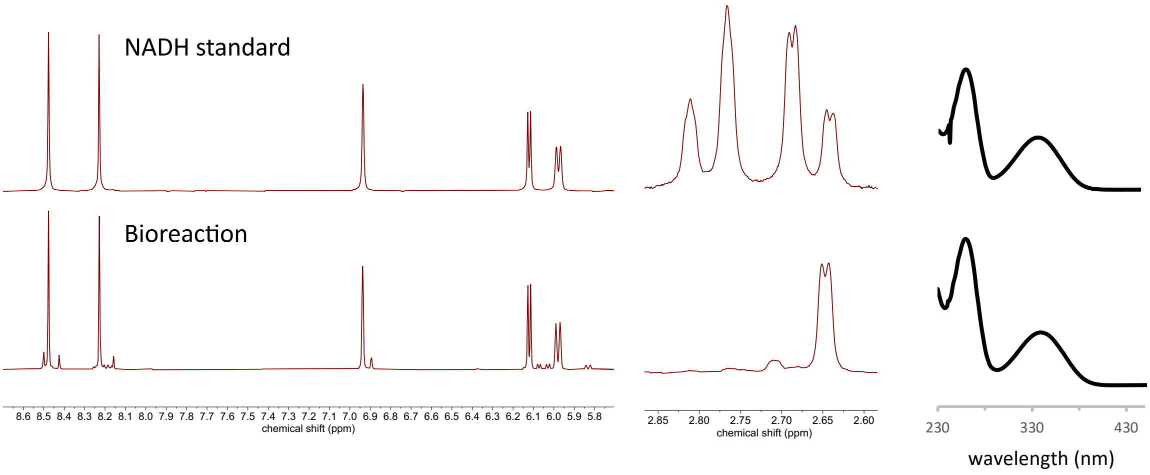

G0S7C6_CHATD-catalysed HIE of NADH

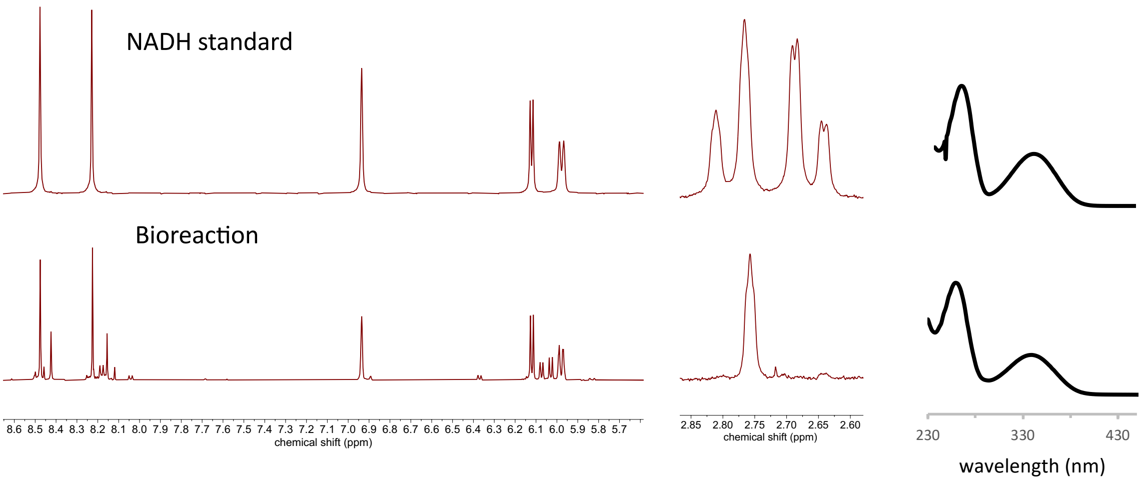

G0S7C6_CHATD + MR-catalysed HIE of NADH

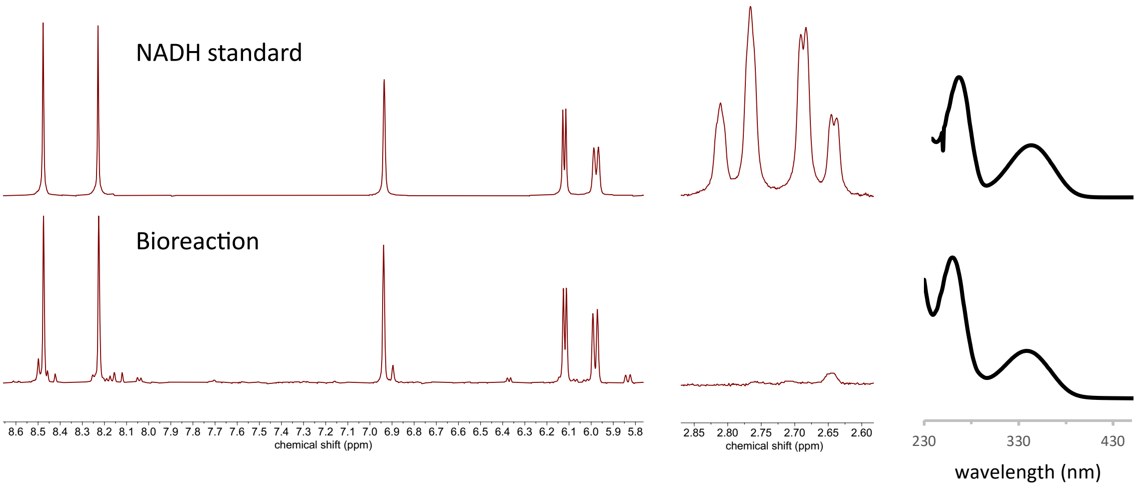

S5.3. Preparative-scale HIE reactions

S5.3.1. UPLC-MS data

	UV absorbance at 260 nm	Mass detection (ESI) negative ion mode
**NADP$^+$ standard**	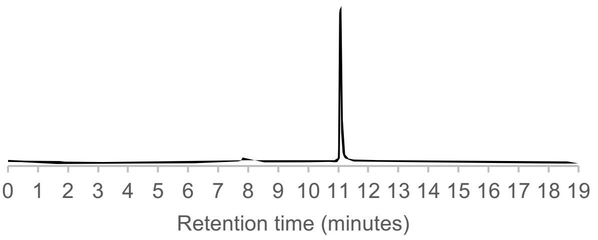	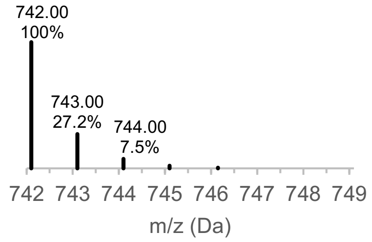
**NADPH standard**	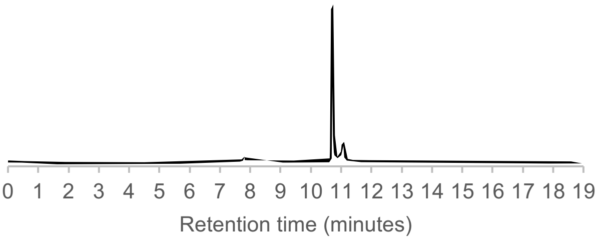	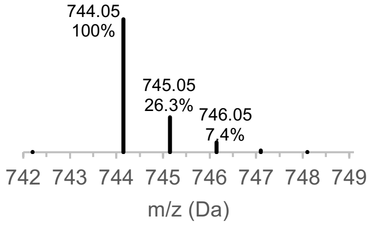
**[4*R*-^{2}H]-NADPH**	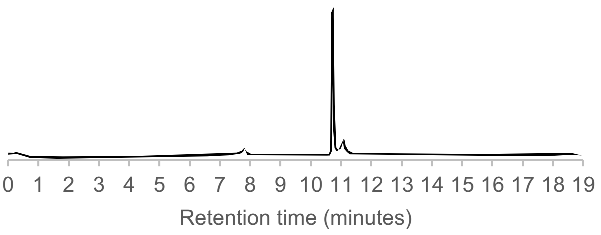	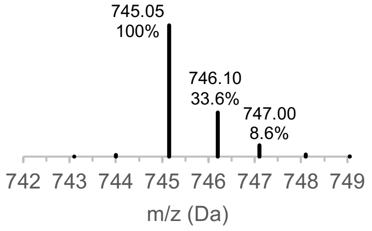

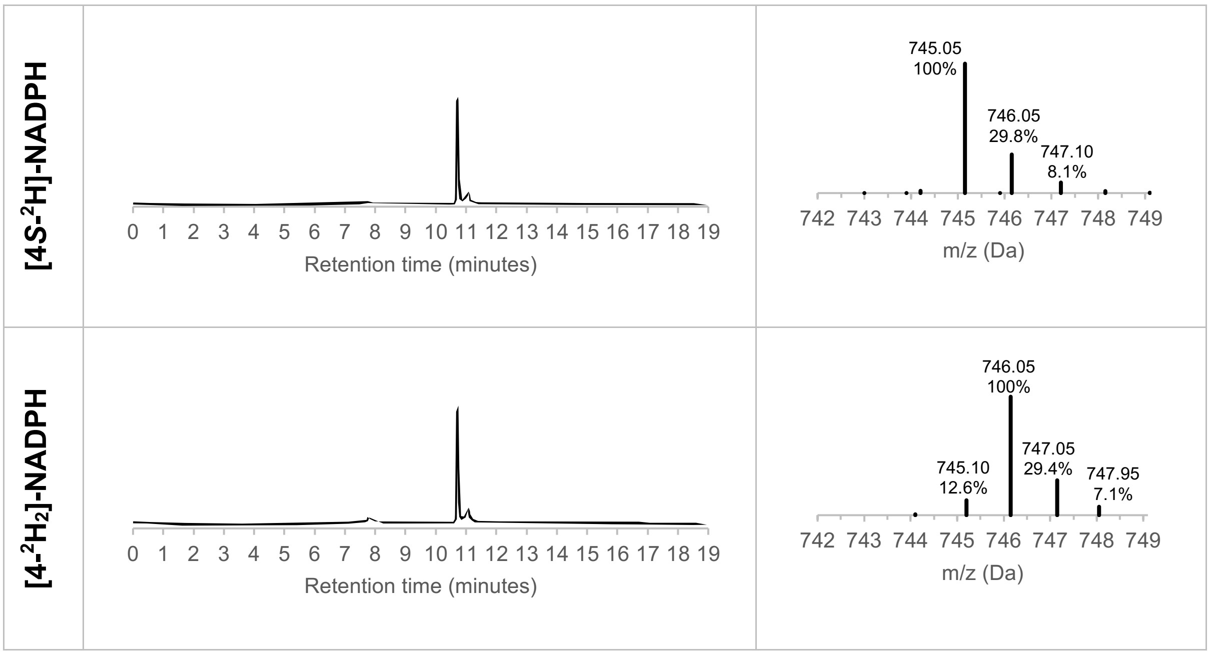

S5.3.2. UV-vis data

Measured in 10 mM Tris-HCl, pH 8.0 at room temperature:

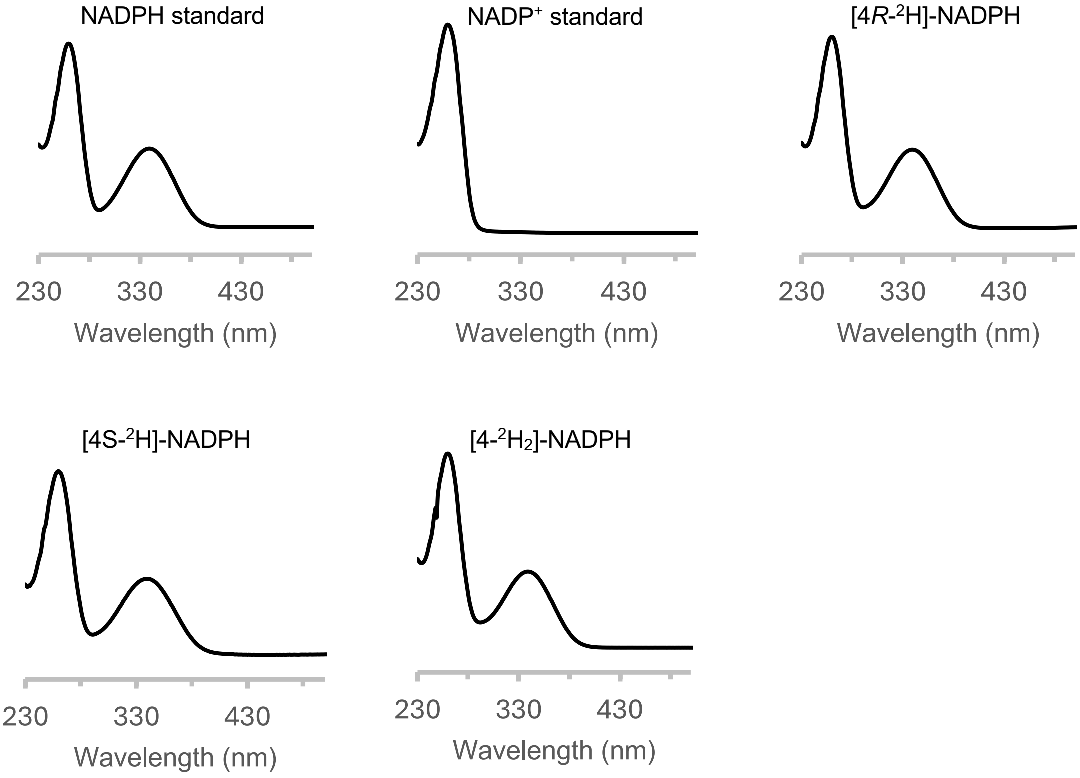

S5.3.3. ^1H NMR data (400 MHz, ^2H_2O, p^{2}H 8.0, 298 K)

In all cases of preparative-scale HIE, 1,6-NADPH formation was ~5%.

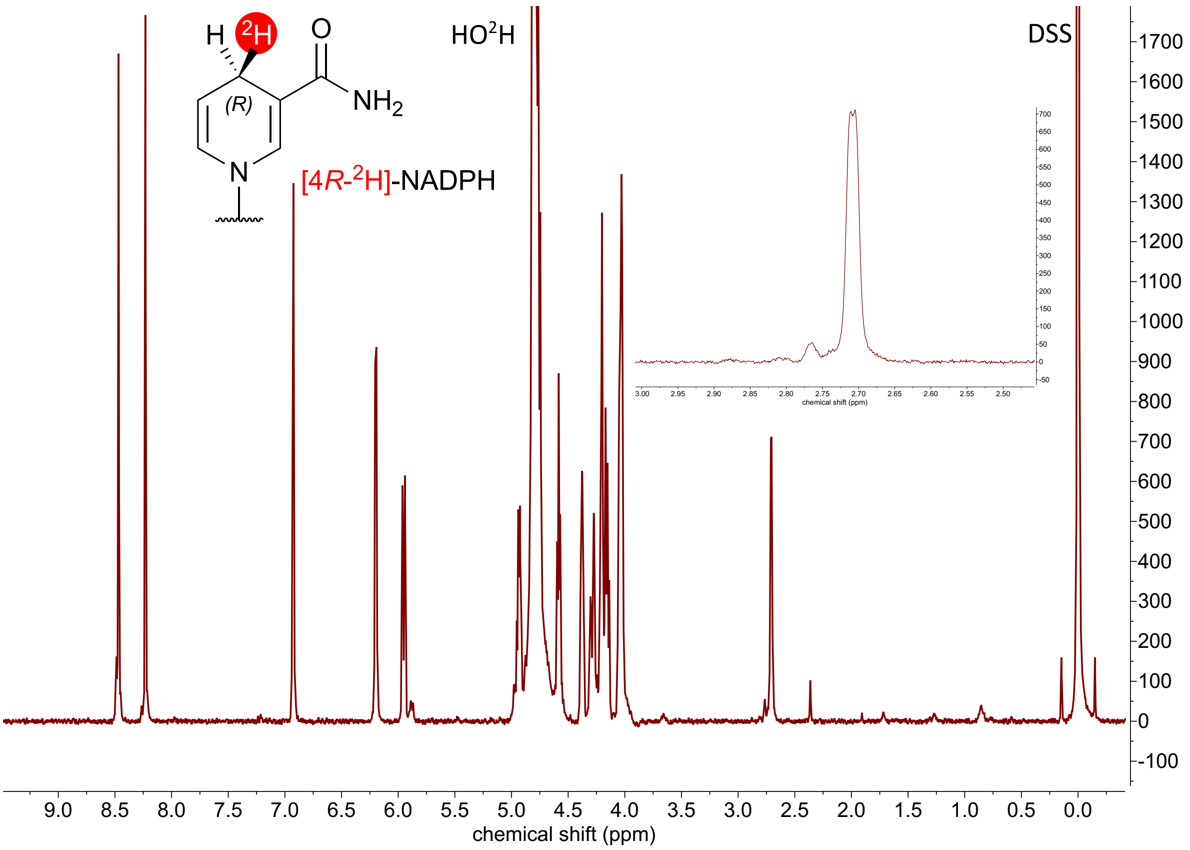

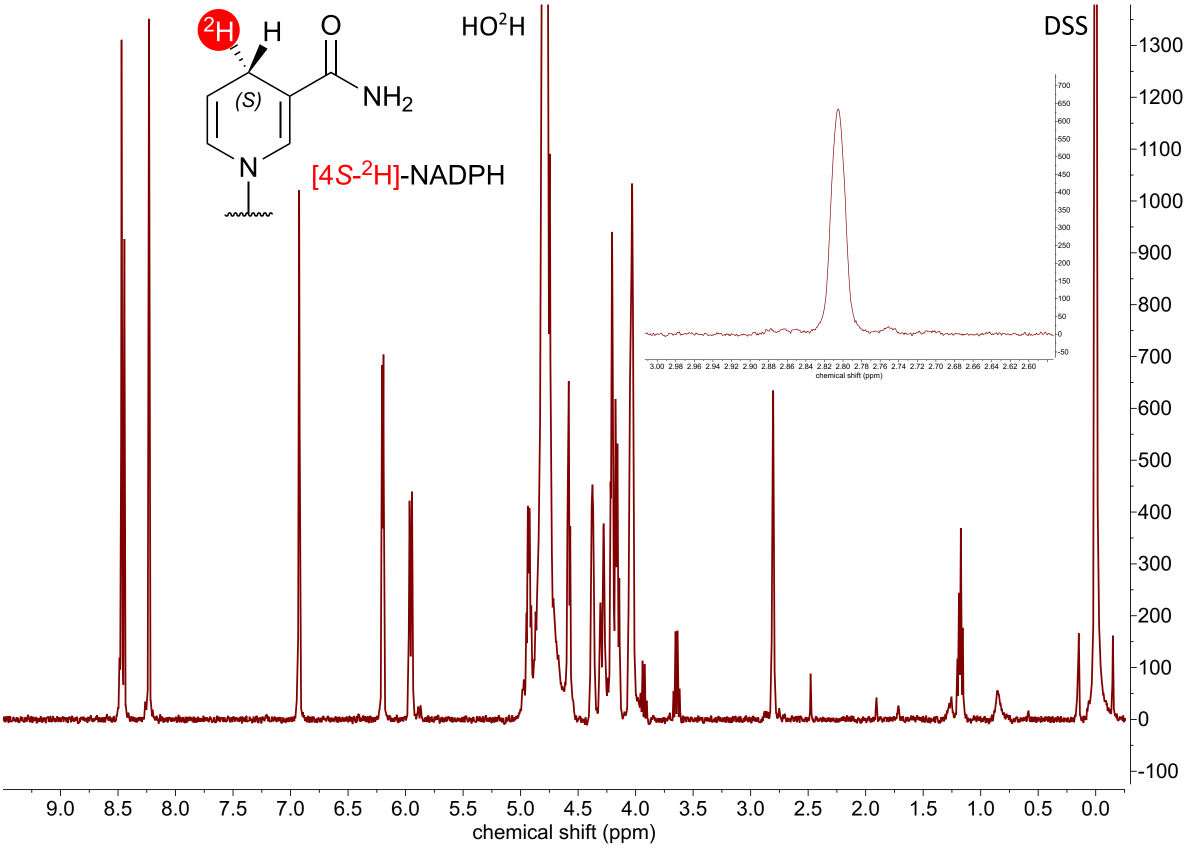

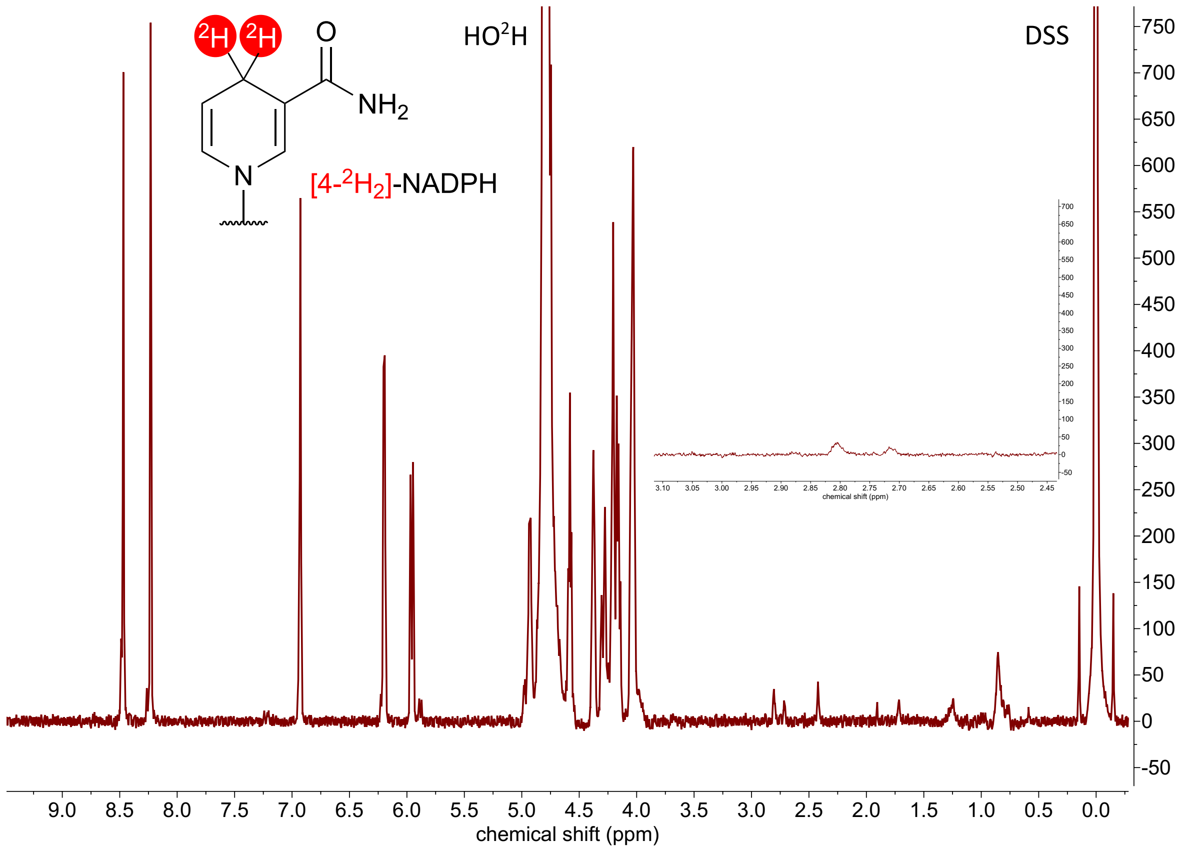

S6. Supplementary References

1. S. K. Chapman and G. A. Reid, *Flavoprotein Protocols*, Humana Press, Totowa, New Jersey, 1999.
2. (a) A. I. Iorgu, M. J. Cliff, J. P. Waltho, N. S. Scrutton and S. Hay, *Methods Enzymol.*, 2019, **620**, 145-166; (b) H. S. Toogood, A. Fryszkowska, M. Hulley, M. Sakuma, D. Mansell, G. M. Stephens, J. M. Gardiner and N. S. Scrutton, *ChemBioChem*, 2011, **12**, 738-749.
3. (a) S. B. Mostad and A. Glasfeld, *J. Chem. Educ.*, 1993, **70**, 504 - 506; (b) J. A. Birrell and J. Hirst, *Biochem.*, 2013, **52**, 4048-4055.
4. J. S. Rowbotham, H. A. Reeve and K. A. Vincent, *ACS Catal.*, 2021, **11**, 2596-2604.
5. J. Basran, R. J. Harris, M. J. Sutcliffe and N. S. Scrutton, *J. Biol. Chem.*, 2003, **278**, 43973-43982.
6. J. Abramson, J. Adler, J. Dunger, R. Evans, T. Green, A. Pritzel, O. Ronneberger, L. Willmore, A. J. Ballard, J. Bambrick, S. W. Bodenstein, D. A. Evans, C. C. Hung, M. O'Neill, D. Reiman, K. Tunyasuvunakool, Z. Wu, A. Zemgulyte, E. Arvaniti, C. Beattie, O. Bertolli, A. Bridgland, A. Cherepanov, M. Congreve, A. I. Cowen-Rivers, A. Cowie, M. Figurnov, F. B. Fuchs, H. Gladman, R. Jain, Y. A. Khan, C. M. R. Low, K. Perlin, A. Potapenko, P. Savy, S. Singh, A. Stecula, A. Thillaisundaram, C. Tong, S. Yakneen, E. D. Zhong, M. Zielinski, A.

Zidek, V. Bapst, P. Kohli, M. Jaderberg, D. Hassabis and J. M. Jumper, *Nature*, 2024, **630**, 493-500.

7. L. Zhang, Z. Xie, Z. Liu, S. Zhou, L. Ma, W. Liu, J. W. Huang, T. P. Ko, X. Li, Y. Hu, J. Min, X. Yu, R. T. Guo and C. C. Chen, *Nat. Commun.*, 2020, **11**, 2676.
8. C. R. Pudney, S. Hay, C. Levy, J. Pang, M. J. Sutcliffe, D. Leys and N. S. Scrutton, *J. Am. Chem. Soc.*, 2009, **131**, 17072-17073.
9. R. Anandakrishnan, B. Aguilar and A. V. Onufriev, *Nucleic Acids Res.*, 2012, **40**, W537-541.
10. W. L. Jorgensen, J. Chandrasekhar, J. D. Madura, R. W. Impey and M. L. Klein, *J. Chem. Phys.*, 1983, **79**, 926-935.
11. M. J. Frisch, G. W. Trucks, H. B. Schlegel, G. E. Scuseria, M. A. Robb, J. R. Cheeseman, G. Scalmani, V. Barone, B. Mennucci, G. A. Petersson, H. Nakatsuji, M. Caricato, X. Li, H. P. Hratchian, A. F. Izmaylov, J. Bloino, G. Zheng, J. L. Sonnenberg, M. Hada, M. Ehara, K. Toyota, R. Fukuda, J. Hasegawa, M. Ishida, T. Nakajima, Y. Honda, O. Kitao, H. Nakai, T. Vreven, J. A. Montgomery, Jr., J. E. Peralta, F. Ogliaro, M. Bearpark, J. J. Heyd, E. Brothers, K. N. Kudin, V. N. Staroverov, R. Kobayashi, J. Normand, K. Raghavachari, A. Rendell, J. C. Burant, S. S. Iyengar, J. Tomasi, M. Cossi, N. Rega, J. M. Millam, M. Klene, J. E. Knox, J. B. Cross, V. Bakken, C. Adamo, J. Jaramillo, R. Gomperts, R. E. Stratmann, O. Yazyev, A. J. Austin, R. Cammi, C. Pomelli, J. W. Ochterski, R. L. Martin, K. Morokuma, V. G. Zakrzewski, G. A. Voth, P. Salvador, J. J. Dannenberg, S. Dapprich, A. D. Daniels, Ö. Farkas, J. B. Foresman, J. V. Ortiz, J. Cioslowski and D. J. Fox, *Gaussian 09, Revision D.01*, 2009.
12. (a) W. J. Hehre, R. Ditchfield and J. A. Pople, *J. Chem. Phys.*, 1972, **56**, 2257-2261; (b) P. C. Hariharan and J. A. Pople, *Theoret. Chim. Acta*, 1973, **28**, 213-222; (c) S. H. Vosko, L. Wilk and M. Nusair, *Can. J. Phys.*, 1980, **58**, 1200-1211; (d) C. Lee, W. Yang and R. G. Parr, *Phys. Rev. B Condens. Matter*, 1988, **37**, 785-789; (e) A. D. Becke, *J. Chem. Phys.*, 1993, **98**, 5648-5652; (f) P. J. Stephens, F. J. Devlin, C. F. Chabalowski and M. J. Frisch, *J. Phys. Chem.*, 1994, **98**, 11623-11627.
13. (a) A. T. Carvalho, A. F. Teixeira and M. J. Ramos, *J. Comput. Chem.*, 2013, **34**, 1540-1548; (b) D. A. Case, H. M. Aktulga, K. Belfon, D. S. Cerutti, G. A. Cisneros, V. W. D. Cruzeiro, N. Forouzesh, T. J. Giese, A. W. Gotz, H. Gohlke, S. Izadi, K. Kasavajhala, M. C. Kaymak, E. King, T. Kurtzman, T. S. Lee, P. Li, J. Liu, T. Luchko, R. Luo, M. Manathunga, M. R. Machado, H. M. Nguyen, K. A. O'Hearn, A. V. Onufriev, F. Pan, S. Pantano, R. Qi, A. Rahnamoun, A. Risheh, S. Schott-Verdugo, A. Shajan, J. Swails, J. Wang, H. Wei, X. Wu, Y. Wu, S. Zhang, S. Zhao, Q. Zhu, T. E. Cheatham, 3rd, D. R. Roe, A. Roitberg, C. Simmerling, D. M. York, M. C. Nagan and K. M. Merz, Jr., *J. Chem. Inf. Model.*, 2023, **63**, 6183-6191.
14. J. A. Maier, C. Martinez, K. Kasavajhala, L. Wickstrom, K. E. Hauser and C. Simmerling, *J. Chem. Theory. Comput.*, 2015, **11**, 3696-3713.
15. M. R. Shirts, C. Klein, J. M. Swails, J. Yin, M. K. Gilson, D. L. Mobley, D. A. Case and E. D. Zhong, *J. Comput. Aided Mol. Des.*, 2017, **31**, 147-161.
16. M. J. Abraham, T. Murtola, R. Schulz, S. Páll, J. C. Smith, B. Hess and E. Lindahl, *SoftwareX*, 2015, **1-2**, 19-25.
17. (a) H. J. C. Berendsen, J. P. M. Postma, W. F. van Gunsteren, A. DiNola and J. R. Haak, *J. Chem. Phys.*, 1984, **81**, 3684-3690; (b) G. Bussi, D. Donadio and M. Parrinello, *J. Chem. Phys.*, 2007, **126**, 014101.

18. G. M. Torrie and J. P. Valleau, *J. Compt. Phys.*, 1977, **23**, 187-199.
19. X. Daura, K. Gademann, B. Jaun, D. Seebach, W. F. van Gunsteren and A. E. Mark, *Angew. Chem. Int. Ed.*, 1999, **38**, 236-240.
20. T. Saba, J. W. H. Burnett, J. Li, P. N. Kechagiopoulos and X. Wang, *Chem. Commun.*, 2020, **56**, 1231-1234.
21. C. Bernofsky and S. Y. Wanda, *J. Biol. Chem.*, 1982, **257**, 6809-6817.
22. (a) S. Chaykin and L. Meissner, *Biochem. Biophys. Res. Commun.*, 1964, **14**, 233-240; (b) S. Chaykin, L. King and J. G. Watson, *Biochemica et Biophysica Acta*, 1966, **124**, 13-25.
23. H. Jaegfeldt, *Bioelectrochemistry and Bioenergetics*, 1981, **128**, 355-370.
24. (a) T. Saba, J. Li, J. W. H. Burnett, R. F. Howe, P. N. Kechagiopoulos and X. Wang, *ACS Catal.*, 2020, **11**, 283-289; (b) F. Liu, C. Ding, S. Tian, S. M. Lu, C. Feng, D. Tu, Y. Liu, W. Wang and C. Li, *Chem. Sci.*, 2022, **13**, 13361-13367; (c) C. Trotta, G. Menendez Rodriguez, C. Zuccaccia and A. Macchioni, *ACS Catal.*, 2024, **14**, 10334-10343; (d) M. A. S. Al-Shaibani, T. Sakoleva, L. A. Zivkovic, H. P. Austin, M. Dorr, L. Hilfert, E. Haak, U. T. Bornscheuer and T. Vidakovic-Koch, *ChemistryOpen*, 2024, **13**, e202400064.
25. X. Wang, T. Saba, H. H. P. Yiu, R. F. Howe, J. A. Anderson and J. Shi, *Chem*, 2017, **2**, 621-654.
26. (a) S. E. Godtfredsen, M. Ottesen and N. R. Andersen, *Carlsberg Res. Commun.*, 1979, **44**, 65 - 75; (b) M. V. Makarov, F. Hayat, B. Graves, M. Sonavane, E. A. Salter, A. Wierzbicki, N. R. Gassman and M. E. Migaud, *ACS Chem. Biol.*, 2021, **16**, 604-614.
27. (a) N. Esaki, H. Shimoi, N. Nakajima, T. Ohshima, H. Tanaka and K. Soda, *J. Biol. Chem.*, 1989, **264**, 9750-9752; (b) A. Pennacchio, A. Giordano, L. Esposito, E. Langella, M. Rossi and C. A. Raia, *Protein Pept. Lett.*, 2010, **17**, 437-443; (c) K. Yoneda, H. Sakuraba, T. Araki and T. Ohshima, *FEBS Open Bio*, 2021, **11**, 1981-1986.
